# Safety and Immunogenicity of an AMA-1 Malaria Vaccine in Malian Adults: Results of a Phase 1 Randomized Controlled Trial

**DOI:** 10.1371/journal.pone.0001465

**Published:** 2008-01-23

**Authors:** Mahamadou A. Thera, Ogobara K. Doumbo, Drissa Coulibaly, Dapa A. Diallo, Abdoulaye K. Kone, Ando B. Guindo, Karim Traore, Alassane Dicko, Issaka Sagara, Mahamadou S. Sissoko, Mounirou Baby, Mady Sissoko, Issa Diarra, Amadou Niangaly, Amagana Dolo, Modibo Daou, Sory I. Diawara, D. Gray Heppner, V. Ann Stewart, Evelina Angov, Elke S. Bergmann-Leitner, David E. Lanar, Sheetij Dutta, Lorraine Soisson, Carter L. Diggs, Amanda Leach, Alex Owusu, Marie-Claude Dubois, Joe Cohen, Jason N. Nixon, Aric Gregson, Shannon L. Takala, Kirsten E. Lyke, Christopher V. Plowe

**Affiliations:** 1 Malaria Research and Training Center, University of Bamako, Bamako, Mali; 2 Division of Malaria Vaccine Development, Walter Reed Army Institute of Research, Silver Spring, Maryland, United States of America; 3 Malaria Vaccine Development Program, U.S. Agency for International Development, Washington, D. C., United States of America; 4 World Wide Clinical Development – Malaria Vaccines, GlaxoSmithKline Biologicals, Rixensart, Belgium; 5 Center for Vaccine Development, University of Maryland School of Medicine, Baltimore, Maryland, United States of America; London School of Hygiene & Tropical Medicine, United Kingdom

## Abstract

**Background:**

The objective was to evaluate the safety, reactogenicity and immunogenicity of the AMA-1-based blood-stage malaria vaccine FMP2.1/AS02A in adults exposed to seasonal malaria.

**Methodology/Principal Findings:**

A phase 1 double blind randomized controlled dose escalation trial was conducted in Bandiagara, Mali, West Africa, a rural town with intense seasonal transmission of *Plasmodium falciparum* malaria. The malaria vaccine FMP2.1/AS02A is a recombinant protein (FMP2.1) based on apical membrane antigen-1 (AMA-1) from the 3D7 clone of *P. falciparum*, adjuvanted with AS02A. The comparator vaccine was a cell-culture rabies virus vaccine (RabAvert). Sixty healthy, malaria-experienced adults aged 18–55 y were recruited into 2 cohorts and randomized to receive either a half dose or full dose of the malaria vaccine (FMP2.1 25 µg/AS02A 0.25 mL or FMP2.1 50 µg/AS02A 0.5 mL) or rabies vaccine given in 3 doses at 0, 1 and 2 mo, and were followed for 1 y. Solicited symptoms were assessed for 7 d and unsolicited symptoms for 30 d after each vaccination. Serious adverse events were assessed throughout the study. Titers of anti-AMA-1 antibodies were measured by ELISA and *P. falciparum* growth inhibition assays were performed on sera collected at pre- and post-vaccination time points. Transient local pain and swelling were common and more frequent in both malaria vaccine dosage groups than in the comparator group. Anti-AMA-1 antibodies increased significantly in both malaria vaccine groups, peaking at nearly 5-fold and more than 6-fold higher than baseline in the half-dose and full-dose groups, respectively.

**Conclusion/Significance:**

The FMP2.1/AS02A vaccine had a good safety profile, was well-tolerated, and was highly immunogenic in malaria-exposed adults. This malaria vaccine is being evaluated in Phase 1 and 2 trials in children at this site.

**Trial Registration:**

ClinicalTrials.gov NCT00308061

## Introduction

*Plasmodium falciparum* malaria remains a major global killer, especially of infants and children, and a serious threat to travelers. A safe and effective malaria vaccine used in conjunction with other control measures would be a huge boon to the health and economies of malaria-endemic countries. In recent clinical trials, RTS,S/AS02, a recombinant subunit protein malaria vaccine designed to block infection, demonstrated 35% efficacy against uncomplicated malaria and 49% efficacy against severe malaria for at least 18 months in young children and 66% efficacy against *P. falciparum* infection in infants [1]–[3]. Approaches to improve on this efficacy include building multi-stage, multi-antigen vaccines [4], combination with a viral vector [5] and developing more-effective single antigen or live attenuated vaccines [6], [7].

Apical membrane antigen-1 (AMA-1) is a 83-kilodalton surface protein that is expressed by mature intra-erythrocytic malaria parasites and processed to a 66-kilodalton protein before being exported to the merozoite surface around the time of rupture of the infected erythrocyte [8]. Several lines of evidence including in vitro growth inhibition assays [9]–[12], antibody-mediated inhibition of antigen processing [13], and sero-epidemiological surveys [14], [15] support a critical role for AMA-1 during merozoite invasion of erythrocytes. A vaccine that boosts levels of anti-AMA-1 antibodies might therefore reduce the risk that malaria infection will cause clinical disease.

AMA-1 is highly polymorphic [16], [17]. Polymorphisms in AMA-1 allow the parasite to evade antibody-mediated inhibition of invasion in vitro [18], and sera from rabbits immunized with different forms of AMA-1 showed limited cross-protection, with the level of inhibition inversely related to the number of amino acid differences between parasite strains [12], [19], [20]. The relevance, if any, of these in vitro and animal studies for allele-specific efficacy of AMA-1 vaccines in humans is unknown. Presently three AMA-1-based adjuvanted protein vaccines are being evaluated in clinical trials in Mali, including two different monovalent vaccines based on AMA-1 derived from the 3D7 and FVO clones of *P. falciparum*, respectively, [20], [21]and a bivalent vaccine that includes both of these versions of AMA-1 [22].

Falciparum Malaria Protein 2.1 (FMP2.1) is a recombinant AMA-1 from the 3D7 clone of *P. falciparum* that is produced in and purified from *Escherichia coli* [23]. Together with the AS02A adjuvant system, an oil-in-water emulsion with the immunostimulants monophosphoryl lipid A and QS 21, it constitutes the FMP2.1/AS02A malaria vaccine. This vaccine has been evaluated in a Phase 1 dose escalation clinical trial in malaria-naive North American adults [21]. The vaccine was well tolerated and strongly immunogenic, inducing both humoral and cellular immune responses. Vaccine-induced antibodies also inhibited parasite growth and interfered with antigen processing in vitro. Because previous exposure to malaria may affect the reactogenicity and immunogenicity of malaria vaccines, we conducted a Phase 1 dose escalation trial of this vaccine in malaria-experienced adults in Mali. A cell-culture rabies virus vaccine was used as a comparator to help distinguish vaccine-induced immune responses from natural background immunity. This study was the first evaluation of FMP2.1/AS02A in a malaria-experienced population and the antecedent to Phase 1 and Phase 2 clinical trials of this vaccine in children that are now in progress at this site.

## Methods

The protocol for this trial and supporting CONSORT checklist are available as supporting information; see Checklist S1 and Protocol S1.

### Study Setting

The study was conducted at the Bandiagara Malaria Project research clinic adjacent to the district hospital in Bandiagara, a rural town of 13,634 inhabitants in the Dogon Country in northeast Mali. It is relatively dry, with a mean annual rainfall of 600 mm. *Anopheles gambiae* is the principal malaria vector. Malaria transmission is strictly seasonal, with virtually undetectable transmission at the height of the dry season in March; less than 1 infected bite per person per month as the transmission season starts and ends in June and December, respectively; and a peak of up to 60 infected mosquito bites per person per month in August or September [24], [25]. *P. falciparum* represents 97% of malaria infections with 3% due to *P. malariae* and rare infections with *P. ovale*. Despite the seasonal transmission pattern the malaria burden is heavy: children aged less then 10 years have an average of 2 clinical malaria episodes every transmission season [25] and severe malaria afflicts 1 in 50 children aged less than 6 years each year [24]. Older children and adults are relatively protected against malaria disease but remain susceptible to malaria infection.

### Participants

After obtaining community permission as described by Diallo et al. [26], the trial was publicized by local radio broadcast. Men and women aged 18 to 55 years were invited to the research clinic to be screened for eligibility. Participants were included if they had resided in Bandiagara for at least 12 months, gave written informed consent, and, if female, declared their intent not to become pregnant during the first 3 months of the study (up to one month following the third immunization). Exclusion criteria included: current illness as indicated by history, examination and/or laboratory testing, previous immunization with a rabies vaccine or any experimental vaccine, recent use of immunosuppressants, receipt of blood products during the previous 6 months, pregnancy or breast-feeding, alcohol or drug abuse, and allergy to substances present in the vaccines.

#### Ethical compliance

The trial was conducted in compliance with the International Conference on Harmonization Good Clinical Practices, the Declaration of Helsinki and regulatory requirements of Mali. The protocol was approved by institutional review boards of the University of Bamako Faculty of Medicine, University of Maryland Baltimore, and the U.S. Army Surgeon General. Separate written informed consent was obtained for screening and for enrollment. Consent of illiterate participants was documented by their thumbprints and by signatures of independent witnesses. Permission to import and administer the investigational products in Mali was granted by the Republic of Mali Ministry of Health. The trial was monitored by the United States Army Medical Materiel Development Activity and the National Institute of Allergy and Infectious Diseases/Division of Microbiology and Infectious Diseases.

### Interventions

The FMP2.1 antigen (Lot 0971) is comprised of amino acids #83-531 corresponding to the ectodomain of AMA-1 derived from the 3D7 clone of *P. falciparum*. The protein was produced in and purified from *E. coli* bacteria under current Good Manufacturing Practices at the Walter Reed Army Institute of Research Pilot Bioproduction facility (Forest Glen, Maryland, United States) [23]. The vaccine was provided in vials containing approximately 50 µg of lyophilized protein.

The AS02A adjuvant system is composed of an oil-in-water emulsion and two immuno-stimulants, 3-deacylated monophosphoryl lipid A and QS21, a saponin agent derived from the soap bark tree, *Quillaja saponaria* [27], [28]. AS02A was manufactured by GlaxoSmithKline Biologicals (Rixensart, Belgium) according to current Good Manufacturing Practices P and provided in pre-filled syringes. The whole content of each FMP2.1 vial was dissolved in the whole content of a separate 0.62 mL vial of AS02A immediately before injection. The RabAvert rabies vaccine (Chiron Corporation, Emeryville, California, United States) is a sterile freeze-dried vaccine obtained by growing the fixed-virus strain Flury LEP in primary cultures of chicken fibroblasts. It is supplied in pre-filled syringes containing lyophilized antigen to which 1 mL of sterile water was added as a diluent before injection. All vaccines were administered by intramuscular injection in the left deltoid muscle.

Sixty adults were sequentially assigned to 2 cohorts of 30. Within each cohort, participants were randomized in a 2∶1 fashion to receive FMP2.1/AS02A or rabies vaccine. After reconstitution, the dose of FMP2.1/AS02A was approximately 25 µg of FMP2.1 in a final volume of 0.25 mL AS02A in Cohort 1 (half dose), and approximately 50 µg FMP2.1 in a final volume of 0.5 mL in Cohort 2 (full dose). Vaccines were given on a 0-, 1- and 2-mo schedule. The first vaccination was given in early December 2004 at the end of the malaria transmission season; the second and the third doses were given in January and February 2005, when virtually no malaria transmission occurs at this site. Study day 90 was in March 2005, at the nadir of malaria transmission, study day 180 was at the beginning of the malaria season, and study day 272 was in September, at the peak of malaria transmission intensity. The final study follow-up on day 364 coincided with the end of the 2005 malaria season. The cohorts were immunized in a staggered fashion to permit interim safety analyses; each successive immunization of Cohort 1 was followed in approximately 3 wk by the corresponding immunization of Cohort 2. Three interim safety analyses were reviewed by an independent Safety Monitoring Committee, which provided written recommendations to proceed before each of the three immunizations of Cohort 2 with the full dose of FMP2.1/AS02A.

### Objectives

The primary objective was to evaluate the safety and reactogenicity of 3 injections of 2 different dose levels of the malaria vaccine FMP2.1/AS02A in malaria-experienced Malian adults. Secondary objectives were to measure the magnitude and duration of antibody responses to FMP2.1, and exploratory objectives included measuring vaccine-induced cellular immune responses at baseline and after immunization (results to be presented elsewhere); and measuring the inhibition of parasite growth by in vitro growth inhibition assays.

### Outcomes

The primary outcome was safety, measured as 1) occurrence of solicited symptoms during an 8-day follow-up period after immunization (day of immunization and days 1, 2, 3 and 7 after immunization); 2) occurrence of unsolicited symptoms during a 31-day follow-up period after immunization (day of immunization and 30 subsequent days); 3) occurrence of laboratory toxicities during the study period; and 4) occurrence of serious adverse events during the study period. Secondary outcomes were anti-AMA-1 antibody titers measured against recombinant 3D7 AMA-1 and at baseline and at specified times during and after immunization. Serum inhibition of parasite growth in vitro was an exploratory outcome.

#### Assessment of safety and tolerability

Following each immunization, participants were directly observed for 30 minutes, then evaluated at the study clinic 1, 2, 3, 7 and 14 days after each immunization and on study days 90, 180, 272 and 364. Starting on day 120, monthly home visits were made to check the health status of participants and to encourage them to come to the research clinic if they felt ill. Study physicians were available at the research clinic at all times throughout the 12-month study to assess and treat adverse events.

Clinical evaluations consisted of measurement of vital signs and assessment for local injection site and general solicited signs or symptoms. Local signs and solicited symptoms included pain, swelling, erythema at the injection site and limitation of arm abduction at the shoulder. General signs and solicited symptoms included fever (oral temperature≥37.5°C), chills, nausea, headache, malaise, myalgia and joint pain. Any other signs or symptoms were considered to be unsolicited, as were all signs or symptoms that occurred more than 7 days after immunization. Solicited symptoms were considered to be related to the study vaccines. Unsolicited signs and symptoms were recorded during the 30 days after each immunization, whereas serious adverse events and pregnancies were monitored throughout the 12-month study.

Blood was collected at screening, on immunization days, 7 and 14 days after each immunization and on study days 90, 180, 272 and 364 to determine complete blood count, alanine aminotransferase (ALT) and serum creatinine.

Adverse events were graded by severity and judged for relatedness to study vaccines. Grade 1 adverse events were easily tolerated, causing minimal discomfort and not interfering with daily activities. Grade 2 adverse events were sufficiently discomforting to interfere with normal activities. Grade 3 adverse events prevented normal daily activities. Swelling, erythema, fever and limitation of arm motion had specific definitions not based on interference with daily activities. Injection site swelling and erythema were graded based on their widest dimension: Grade 1, >0 to 20 mm; Grade 2, >20 to 50 mm; and Grade 3, >50 mm. Fever was classified as Grade 3 if the oral temperature was ≥39°C whereas Grade 3 limitation of arm motion was classified as abduction limited to 30°. For laboratory tests, toxicity grading was adapted to normal reference ranges determined for the local adult population.

#### Antibody responses to AMA-1

Antibody responses to AMA-1 were measured by an enzyme-linked immunosorbent assay (ELISA) [21,21]. Briefly, IgG ELISAs were performed using FMP2.1 as the capture antigen, in serial 2-fold dilution, and the titer was defined as the serum dilution required to yield and optical density of 1.0 in our assay. Antibody responses were measured on serum obtained from participants at the time of each immunization (study days 0 [baseline], 30 and 60), 2 wk after each immunization (study days 14, 44 and 74), and 1, 3, 6 and 10 mo after the scheduled time of the last immunization (study days 90, 180, 272 and 364).

#### Growth invasion/inhibition assay

Pre-immunization (day 0) sera and sera from 2 wk after the third immunization (day 74, corresponding to peak antibody titers) were tested for growth inhibitory effects against homologous (derived from 3D7 clone) and heterologous (derived from FVO clone) *P. falciparum* parasites as described [21], [29]. Sera were dialyzed using 12–14 kilodalton molecular weight cutoff membranes against three rounds of 1× PBS and one round of RPMI 1640 [30]. Samples were then heat-inactivated for 20 min at 56°C and pre-absorbed with erythrocytes from the same donor as the erythrocytes in which parasites had been cultured (2.5 µl of erythrocytes at 50% hematocrit for 50 µl serum). Samples were tested at 20% serum concentration in 384-well plates (Perkin Elmer Spectra 384-TC plates, Cat #6007650) against 3D7 or FVO parasites (2% hematocrit, 0.3% starting parasitemia). Assays were initiated with parasites synchronized at schizont stage and harvested 40 hrs (3D7) or 44 hrs (FVO) after culture setup, i.e., after one cycle. Growth inhibition was determined by measuring parasite lactate dehydrogenase as described [19] and reported as percent growth inhibition relative to control. Growth inhibition assays were performed in a double blind manner.

Detailed methods and results of assays for cellular immune responses will be described in a separate publication.

### Sample size

A sample size of 20 in each group was chosen to balance the need to detect any possible untoward reactions against the need to limit the number of volunteers involved for safety purposes. This Phase 1 trial was thus not powered to detect differences between groups and where comparative statistics for the safety variables were computed, the study had power to detect only large differences in the incidence of local and general side effects between the vaccination groups. Incorporation of a comparator vaccine group of 20 permitted broad initial estimates of the incidence of local and general side effects and of immune responses among vaccine recipients.

### Randomization—Sequence generation

Within the two cohorts, individual participants were randomized in a 2∶1 ratio to receive either FMP2.1/AS02A (half dose in Cohort 1 and full dose in Cohort 2) or rabies vaccine. The randomization sequence was generated by a computer program using blocks of three to ensure a 2∶1 ratio of vaccine allocation. Randomly generated sequential codes linked each study number to a vaccine assignment (FMP2.1/AS02A or rabies vaccine).

### Randomization—Allocation concealment

The randomization sequence was provided by the study statistician consultant in an opaque sealed envelope to the study pharmacists. In addition the local safety monitor was provided with a sealed envelope to be opened if it was deemed necessary to determine urgently the intervention a participant had received; no such emergency unblinding occurred. The only people at the study site with access to the randomization codes during the study were 2 study pharmacists, who had no contact with study participants and did not reveal vaccine assignments to anyone else. Study participants and investigators who assessed outcomes were blinded to vaccine assignment.

### Randomization—Implementation

Clinical investigators assigned study numbers to participants of each group in the order in which they arrived at the clinic on the first day of immunization. At the time of the first immunization, study pharmacists opened the sealed envelopes containing the vaccine assignment and prepared the vaccine to be administered to the respective study participant. The vaccine and dose assigned during the first immunization were maintained for second and third immunizations. The study pharmacists prepared the vaccines in a special room with access strictly limited to them and to study monitors. Syringes containing the prepared vaccines were passed through small sliding doors from the vaccine preparation room to separate vaccine administration rooms, where the immunizations were administered.

### Blinding

The reconstituted rabies vaccine was a clear to slightly opaque, colorless suspension of 1 mL volume, while FMP2.1/AS02A was off-white and either 0.25 or 0.5 mL in volume. Syringes containing vaccines were covered with opaque tape to conceal their content from participants and immunizers. The study pharmacists, who were unblinded, had no study-related contact with participants and were not involved in outcome assessment. Because of the difference in volumes, the immunizers could potentially have deduced which vaccine was given to a specific participant, and therefore they did not participate in other study procedures. The presence of both study pharmacists and immunizers at the site was limited to the periods during which immunizations were given, and these individuals were instructed not to discuss vaccine allocation with other study staff.

### Statistical methods

Adverse event rates were analyzed using SPSS version 11.1 (SPSS, Chicago, Illinois, United States). Fisher's exact test was used to compare rates between vaccine groups. Confidence intervals for geometric mean AMA-1 antibody titers were estimated by using log_10_-transformed values, calculating the 95% confidence interval based on the normal distribution, and then converting the limits to the original scale for presentation. Wilcoxon Signed Rank Test was used to evaluate vaccine effects on parasite growth inhibition on paired samples from study days 0 and 74, for each study group. The differences in mean parasite growth inhibition for the three study groups on study day 74 was evaluated by using a One Way ANOVA and adjusted for multiple comparisons using Tukey's post test on pairwise comparisons using Jandel SigmaStat version 2.0 (Corte Madera, CA, United States). For longitudinal analysis of antibody responses, log-transformed antibody titers were modeled using mixed spline models to determine the effect of vaccine dose on mean antibody levels over time using SAS version 9.1 (Cary, NC, United States). The spline models consisted of a linear function joined to a quadratic function at study day 74. A spatial exponential covariance structure was used to model the correlation between measurements from the same individual taking into account the number of study days between each measurement. Point estimates and confidence intervals for each time point were generated based on the fitted longitudinal model, using the ESTIMATE statement in the MIXED procedure in SAS. All tests were 2-sided, and no correction of *p*-values was made for additional analyses. Given the large number of statistical tests performed and the small sample size, the *p*-values have limited probabilistic interpretation. Safety and immunogenicity analyses were based on intention-to-treat, such that all available data were included in analyses including partial data from 2 participants who were subsequently lost to follow-up.

## Results

### Participant flow

**Figure 1:**
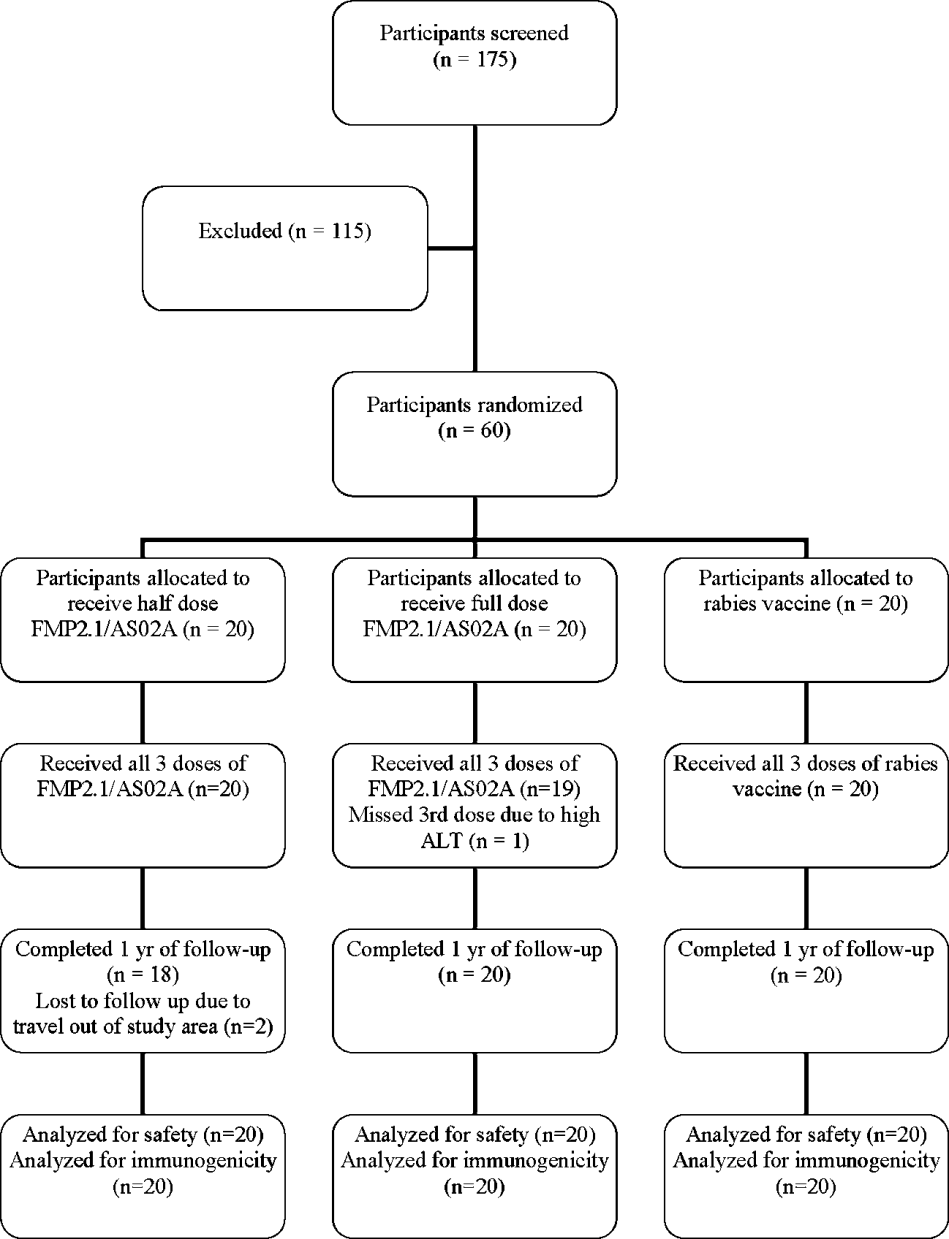
Trial Profile.

One hundred and seventy five persons were screened, and 60 who fulfilled the criteria for inclusion were enrolled in the study (Figure 1). The most common reasons for exclusion were medical illnesses and planned travel out of the study area. After enrollment, one participant traveled outside the study area two days after his second immunization and missed all subsequent visits, and one participant missed the final study visit on day 364 due to travel. Three participants temporarily left the study area to attend a professional meeting, and missed only their study day 63 clinic visits. All of these participants received all 3 doses of vaccine. One participant received only 2 immunizations due to an elevated ALT (described below in Laboratory Safety Tests).

### Recruitment

Participants were recruited from November 19 to December 2, 2004. Immunizations for Cohort 1 began on December 4, 2004 and for Cohort 2 on December 24, 2004. Subsequent immunizations were done at 30-day intervals following this staggered start. Active surveillance of participants for 30 days after each immunization was completed in March 2005, corresponding to study day 90. The database was locked for the primary unblinded analysis after study day 90, and the study continued in a single-blinded fashion, although individual study allocations were not disclosed to on-site study investigators or staff with the exception of the principal investigator. The extended surveillance phase included continuous access to free basic medical care at the research clinic, monthly home visits, and scheduled visits on study days 180, 272 and 364.

### Baseline data

The three study groups did not differ significantly at enrollment with regard to sex, age or laboratory parameters (Table 1). Eleven of 60 participants were female. The mean age was 28 y.

**Table 1:** Baseline Characteristics of FMP2.1/AS02A and Rabies Vaccine groups

Characteristics	FMP2.1/AS02A Half dose (*n* = 20)	FMP2.1/AS02A Full dose (*n* = 20)	Rabies Vaccine (*n* = 20)
Mean age in year (SD)	26.1 (9.2)	29 (11.2)	30.1 (12.2)
Number of Females	4	4	3
Mean WBC×10^3^/µL (SD)	5.4 (1.3)	5.9 (2.2)	6.0 (1.5)
Mean hemoglobin g/dL (SD)	14.4 (1.4)	14.1 (1.6)	14.0 (1.6)
Mean platelets×10^3^/µL (SD)	232.2 (61.3)	252.3 (81.3)	239.3 (68.2)
Mean lymphocytes×10^3^/µL (SD)	1.9 (0.6)	2.3 (0.9)	2.2 (0.8)
Mean creatinine mM/L (SD)	77.8 (14.0)	77.1 (18.6)	76.6 (13.4)
Mean ALT U/L (SD)	18.0 (6.6)	17.3 (7.9)	18.1 (5.7)
GMT Anti-AMA-1 antibody titer (95% CI)	19,161 (8,570–42,844)	23,500 (9,079–60,826)	14,355 (5,860–35,165)

GMT, geometric mean titer; CI, confidence interval; ALT, alanine amino-transferase; SD, standard deviation.

### Numbers analyzed

All available data from all participants, including partial data from participants lost to follow-up, were included in both safety and immunogenicity analyses.

### Safety and reactogenicity

#### Local solicited adverse events

After each immunization, the proportion of participants who had at least one local injection site reaction during the 8-d post-immunization periods was higher and similar in both malaria vaccine groups, compared to the rabies vaccine group (Table 2). Pain and swelling at the injection site were the most common local reactions for all groups and tended to diminish in frequency and severity with successive immunizations. Grade 3 injection site swelling was seen after all immunizations in all three groups, but was much more common in the FMP2.1/AS02A malaria vaccine full-dose group. Swelling classified as Grade 3 occurred in seven participants after each of the three immunizations in that group, compared to one, three and two participants after the first, second and third immunizations, respectively, in the malaria vaccine half-dose group, and in one, three and one participants after the first, second and third immunizations, respectively, in the rabies vaccine group (Table 2). The swelling was typically unnoticed by the participant and detected only on physical examination, and did not interfere with normal daily activities. No other Grade 3 local adverse events occurred. Three episodes of Grade 1 arm motion limitation occurred in the malaria vaccine full-dose group, one after the first immunization and two after the second immunization. Five episodes of arm motion limitation (four Grade 1, one Grade 2) occurred in the malaria vaccine half-dose group, four after the first immunization and one after the second immunization. No episodes of arm motion limitation occurred in the rabies vaccine group. All local solicited symptoms resolved without sequelae during the 8-day post-immunization periods.

**Table 2:** Signs and Solicited Symptoms during the 8-d Follow-Up Periods after Each Immunization

	FMP2.1/AS02A half dose						FMP2.1/AS02A full dose						Rabies Vaccine					
	Immunization 1 (*n* = 20)		Immunization 2 (*n* = 20)		Immunization 3 (*n* = 20)		Immunization 1 (*n* = 20)		Immunization 2 (*n* = 20)		Immunization 3 (*n* = 19)		Immunization 1 (*n* = 20)		Immunization 2 (*n* = 20)		Immunization 3 (*n* = 20)	
	Overall	Grade 3	Overall	Grade 3	Overall	Grade 3	Overall	Grade 3	Overall	Grade 3	Overall	Grade 3	Overall	Grade 3	Overall	Grade 3	Overall	Grade 3
Local																		
Pain (%)	19 (95)	0	15 (75)	0	11 (55)	0	20 (100)	0	19 (95)	0	14 (73.7)	0	6 (30)	0	10 (50)	0	6 (30)	0
Swelling (%)	13 (65)	1 (5)	11 (55)	3 (15)	7 (35)	2 (10)	13 (65)	7 (35)	10 (50)	7 (35)	8 (42.1)	7 (35)	3 (15)	1 (5)	3 (15)	3 (15)	4 (20)	1 (5)
Limitation of arm motion (%)	4 (20)	0	1 (5)	0	0	0	1 (5)	0	2 (10)	0	0	0	0	0	0	0	0	0
Erythema (%)	0	0	0	0	0	0	0	0	0	0	0	0	0	0	0	0	0	0
General																		
Fever (%)	3 (15)	0	1 (5)	0	2 (10)	0	2 (10)	0	1 (5)	0	3 (15.7)	0	0	0	1 (5)	0	0	0
Headache (%)	5 (25)	0	1 (5)	0	1 (5)	0	5 (25)	0	8 (40)	0	4 (21)	0	2 (10)	0	4 (20)	0	3 (15)	0
Joint pain (%)	0	0	0	0	0	0	1 (5)	0	1 (5)	0	0	0	0	0	1 (5)	0	0	0
Myalgia (%)	2 (10)	0	0	0	1 (5)	0	3 (15)	0	1 (5)	0	2 (10.5)	0	0	0	3 (15)	0	0	0
Malaise (%)	3 (15)	0	0	0	1 (5)	0	2 (10)	0	2 (10)	0	1 (5.3)	0	0	0	3 (15)	0	0	0
Nausea (%)	2 (10)	0	1 (5)	0	1 (5)	0	0	0	1 (5)	0	0	0	0	0	1 (5)	0	0	0
Chills (%)	3 (15)	0	1 (5)	0	0	0	1 (5)	0	1 (5)	0	0	0	0	0	1 (5)	0	0	0

#### General solicited adverse events

The malaria vaccine full-dose group had the most general solicited signs and symptoms during the 8-d post-immunization periods, with 14, 15 and 10 general adverse events following the first, second and third immunizations, respectively, compared to 18, 4 and 6 in the half-dose group and 2, 14 and 3 in the rabies vaccine group (Table 2). Headache was the most common general adverse event in all groups, followed by myalgia and malaise. All general solicited adverse events were of Grade 1 or Grade 2 intensity and resolved during the 8-day follow-up period.

#### Unsolicited adverse events

Overall, unsolicited adverse events reported during the 31-d post immunization periods were balanced by group, with 109, 109 and 100 events reported for the malaria vaccine full-dose group, half-dose group and rabies vaccine group, respectively. The most frequent unsolicited adverse events were upper respiratory tract infections and headaches, followed by traumatic injuries, infections and other common medical problems. All unsolicited adverse events were of Grade 1 or Grade 2 intensity and all resolved during the study period.

One female participant in the rabies vaccine group had a positive urine pregnancy test on study day 180. She subsequently reported that she had terminated the pregnancy by elective abortion. On study day 364, the last day of the study follow-up, her pregnancy test was again positive. She later reported that this pregnancy had also been terminated by elective abortion and that she was in good health.

A second participant in the rabies vaccine group had a positive pregnancy test on study day 90 and gave birth to a healthy male child on study day 289. The parents later reported that the child had died at home at one year of age of an undiagnosed illness that was thought to be consistent with tetanus or meningitis.

#### Serious adverse events

No serious adverse events occurred during the study.

#### Laboratory safety tests

Grade 1 elevated serum creatinine levels were detected in six participants during the study: One female in the rabies vaccine group had Grade 1 creatinine elevations on study days 90, 272 and 364; one male in the rabies group had a Grade 1 elevation on day 7 that persisted through day 363; a female in the malaria vaccine half-dose group and two males in the full-dose group had Grade 1 elevations on study day 0 that either persisted or rose and fell above the upper limit of normal, and remained at Grade 1 on day 364; and one male in the full-dose group had a Grade 1 creatinine elevation only on day 364. These creatinine elevations never increased above Grade 1 and were not associated with clinical abnormalities.

Five participants (two each in the malaria vaccine half-dose and full-dose groups and one in the rabies vaccine group) had Grade 1 elevated ALT levels, and one participant in the malaria vaccine full-dose group had an ALT of 194 U/L on study day 30, prior to immunization. This initial elevation was followed by a rise to 564 U/L on study day 44 and then a decline to 13 U/L by day 60. Extensive investigation including serological tests for hepatitis A, B and C identified no cause for this elevated ALT with the exception of self-administration of a single dose of 150 mg of diclofenac, a non-steroidal anti-inflammatory drug with reported rare liver toxicity.

Hemoglobin levels remained within or slightly above the normal range for all participants throughout the study (11.7 to 17.3 g/dL for males; 10.0 to 14.4 g/dL for females). Grade 1 abnormalities in white blood cell counts were infrequent and balanced by group. Grade 3 low platelet counts were reported for two participants in the malaria vaccine high dose group, but these were unaccompanied by any clinical signs and were determined to be false positive results from an automated cell counter caused by platelet aggregation, based on microscopic examination of the blood.

### Immunogenicity

**Figure 2:**
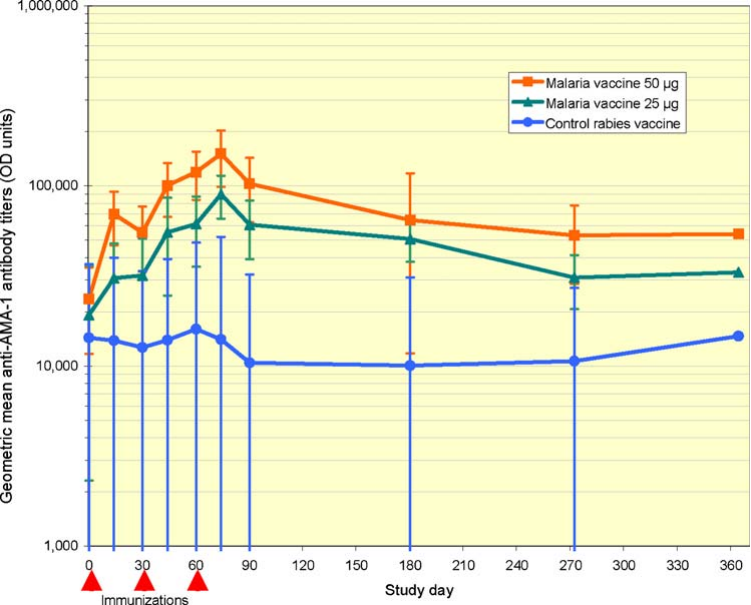
Anti-AMA-1 antibody titers. Geometric mean antibody titers to homologous recombinant AMA-1 for FMP2.1/AS02A full dose, FMP2.1/AS02A half dose and rabies vaccine recipients. Times of each of three immunizations and the start and end of the malaria transmission season are indicated by triangles. Error bars represent 95 percent confidence intervals.

Baseline antibody titers were high in all groups (Figure 2), reflecting a high level of naturally acquired immunity at the end of the malaria transmission season. In contrast to the waning antibody titers seen in the rabies vaccine comparator group following the end of the malaria season, immunization with both the half dose and full dose of the malaria vaccine was followed by significant elevations in anti-AMA-1 antibodies. Antibody titers peaked two weeks after the third immunization (study day 74), with a 4.7-fold rise relative to baseline in the malaria vaccine half-dose group and a 6.4-fold rise in the full-dose group. Mean AMA-1 antibody levels remained higher in the malaria vaccine groups than in the comparator group throughout the study period, although confidence intervals for point-wise comparisons overlapped at study time points after day 90. Mean antibody titers were higher in the full-dose group than in the half-dose group at all time points, although these differences were not statistically significant for point-wise comparisons.

Based on the fitted longitudinal model, participants who received a full dose of the malaria vaccine had a significantly greater mean antibody response at all time points from day 30 (one month after the first immunization) through day 364 (the end of the follow-up period) compared to those receiving the comparator vaccine, and those receiving a half dose of the malaria vaccine had a significantly greater mean antibody response at times points from day 44 through day 272 compared to those receiving the comparator vaccine. A dose-response antibody effect was suggested by higher mean antibody responses in the full-dose malaria vaccine group compared to half-dose group, with these differences approaching but not reaching significance at study days 74, 90, and 180 (*p* = 0.050, 0.053, and 0.079, respectively).

### Ancillary analyses

#### Growth inhibition assays

**Figure 3:**
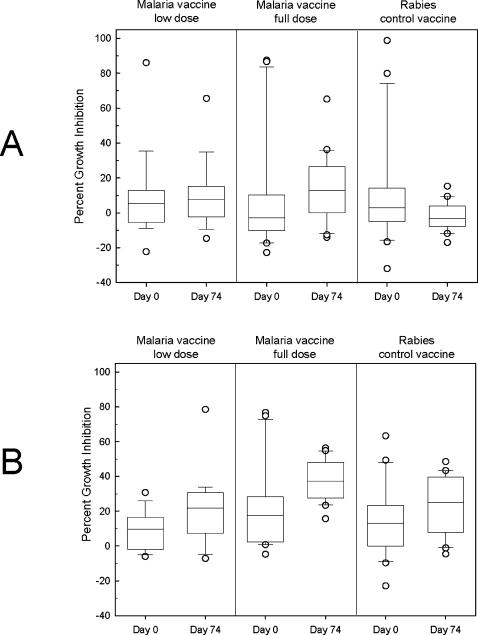
Growth inhibition assays. Mean percent growth inhibition of pre- and post-immunization sera against the 3D7 (A) and FVO (B) clones of *Plasmodium falciparum* grown in vitro. Error bars represent 95 percent confidence intervals.

Measurement of functional anti-AMA1 antibodies in the sera from FMP2.1/AS02A-immunized participants was quantified by determining the levels of parasite lactate dehydrogenase as a biomarker for parasite viability in continuous erythrocyte cultures of synchronized *P. falciparum* parasites. Sera from pre-immunization (day 0) and post immunization (day 74, 2 wk after the third immunization) time-points were tested against both the vaccine-homologous parasite clone, 3D7 (Figure 3A), and against the heterologous clone, FVO (Figure 3B). The immune sera generally gave higher growth inhibition activity against the FVO clone of *P. falciparum* than against the 3D7 clone. There was no difference in mean inhibition of either 3D7 or FVO parasites between day 0 and day 74 in the half-dose group (Wilcoxon Signed Rank Test analyses on paired serum samples; p = 0.490 and 0.156, respectively). Inhibitory activity at day 74 in the malaria vaccine full-dose group was however significantly higher than the day 0 inhibition for this group, against both the 3D7 and FVO clones (*p* = 0.024 and *p* = 0.004, respectively). Sera from participants in the rabies vaccine control group exhibited a significant increase in parasite inhibitory activity against the FVO parasites, p = 0.048, but not for the 3D7 parasites (p = 0.231). Comparison of mean inhibition on day 74 for all groups suggested that while there was no difference in growth inhibition between the rabies vaccine comparator group and malaria vaccine half-dose groups, post-immunization sera from participants who received the full dose of the malaria vaccine had significantly greater growth inhibition activity against both 3D7 and FVO parasites than did post-immunization sera from rabies comparator group (ANOVA *p* = 0.007 and *p* = 0.002, respectively; Tukey post test; *p*<0.05). Compared to baseline, growth inhibition activity against 3D7 tended to decrease following immunization with the rabies vaccine and increase following immunization with either dose of malaria vaccine, although these trends were not statistically significant (Figure 3A).

## Discussion

### Interpretation

This is the first evaluation of the AMA-1-based malaria vaccine FMP2.1/AS02A in a malaria-experienced population. Both the full dose and a half dose of the malaria vaccine had acceptable tolerability. Local reactions were more frequent in both malaria vaccine groups than in the comparator group. Pain and/or swelling at the injection site were experienced by most recipients of the malaria vaccine. Although swelling was often classified as Grade 3 based on the size of the reaction (>50 mm), these episodes of swelling were short-lived and were usually unnoticed by participants. No participants were withdrawn from the study because of adverse events with the exception of one individual who had a transient high elevation of ALT temporally related to immunization but thought likely to be due to ingestion of a drug with known liver toxicity. No serious adverse events were observed. Three pregnancies occurred, one resulting in a healthy male child who died at the age of one year of an undiagnosed illness thought to be consistent with tetanus, and two in one participant that were terminated by elective abortion.

The malaria vaccine elicited high levels of antibodies recognizing the vaccine antigen. Differences in antibody levels between the two malaria vaccine groups, and between malaria vaccine and rabies vaccine comparator groups at time points 4 mo or longer after the last immunization, were not statistically significant when analyzed using point-wise comparisons. However, longitudinal analyses demonstrated that the malaria vaccine full-dose group had significantly higher antibody responses than the rabies vaccine comparator group from 1 mo after the first immunization through the end of the 1 y study period, and the malaria vaccine half-dose group had higher antibody responses than the comparator group from 2 wk after the second immunization through 7 mo after the third immunization. Longitudinal analyses also suggested a dose-related antibody response with a trend toward higher responses in the full-dose group compared to the half-dose malaria vaccine group. Moreover, post-immunization sera from the full-dose group, but not the half-dose group, had significantly greater growth inhibition activity than sera from the comparator group against both homologous and heterologous parasites. Based on these data, the more immunogenic full dose of the vaccine (FMP2.1 50 µg in 0.5 mL AS02A) was selected for further clinical development. This vaccine is presently undergoing evaluation in Phase 1 and 2 clinical trials in children at this site.

### Generalizability

The safety and tolerability profile of the FMP2.1/AS02A vaccine was similar to that seen in a previous trial of this vaccine in North American malaria-naïve volunteers [21] and in trials of a similar recombinant protein blood-stage malaria vaccine with the same adjuvant in this and other African populations [31], [32].

While baseline antibody titers of AMA-1 antibodies were higher in this malaria-experienced population than in malaria-naïve North American volunteers [21], post-immunization titers were of a similar magnitude. Although antibody responses in a recent Phase 1 trial of another AMA-1 vaccine using Alhydrogel as an adjuvant [22] were measured using different methods than those used to measure responses to FMP2.1/AS02A, the FMP2.1/AS02A vaccine induced more uniformly robust responses (with 100% of malaria-naïve and malaria-experienced vaccine recipients showing an increase in antibody titer), possibly due to use of the more potent adjuvant system. In addition to inducing high ELISA activity, the antibodies to FMP2.1/AS02A vaccine induced a moderate level of growth inhibitory activity against parasites expressing both homologous and heterologous AMA-1 [21]. Although a higher degree of background growth inhibition was observed using sera from malaria-experienced adults, immunization with 50 µg of FMP2.1/AS02A induced measurably higher inhibitory antibodies against both homologous and heterologous parasite strains than did the 25 µg FMP2.1/AS02A dose. Interestingly, responses measured against the FVO parasites were significantly higher than baseline at the post third immunization sampling for the rabies controls, suggesting a possible exposure during the study period to parasites similar to FVO with respect to AMA-1 and/or other antigens capable of stimulating allele-specific growth inhibition.

Although these results are promising, until a blood-stage malaria vaccine demonstrates clinical efficacy, immune correlates of vaccine-induced protection and the choice of immunogenicity endpoints for clinical development decisions will remain a matter of reasoned conjecture.

*P. falciparum* AMA-1 is extremely polymorphic, with more than 100 polymorphic amino acid sites, and in vitro experiments and studies in both animals and humans have indicated some degree of allele-specificity in the antibody responses to genetically different forms of AMA-1 [12], [17]–[21]. The FMP2.1/AS02A vaccine is based on AMA-1 sequence from the 3D7 clone of *P. falciparum*. Other AMA-1-based vaccines are being developed based on AMA-1 sequence from the FVO clone [20] and from both 3D7 and FVO [22]. If immunity elicited by an AMA-1 vaccine is allele-specific, then initial vaccine efficacy may depend on the degree of homology at key amino acid residues between the vaccine antigen and AMA-1 in parasites circulating at vaccine trial sites. Moreover, vaccination may result in directional selection favoring AMA-1 alleles that are different from those targeted by the vaccine, resulting in reduced efficacy over time. As this and other AMA-1-based vaccines progress to trials measuring clinical efficacy, it will be important to measure allele-specific efficacy and to identify which specific polymorphisms or sets of polymorphisms are under selection by vaccine-induced immune responses. In the likely event that the genetic diversity of AMA-1 in natural populations of malaria parasites restricts the efficacy of AMA-1 vaccines, it may be necessary to construct a polyvalent and/or chimeric vaccine [33] based on detailed molecular epidemiological and molecular evolutionary analyses of vaccine efficacy and selection in early efficacy trials.

### Overall evidence

Based on its good safety profile, acceptable tolerability, and robust antibody responses, the AMA-1-based malaria vaccine FMP2.1/AS02A is being evaluated in Phase 1 and Phase 2 clinical trials in children aged 1–6 years at the Bandiagara Malaria Project in Mali. If the results of these trials are promising, the development pathway for this vaccine could include incorporating the FMP2.1 antigen as one component of a multi-stage, multi-antigen malaria vaccine in combination with RTS,S [4], improved adjuvant formulations [34] and/or separate development as a disease-blocking vaccine for use in targeted populations in high malaria transmission areas. As AMA-1 malaria vaccines move into efficacy trials, the impact of genetic diversity on malaria vaccine efficacy is likely to emerge as a critical problem requiring integration of methods and concepts drawn from molecular epidemiology, molecular evolution, immunology and structural vaccinology.

## Supporting Information

Checklist S1 CONSORT checklist
(0.06 MB DOC)

Protocol S1 Trial protocol
(0.62 MB PDF)
