# Supplementary material for: Safety and Immunogenicity of an AMA-1 Malaria Vaccine in Malian Adults: Results of a Phase 1 Randomized Controlled Trial
Source: PLoS One. 2008 Jan 23;3(1):e1465. doi: 10.1371/journal.pone.0001465 (PMC2186380; doi:10.1371/journal.pone.0001465)

TITLE OF PROJECT: Double blind randomized, controlled Phase 1 dose escalation trial to evaluate the safety and immunogenicity of the WRAIR AMA1 malaria antigen (FMP2.1) adjuvanted in GSK's AS02A vs. rabies vaccine in malaria-experienced adults in Bandiagara, Mali

Study vaccine FMP2.1/AS02A; Walter Reed Army Institute of Research *Plasmodium falciparum* malaria 3D7 clone based AMA1 antigen adjuvanted with GlaxoSmithKline Biologicals' AS02A

Trial site Bandiagara, Mali, Tel: 223-2-420-495

DMID Protocol No. DMID 04-031

UMB IRB Protocol No. H-25368

HSRRB Study Log No. A-12855

MMVDU Protocol No. Malaria 003

GSK Biologicals Protocol No. 102231 (Malaria-037)

IND No. BB-IND-11140

Version XVII, 17 August, 2004

Title Double blind randomized, controlled Phase I dose escalation trial to evaluate the safety and immunogenicity of the WRAIR AMA1 malaria antigen (FMP2.1) adjuvanted in GSK's AS02A vs. rabies vaccine in malaria-experienced adults in Bandiagara, Mali

IND Sponsor:

Office of the Surgeon General, U.S. Army
The U.S. Army Medical Materiel Development Activity
Contact: H. Marc Howell, Ph.D.
Tel: 301-619-8199
622 Neiman Street
Fort Detrick, Maryland 21702-5009
Tel: 301- 619-2136

Study Co-Sponsor:

Division of Microbiology & Infectious Diseases
NIAID, NIH
Contact: Abdi Naficy M.D., M.P.H.
Medical Officer
Parasitology & International Programs Branch
Division of Microbiology & Infectious Diseases
National Institute of Allergy & Infectious Diseases
National Institutes of Health

6610 Rockledge Drive, MSC 6603
Bethesda, MD 20892, USA
Tel: (301) 435 2866
Fax: (301) 402 0659
E-mail: anaficy@niaid.nih.gov

Principal Investigator: Mahamadou A. Thera M.D., M.P.H.
Malaria Research and Training Center
Department of Epidemiology of Parasitic Diseases
Faculty of Medicine, Pharmacy and Dentistry
University of Bamako
BP 1805, Point G
Bamako, Mali, West Africa
Tel/Fax 223-222-8109
E-mail: mthera@mrtcbko.org

Co-principal Investigator: Christopher V. Plowe M.D. M.P.H.
Center for Vaccine Development
University of Maryland School of Medicine
685 West Baltimore Street, HSF 480
Baltimore, Maryland 21201 USA
Tel 410-706-3082
Fax 410-706-6205
E-mail: cplowe@medicine.umaryland.edu

Clinical Investigators:
MRTC Mali

Ogobara K. Doumbo M.D., Ph.D. (Senior co-investigator)
Dapa A. Diallo M.D.
Drissa Coulibaly M.D.
Abdoulaye Koné M.D.
Ando Guindo M.D.
Karim Traoré, M.D.
Sory Diawara, M.D.
Yacouba Cissoko, M.D.
Malaria Research and Training Center
Department of Epidemiology of Parasitic Diseases
Faculty of Medicine, Pharmacy and Dentistry
University of Bamako
BP 1805, Point G
Bamako, Mali, West Africa
Tel/Fax: + 223-222 81 09; 221 63 61; 222 49 87

CVD Maryland

Kirsten E. Lyke M.D.
Aric Gregson M.D.
Center for Vaccine Development
University of Maryland School of Medicine
685 West Baltimore Street, HSF 480
Baltimore, Maryland 21201 USA

Tel 410-706-5328
Fax 410-706-6205

WRAIR
D. Gray Heppner M.D.
James Cummings, M.D.
Department of Immunology
Walter Reed Army Institute of Research
Tel 301-319-9414
Fax 301-319-7358

MVDB NIH
David Diemert M.D.
Malaria Vaccine Development Branch
National Institute of Allergy and Infectious Diseases
National Institutes of Health
5640 Fishers Lane
Twinbrook I, Room 1125
Rockville, Maryland 20852 USA
Tel 301-443-3915
Fax 301-435-6725

Laboratory Investigators:
MRTC Mali
Mounirou Baby Pharm.D.
Modibo Daou, Pharm.D.
Amagana Dolo, Pharm.D., Ph.D.
Issa Diarra, Pharm.D
Amadou Niangaly PharmD candidate
Malaria Research and Training Center
Department of Epidemiology of Parasitic Diseases
Faculty of Medicine, Pharmacy and Dentistry
University of Bamako
BP 1805, Point G
Bamako, Mali, West Africa
Tel/Fax: + 223-222 81 09; 221 63 61; 222 49 87

WRAIR
David Lanar, Ph.D.
David Haynes, Ph.D.
Sheetij Dutta, Ph.D.
V. Ann Stewart, M.D.
Department of Immunology
Walter Reed Army Institute of Research
Tel 301-319-9414
Fax 301-319-7358

Scientific Investigators:
MVDB NIH
Louis H. Miller M.D. (Consultant)
Malaria Vaccine Development Branch
National Institute of Allergy and Infectious Diseases
National Institutes of Health

	5640 Fishers Lane<br>Twinbrook I, Room 1125<br>Rockville, Maryland 20852 USA<br>Tel 301-443-3915<br>Fax 301-435-6725
USAID	Carter Diggs, M.D., Ph.D.<br>Tel 202-712-5728<br>Lorraine Soisson, Ph.D.<br>Tel 908-369-7704<br>USAID Malaria Vaccine Development Program
GSKBio	Amanda Leach, M.Sc., MRCPCH<br>Clinical Development Manager<br>GSK Biologicals<br>89 Rue de l'Institut<br>1330, Rixensart, Belgium<br>Tel: +32-2-656-7788<br>Fax: +32-2-656-6160<br>Alex Owusu-Ofori, M.D.<br>GSK Biologicals<br>Tel: +32-2-656-6535<br>Fax: +32-2-656-6160
**Local Medical Monitor (Sponsor's Medical Expert)**	Prof. Hamar Alassane Traoré<br>Hospital Point G, BP 1805, Bamako, Mali<br>Tel 223 222 5002
**Clinical Trial Coordinator**	Drissa Coulibaly, M.D.<br>Malaria Research and Training Center<br>Department of Epidemiology of Parasitic Diseases<br>Faculty of Medicine, Pharmacy and Dentistry<br>University of Bamako<br>BP 1805, Point G<br>Bamako, Mali, West Africa<br>Tel/Fax: + 223-222 81 09; 221 63 61
**Data Quality Manager**	Issaka Sagara, M.D., M.Sc.<br>Malaria Research and Training Center<br>Department of Epidemiology of Parasitic Diseases<br>Faculty of Medicine, Pharmacy and Dentistry<br>University of Bamako<br>BP 1805, Point G<br>Bamako, Mali, West Africa<br>Tel/Fax: + 223-222 81 09; 221 63 61; 222 49 87

**Regulatory Affairs Specialist:**	Linda L. Rosendorf, M.S.<br>Center for Vaccine Development<br>University of Maryland<br>685 West Baltimore Ave.<br>Baltimore MD 21201<br>Phone: 410-706-0431<br>Fax: 410-706-4171<br>E-mail: lrosendo@medicine.umaryland.edu
**Study Statistician:**	Alassane Dicko, M.D., M.Sc.<br>Malaria Research and Training Center<br>Department of Epidemiology of Parasitic Diseases<br>Faculty of Medicine, Pharmacy and Dentistry<br>University of Bamako<br>BP 1805, Point G<br>Bamako, Mali, West Africa<br>Tel/Fax: + 223-222 81 09; 221 63 61; 222 49 87<br>E-mail: adicko@mrtcbko.org
**Statistical Consultant:**	William Blackwelder, Ph.D.<br>Center for Vaccine Development<br>University of Maryland<br>685 West Baltimore Street<br>Baltimore MD 21201<br>Phone: 410-706-5328<br>Fax: 410-706-6205<br>E-mail: wcb@boo.net
**IND Coordinator**	Sally Robinson<br>IND Coordinator<br>WRAIR, Dept. Immunology<br>503 Robert Grant Ave<br>Silver Spring, MD 20910<br>Office: (301) 319-9941<br>Fax: (301) 319-7358<br>E-mail: sally.robinson@na.amedd.army.mil
**Clinical laboratory to be used**	Bandiagara Malaria Project Clinical Laboratory,<br>Bandiagara, Mali
**Study Monitors:**	Division of Microbiology and Infectious Diseases, NIH<br>Quality Assurance Office, USAMMDA<br>GlaxoSmithKline Biologicals<br>World Health Organization/Tropical Disease Research

APPROVED AT MALI MALARIA VACCINE DEVELOPMENT UNIT/MALARIA RESEARCH AND TRAINING CENTER BY:

Ogobara Doumbo, M.D., Ph.D.<br>Director of MRTC	____________________<br>Signature	______________<br>Date

I, the undersigned, have reviewed this protocol, including Appendices and I will conduct the clinical study as described and will adhere to the ICH/cGCP and applicable Regulatory requirements. I have read and understood the contents of the Investigator Brochure provided by WRAIR.

Mahamadou A. Thera, M.D., M.P.H.<br>Principal Investigator	____________________<br>Signature	______________<br>Date

Table of Contents

1 LIST OF ABBREVIATIONS 10
2 GLOSSARY OF TERMS 12
3 SUMMARY 13
4 INTRODUCTION AND BACKGROUND 14
4.1 MALARIA PARASITE LIFE CYCLE 14
4.2 DISEASE BURDEN 14
4.2.1 Malaria in Mali 15
4.3 RATIONALE FOR A MALARIA VACCINE 16
4.3.1 The AMA1 antigen 16
4.3.2 FMP2.1/AS02A: the vaccine and its potential clinical development pathway 18
4.3.3 Pre-clinical toxicity, safety and reactogenicity of FMP2.1/AS02A 18
4.3.4 Clinical Experience with FMP2.1/AS02A 19
4.3.5 Antibodies made against FMP2.1 inhibit parasite growth in vitro 21
4.3.6 Preliminary immunogenicity data from AMA1/AS02A Phase 1 trial 22
4.3.7 Clinical Experience with the AS02A Adjuvant 22
4.3.8 Justification of 0, 1, 2 Month Schedule 26
4.3.9 Justification of the vaccine dosing regimen and development plan for vaccine... 26
4.3.10 Comparator Vaccine 26
4.3.11 Safety of RabAvert® rabies vaccine 27
4.3.12 Rationale for double blind controlled design 28
5 OBJECTIVES 28
5.1 PRIMARY OBJECTIVE 28
5.2 SECONDARY OBJECTIVES 28
6 STUDY DESIGN AND METHODOLOGY 29
6.1 OVERVIEW 29
6.2 SITE DESCRIPTION 29
6.2.1 Determination of local clinical laboratory test reference ranges 30
6.2.2 Competencies of staff 30
6.3 INCLUSION CRITERIA 31
6.4 EXCLUSION CRITERIA 32
6.4.1 Justification for the exclusion of children 32
6.4.2 Rationale for using clinical assessment of immunosuppression 33
6.5 TREATMENTS THAT COULD INTERFERE WITH VACCINE-INDUCED IMMUNITY 33
6.6 CONTRAINDICATIONS TO VACCINATION 34
6.7 INDICATIONS FOR DEFERRAL OF IMMUNIZATION 34
6.7.1 Definition of clinical malaria 34
7 CONDUCT OF THE STUDY 35
7.1 GENERAL STUDY ASPECTS 35
7.1.1 Screening and inclusion process 35
7.1.2 Treatment allocation and randomization 36
7.1.3 Vaccination process 36
7.1.4 Post-immunization evaluations 37
7.1.5 Dose escalation 37
7.1.6 Detailed description of study visits 37
7.1.7 Outline of study procedures 42

7.1.8 Health care provision 44
8 SAMPLE HANDLING AND ANALYSIS 44
8.1 OVERVIEW OF COLLECTION TIME POINTS 44
8.2 LABORATORY ASSAYS 45
9 STUDY VACCINES/MEDICATIONS AND ADMINISTRATION 46
9.1 STUDY VACCINES 46
9.1.1 FMP2.1/AS02A vaccine 46
9.1.2 RabAvert® vaccine 47
9.2 DOSAGE AND ADMINISTRATION 47
9.2.1 Formulation of FMP2.1 with AS02A 47
9.2.2 Reconstitution of RabAvert® vaccine 48
9.2.3 Administration of vaccines 48
9.3 VACCINE STORAGE AND ACCOUNTABILITY 48
9.4 METHODS OF BLINDING AND BREAKING THE STUDY BLIND 49
9.4.1 Double blinding 49
9.4.2 Breaking the study blind 49
9.5 CONCOMITANT MEDICATION/TREATMENT 50
10 ADVERSE EVENTS 50
10.1 ELICITING AND DOCUMENTING ADVERSE EVENTS 50
10.1.1 Adverse event definition 50
10.1.2 Surveillance period for adverse events 51
10.1.3 Recording adverse events 51
10.1.4 Solicited adverse events 51
10.1.5 Unsolicited adverse events 52
10.2 ASSESSMENT OF INTENSITY OF NON-SERIOUS ADVERSE EVENTS 52
10.3 ASSESSMENT OF CAUSALITY 53
10.4 FOLLOWING-UP OF ADVERSE EVENTS AND ASSESSMENT OF OUTCOME 54
10.5 SERIOUS ADVERSE EVENTS 54
10.5.1 Definition of a serious adverse event 54
10.5.2 Reporting serious adverse events 55
10.6 PREGNANCY 56
10.7 TREATMENT OF ADVERSE EVENTS 56
11 PARTICIPANT COMPLETION AND DROP-OUT 56
11.1 DEFINITION 56
11.2 PROCEDURE FOR HANDLING DROP-OUTS 56
11.3 REASONS FOR DROP-OUT 57
12 DATA MANAGEMENT AND ANALYSIS 57
12.1 PRIMARY ENDPOINTS 57
12.2 SECONDARY ENDPOINTS 57
12.3 STUDY COHORTS/DATASETS TO BE EVALUATED 57
12.4 ESTIMATED SAMPLE SIZE 58
12.5 PRIMARY ANALYSIS 59
12.5.1 Analysis of demographics 59
12.5.2 Analysis of immunogenicity 59
12.5.3 Analysis of safety 60
13 SITE MONITORING PLAN 60

14 QUALITY CONTROL AND QUALITY ASSURANCE 61
15 ETHICAL CONSIDERATIONS 61
15.1 STATEMENT OF COMPLIANCE AND ETHICAL REVIEWS 61
15.1.1 Institutional Review Boards 61
15.2 INFORMED CONSENT 62
15.2.1 Screening and study informed consent 62
15.2.2 Screening recruitment radio announcement text 64
15.3 ROLE OF LOCAL MEDICAL MONITOR 64
15.4 SAFETY MONITORING COMMITTEE (SMC) 65
15.5 RISKS AND POTENTIAL BENEFITS TO THE PARTICIPANTS 66
15.5.1 Vaccination 66
15.5.2 Medical treatment for participants 66
15.5.3 Rabies vaccination 66
15.5.4 Pregnancy 66
15.5.5 Benefits 66
15.6 PRECAUTIONS TO MINIMIZE RISKS 67
15.6.1 Vaccination 67
15.6.2 Post-vaccination 67
15.6.3 Post-trial information on product safety 67
15.6.4 Protection of study staff 68
15.7 PROCEDURES FOR MAINTAINING CONFIDENTIALITY 68
15.8 COMPENSATION 68
15.9 FINANCING AND INSURANCE 69
16 REFERENCES 69
17 APPENDICES 75
17.1 CONSENT FORMS AND TRANSLATION CERTIFICATION 75
17.2 STUDY COMPREHENSION EXAM 95
17.3 USAMMDA REPORTING SCHEME OF SAES 96

1 LIST OF ABBREVIATIONS

3D7:	A Clone of *Plasmodium falciparum*
AE:	Adverse event
ALT:	Alanine aminotransferase
AMA1:	Apical merozoite antigen-1 of *P. falciparum*
AST:	Aspartate aminotransferase
AS02A:	Adjuvant System 2 of GlaxoSmithKline Biologicals without thimerosal
BMP:	Bandiagara Malaria Project (clinical trial site)
CBER:	Center for Biologics Evaluation and Research
CBC:	Complete blood count
CFR:	Code of Federal Regulations
CMI:	Cell mediated immunity
CRF:	Case Report Form
CS gene:	Circumsporozoite gene of *P. falciparum*
CSP:	Circumsporozoite protein
CTL:	Cytotoxic T lymphocyte
CVD:	Center for Vaccine Development, University of Maryland Baltimore
DMID:	Division of Microbiology & Infectious Diseases, U.S. National Institutes of Health
DNA:	Deoxyribonucleic acid
EGF:	Epidermal Growth Factor
EIR:	Entomologic inoculation rate
FDA:	U.S. Food and Drug Administration
FWA:	Federal-Wide Assurance
ELISA:	Enzyme linked immunosorbent assay
FMP1:	Falciparum Malaria Protein 1 (3D7 MSP1 vaccine candidate antigen)
FMP2.1:	Falciparum Malaria Protein 2.1 (3D7 AMA1 vaccine candidate antigen)
FMPOS:	Faculty of Medicine, Pharmacy and Odonto-stomatology, University of Bamako, Mali
GCP:	Good Clinical Practice
GIA:	Growth Inhibition Assay
GSK Bio:	GlaxoSmithKline Biologicals
GMT:	Geometric Mean Titer
HSRRB:	Human Subjects Research Review Board
ICH:	International Conference on Harmonization
ID:	Identification
IEC:	Institutional Ethical Committee
IM:	Intramuscular
IND:	Investigational New Drug
IRB:	Institutional Review Board (Ethical Review Committee)
MPL:	Monophosphoryl Lipid A
3D-MPL	3-deacylated Monophosphoryl Lipid A
MRTC:	Malaria Research and Training Center
MMVDU:	Mali Malaria Vaccine Development Unit

MSP-1: Merozoite Surface Protein-1 of *P. falciparum*
MSP-2: Merozoite Surface Protein-2 of *P. falciparum*
MVDB: Malaria Vaccine Development Branch, U.S. National Institutes of Health
NIAID: National Institute of Allergy and Infectious Diseases
NIH: U.S. National Institutes of Health
NANP: Repeat epitopes of the circumsporozoite protein
PBMC: Peripheral Blood Mononuclear Cells
PI: Principal Investigator
OCRA: Office of Clinical Research Affairs, DMID, NIAID, NIH
QA: Quality assurance
QC: Quality control
QS21: *Quillaja saponaria* 21 (saponin derivative)
RTS,S: Fusion protein between circumsporozoite protein based antigen and Hepatitis B surface antigen and 226 amino acid polypeptide corresponding to the surface antigen of hepatitis B virus (adw serotype)
SBAS2: SmithKline Beecham Adjuvant System 2
SAE: Serious Adverse Event
SMC: Safety Monitoring Committee
SOP: Standard Operating Procedure
TRAP: Thrombospondin adhesion protein
UMB: University of Maryland Baltimore
USAID: U.S. Agency for International Development
USAMMDA: U.S. Army Medical Materiel Development Activity
WHO: World Health Organization
WRAIR: Walter Reed Army Institute of Research

2 GLOSSARY OF TERMS

Participant(s): Term used throughout the protocol to denote the enrolled individual(s).

Subject(s): Term equivalent to participant in the protocol.

Local Medical Monitor: An individual medically qualified to assure the responsibilities of the sponsors especially as regards the ethics, clinical safety of a study and the assessment of adverse events. Synonymous with Sponsor's Medical Expert.

Study Monitor: An individual who is responsible for assuring proper conduct of a clinical study at one or more investigational sites.

IND Sponsor: The U.S. Office of the Surgeon General, which holds the IND for the vaccine.

Co-Sponsor: The Division of Microbiology of Infectious Diseases, NIAID, NIH, which is supporting the trial financially and providing regulatory and other oversight.

Sponsor: When the term "sponsor" is used throughout the protocol without referring specifically to either the IND sponsor or Study Co-Sponsor, it is referring to both of these.

Eligible: Qualified for enrolment into the study based upon strict adherence to inclusion/exclusion criteria.

Evaluable: Meeting all eligibility criteria, complying with the procedures defined in the protocol, and, therefore, included in analysis (see Section 12.3 for details on criteria for evaluability).

Reactogenicity: refers to the both the expected and unexpected symptoms and signs that are associated with the administration of a vaccine. These include local reactions such as erythema, induration, and tenderness, as well as systemic reactions such as fever, malaise, myalgias, and arthralgias.

Study day: Each participant's study day will commence on the day of their first immunization, which shall be Study Day 0. Thus different participants (including participants in the same cohort who may be immunized on different days) will have different Study Days.

Solicited symptoms: Symptoms that are identified by direct questioning/observation about specific symptoms.

Unsolicited symptoms: Symptoms that are mentioned spontaneously by participants or in response to general, open-ended questions such as "How have you been feeling?"

3 SUMMARY

**Title**	Double blind randomized, controlled Phase I dose escalation trial to evaluate the safety and immunogenicity of the WRAIR AMA1 malaria antigen (FMP2.1) adjuvanted in GSK's AS02A vs. rabies vaccine in malaria-experienced adults in Bandiagara, Mali
**Indication/ Study Population**	HEALTHY MALARIA-EXPERIENCED ADULTS AGED 18-55 YEARS
**Principal Investigator**	MAHAMADOU A THERA, M.D., M.P.H.
**Rationale**	In a small clinical study in malaria-naïve adults FMP2.1/AS02A was acceptably safe and immunogenic. This will be the first study in an endemic area and will evaluate the safety and reactogenicity of 25µg and 50µg dose levels of FMP2.1/AS02A in malaria-experienced adults.
**Objectives**	**Primary**<br>To evaluate the safety and reactogenicity of 25µg and 50µg of WRAIR's AMA1 malaria antigen (FMP2.1) adjuvanted in GlaxoSmithKline Biologicals' AS02A compared to rabies vaccine in malaria-experienced Malian adults.<br>**Secondary**<br>To evaluate the humoral and cellular immune responses and growth inhibition responses to 25µg and 50µg of WRAIR's AMA1 malaria antigen (FMP2.1) adjuvanted in GlaxoSmithKline Biologicals' AS02A compared to rabies vaccine in malaria-experienced Malian adults.
**Study design**	• Double blind, randomized, controlled (RabAvert® Rabies vaccine)<br>• Phase 1 dose escalation<br>• Three study groups: 25µg FMP2.1, 50µg FMP2.1, and rabies vaccine<br>• One study center<br>• Study duration: approximately 12 months per subject<br>• Immunization schedule: Study days 0, 30 and 60<br>• Route: IM in deltoid muscle
**Number of subjects**	• 60 subjects
**Co-primary endpoints**	• Occurrence of solicited symptoms after each vaccination during a 7-day surveillance period (day of vaccination and study days 1, 2, 3 and 7).<br>• Occurrence of unsolicited symptoms after each vaccination during a 30-day surveillance period.<br>• Occurrence of serious adverse events throughout the study period.
**Secondary endpoints**	• Titers of anti-AMA1 antibody at each time point where serology samples are analyzed.<br>• Cellular immune responses to FMP2.1 at baseline and after immunization<br>• Parasite growth inhibition by the in vitro GIA

4 INTRODUCTION AND BACKGROUND

4.1 Malaria parasite life cycle

Among the four species of Plasmodium that cause human malaria, *P. falciparum* is responsible for most disease and death from malaria. Its life cycle is complex. Disease occurs as a result of the asexual blood stage when parasites invade and grow inside red blood cells. *P. falciparum* virulence is partially explained by its ability to use various receptor pathways to invade red blood cells of all ages. Red blood cells infected with *P. falciparum* bind to endothelium allowing the parasite to avoid spleen-dependent killing mechanisms but contributing much to pathogenesis (1).

Anopheline mosquitoes inject sporozoites into the subcutaneous tissue and less frequently directly into the blood stream. The sporozoites then travel to the liver, where they invade hepatocytes. About 6-10 days later, each infected hepatocyte releases 20,000-40,000 merozoites into the bloodstream. Despite the destruction of liver cells, no disease results from the infection during parasite development within hepatocytes. Only the blood stages of the infection produce clinical symptoms and malaria disease.

Red blood cells are invaded through a determined sequence. *P. falciparum* must engage receptors on red cells for binding (2) and undergo apical reorientation, junction formation, and signaling (3;4). The parasite then induces a vacuole derived from the red cell plasma membrane and enters the vacuole by a moving junction. Within this parasitophorous vacuole *P. falciparum* develops over 48 hours producing around 17-32 merozoites, each able to invade other red cells. Particular to *P. falciparum* is its ability to modify the surface of the red blood cell in a way that the infected cells can adhere to the vascular endothelium and other tissues, where they may cause disease. Parasite sequestration in various organs (brain, heart, liver, kidney, placenta) contributes to the pathogenesis of malarial disease.

Following a number of intra-erythrocytic cycles, a small proportion of asexual parasites convert to gametocytes that are critical for the transmission of the infection to others through female Anopheline mosquitoes. Gametocytes cause no disease and there is no known induced natural immune response to this intracellular sexual form of the parasite.

4.2 Disease burden

Malaria is a major threat to the health of two billion people living mainly in sub-Saharan Africa, tropical Asia, Latin America and Oceania. Less developed areas in the world, specifically in Africa, bear the heaviest burden of malaria. The most dangerous parasite species, *P. falciparum*, is responsible for more than one million deaths worldwide each year. More than 90% of these deaths occur among sub-Saharan African children under 5 years old. In area of stable malaria transmission 25% of all-cause mortality in 0-4 year old children has been directly attributed to malaria (5). Evidence from impregnated bed net trials in West Africa indicates that malaria could account directly and indirectly for as much as 60% of all-cause mortality in children aged less than 5 years old (6-8). A testament to its effect in school-age children, up to 50% of medically related school absences are attributable to malaria (9). The overall impact of malaria on human capital development in African children remains unexplored, but it is probably substantial (10). The impact of malaria on the productivity of adults living in endemic areas has

implicated malaria as a major obstacle to development and a contributing cause of poverty. Furthermore, malaria constitutes an increasing hurdle to foreign investment and trade. In areas of intense malaria transmission, the disease generates a complex set of biologic and behavioral responses with long-term effect on economic growth and development (10).

4.2.1 Malaria in Mali

In Mali, malaria is a leading cause of morbidity in the general population and of mortality in children aged less than 10 years. Malaria transmission is seasonal, and varies from very high levels of transmission in the wet holoendemic southern area of Sikasso to malaria epidemic-prone areas at the fringe of the Sahara desert in the dry northern areas of Tombouctou, Gao and Kidal, where malaria transmission is limited to a short rainy season in August.

Bandiagara, (pop. 13,364) located 700 km northeast of Bamako in the heart of the Mopti region on the Dogon plateau, will be the site for this study. Although it is in the Mopti region, it is 75 km from the Mopti flood plain and its malaria epidemiology is quite different, with lower EIRs. Studies conducted from 1999 through 2001 have assessed year-to-year variations in the incidence of malaria disease, stratified by age. Children aged 0-5 years and children aged 6-10 years had similar incidence rates of at least one clinical episode of uncomplicated malaria during the malaria season: 86.2% (n=87) vs. 85.5% (n=69), respectively. No marked yearly variation in the incidence of malaria has been observed from 1999 through 2001. The average number of clinical episodes of malaria per child and per transmission season was 1.92, with a few children experiencing a maximum of four clinical episodes (11).

Adults living in highly malaria endemic regions such as most of sub-Saharan Africa are generally considered to be semi-immune; they have experienced many episodes of malaria and while still susceptible to infection they are protected against malaria disease. Our studies in this area since 1994 show that the annual incidence of malaria infection among persons aged two to 20 years is well over 100% and EIRs in Bandiagara range from 4-40 infected bites/person/month during peak transmission season. Thus any long-term adult resident of Bandiagara town or the Mopti-Bandiagara region can be considered to be “malaria experienced.” It is both important and appropriate to test the safety of a malaria vaccine in a population that has been regularly exposed to malaria because this is the ultimate target population for the vaccine, and because in theory natural infection before or after immunization could either increase or decrease the risks of the vaccine.

In this setting, 63% of the population are Dogon, an ethnic group in which the prevalence of the hemoglobin C (HbC) gene is 5 times higher than the prevalence of hemoglobin S, with heterozygosity of 15-20% (12;13). Case-control studies conducted in this population in 1997-98 showed that carriage of HbC among the Dogon was associated with a protective efficacy of 80% in the reduction of the risk of severe malaria (13).

Census data designed to determine the population at risk showed the incidence of severe malaria among children aged six years or less was 2.5% (n=2284) in 2000. Hyperparasitemia (more than 500,000 asexual parasites/µl) and cerebral malaria were the most common forms of severe malaria, present in 59% and 40% of severe cases, respectively (with multiple diagnoses possible). Severe anemia, defined as hemoglobin < 5g/dl, was present in 18.6% of severe malaria cases (unpublished data).

4.3 Rationale for a malaria vaccine

A safe and effective malaria vaccine in the context of the spread of resistance to antimalarial drugs would be a major contribution to existing control tools. However, developing an effective malaria vaccine has been an elusive goal, and the few malaria vaccine candidates that have progressed to clinical trials have so far shown limited or no efficacy. Two critical steps of the malaria life cycle have been closely examined as potential targets for vaccine-induced immunity: the invasion of liver cells by sporozoites and the invasion of red blood cells by merozoites.

The sporozoite stage of the malaria parasite first attracted the attention of researchers as the most logical target for vaccine-induced immunity. If invasion of the liver by the relatively few sporozoites that are injected by a mosquito could be prevented, the host would have sterile immunity. The identification and cloning of the circumsporozoite protein (CSP), which coats the surface of the sporozoite, was the first major step towards this objective (14). This protein is critical for the invasion of liver cells and contains a peptide sequence that binds to the surface of liver cells (15). The CS gene of *P. falciparum* is composed of a central repeating sequence (NANP) flanked by two non-repeat sequences. The first experimental malaria vaccines tested were based on this repeat sequence; they were poorly immunogenic and only protected a small proportion of the participants immunized (16;17). Although the efficacy was poor, these studies served as proof-of-concept that sterile immunity against malaria can be induced by immunization with a synthetic subunit vaccine.

Subsequent efforts concentrated on the development of formulations with enhanced immunogenicity. Modifications such as the addition of *Pseudomonas aeruginosa* toxin A (18), encapsulation in liposomes containing monophosphoryl lipid A (MPL) (18;19), and inclusion of a mixture of MPL, mycobacterial cell wall skeleton and squalene (20) and the fusion to hepatitis B (21;22) generally resulted in higher antibody levels but, contrary to expectations, did not significantly improve the efficacy. Most recently, GSKBio and the WRAIR developed a recombinant molecule that contains important T helper epitopes from CS as well as the $(NANP)_{19}$ repeat fused to hepatitis B (21). In the initial trials when this molecule was adjuvanted with AS02A (18;23) 6 of 7 participants were protected (24). However, the protection was of short duration (25). Subsequent studies have found the estimated efficacy of the RTS,S/AS02A vaccine to range from 40 to 50% (26). One field trial in The Gambia has also corroborated these results, reporting an efficacy of 34% (95% CI 8.0-53%, p= 0.014) (27).

The merozoite stage of the parasite, like the sporozoite, is a logical target for a malaria vaccine since blockade of erythrocyte invasion would prevent clinical disease (28). Several antigens have been identified that are involved in merozoite invasion of red cells. One of the most studied of these antigens and a promising blood stage vaccine candidate is the merozoite surface protein 1 (MSP1). A recombinant version of the 42 kDa C-terminal portion of MSP1 has been produced at the Walter Reed Army Institute of Research, FMP1 (Falciparum merozoite protein 1), as a histidine-tagged (His6) fusion protein in *E. coli*. The antigen is derived from the 3D7 clone of *P. falciparum* and contains both T-cell and B-cell epitopes. The vaccine is formulated in the same adjuvant system used with the RTS,S antigen. A Phase 1 trial of the FMP1/AS02A vaccine in healthy malaria-exposed Malian adults was initiated in July 2003.

4.3.1 The AMA1 antigen

Another promising antigen for blood-stage vaccine development is the apical membrane antigen-1 (AMA1), a surface protein expressed during the asexual blood stage of *P. falciparum*. AMA1 is produced as an 83 kDa polypeptide by mature schizonts in infected erythrocytes (29), and localizes in the microneme, an apical secretory organelle of the merozoite containing ligands for binding red cell receptors (30). The protein is processed to a 66 kDa protein that is subsequently exported to the merozoite surface at around the time of rupture of the schizont-infected erythrocyte (31). Although its exact function remains undetermined, these observations suggest that it performs a role during merozoite invasion of erythrocytes. Further evidence supporting this comes from studies with monoclonal antibodies to primate and murine plasmodia AMA1 that inhibit *in vitro* parasite invasion of erythrocytes (32;33). This invasion inhibition is not a result of parasite agglutination by antibody, as it can be demonstrated that even the Fab fragments of these monoclonal antibodies block such invasion (34).

P. falciparum AMA1 consists of a signal sequence, a large extracellular domain (ectodomain of 546 amino acids), a transmembrane domain (21 amino acids), and a C-terminal cytoplasmic domain (55 amino acids) (35). Comparisons between all of the known amino acid sequences of AMA1 homologues indicate greater than 50% sequence identity, with 16 cysteine residues conserved in all sequences (36-38). All of the cysteines are found in the ectodomain of the molecule, which is stabilized by eight intramolecular disulfide bonds (39).

AMA1 lacks the sequence repeats observed in other malaria antigens such as the merozoite surface antigens MSP-1 and MSP-2. However, sequence polymorphism resulting from point mutations is observed among alleles of the single copy AMA1 gene in *P. falciparum* (35;37;40). Escalante et al. compared sequences from a total of 44 *P. falciparum* isolates from Kenya, India, Thailand, and Venezuela and observed polymorphism at 118 out of 622 amino acids (41). No insertion/deletion mutations were observed, although approximately 70% of the mutations in the gene encoding AMA1 result in radical substitutions, suggesting positive natural selection. Evidence from this study and previous ones indicate that mutations are predominantly clustered within three regions of the ectodomain that are defined by the eight disulfide bonds and affect both B and T-cell epitopes (37;42).

Seroepidemiologic studies conducted in West Africa and western Kenya demonstrate that natural antibody responses to AMA1 are widespread. Thomas et al. conducted a cross-sectional study of antibody responses to recombinant AMA1 in children ages 2-9 in Guinea-Bissau, an area of moderate endemicity, and in people ages 2-86 in Senegal, an area of holoendemic transmission (43). Overall, a very high prevalence (94%-100%) of naturally acquired serum IgG responses to AMA1, was recorded. Although significant age-dependent changes in the serum antibody levels to this antigen were found among children in Guinea-Bissau, no such correlation was observed among children in Senegal, possibly because high antibody levels develop at an earlier age in areas of more intense malaria transmission.

Another study was performed by Udhayakumar et al. in western Kenya, an area of holoendemic, perennial transmission, to determine the development and maintenance of lymphocyte proliferative and antibody responses to AMA1 epitopes (44). This study showed that proliferative responses and serum antibody responses to B epitopes reached their peak prevalence and magnitude in 5-9-year-olds for most epitopes, an age by which most children have developed clinical immunity to malaria in this area. Serum antibody levels remained stable throughout a single transmission season, unlike T-cell proliferative responses, which were transient and short-lived.

Preliminary studies in Donéguébougou, Mali, demonstrate that natural antibodies exist in a large proportion of individuals (>90%) in this area of intense, seasonal, transmission. In a cross-sectional study of 200 individuals aged 6 months to 45 years conducted in 2002 and 2003, median anti-AMA1 antibody titers reached approximately 1000 at the peak of malaria transmission, although significant variation was seen between different age groups (45).

The naturally acquired antibodies to AMA1 found in people living in malaria-endemic areas have also been shown to inhibit in vitro the growth of *P. falciparum* (46). In this study, human anti-AMA1 IgG was affinity purified from a pool of plasma obtained from Papua New Guinean blood donors who had previously been found to have high titers of antibodies to a variety of *P. falciparum* asexual blood-stage antigens. The affinity-purified human anti-AMA1 antibodies inhibited various strains of *P. falciparum* in a manner similar to that observed with rabbit IgG raised to refolded *P. falciparum* AMA1 3D7, in the *in vitro* merozoite invasion assay. This inhibition was dose-dependent and anti-AMA1 antibodies recognized both strain-specific and conserved epitopes. From this evidence, it is reasonable to postulate that boosting the natural antibody response to AMA1 through vaccination may protect an individual from illness due to the asexual blood stage of *P. falciparum* infection.

The study proposed here is a Phase 1 safety and immunogenicity study of an AMA1-derived vaccine in Malian adults, in an area of seasonal and intense malaria transmission. This will be the first study of the FMP2.1/AS02A candidate vaccine in a malaria-experienced population. If shown to be safe and immunogenic, further studies will be planned with this formulation to assess its safety and efficacy in children, either alone or in combination with other malaria vaccine candidates, possibly including RTS,S, other genotypes of AMA1, or other blood stage antigens such as the MSP1-based vaccine currently being tested in Mali.

4.3.2 FMP2.1/AS02A: the vaccine and its potential clinical development pathway

The WRAIR antigen. FMP2.1 is a lyophilized preparation of the ectodomain of the 3D7 clone of *P. falciparum* AMA1. FMP2.1 is comprised of 478 amino acids, 449 of which are derived from the merozoite surface protein AMA1 of the malaria parasite, *P. falciparum*, 3D7 clone. The protein is produced in and purified from *E. coli* bacteria at the BioProduction Facility of WRAIR. The gene encoding the FMP2.1 protein was chemically synthesized to contain an *E. coli*-optimized codon usage to encode the amino acids representative of the AMA1 protein of the 3D7 clone of *P. falciparum*. The purified antigen will be adjuvanted with AS02A (oil in water emulsion [SB62] + MPL + QS21). This candidate vaccine is intended to limit malaria morbidity and mortality, and possibly infection, by stimulating host immune responses against the apical merozoite antigen-1 of *P. falciparum*. FMP2.1/AS02A is being developed as a potential component of a multi-stage, multi-antigen vaccine compatible with the advanced malaria candidate RTS,S/AS02A and/or blood stage vaccines based on other recombinant AMA1 or MSP1 antigens.

4.3.3 Pre-clinical toxicity, safety and reactogenicity of FMP2.1/AS02A

Clinical grade lots of FMP2.1 protein have been administered to mice, guinea pigs, rabbits and rhesus monkeys. FMP2.1 has been administered to mice and rabbits in combination with AS02A and elicited an excellent immune response without any overt signs of ill health or unusual responses. FMP2.1, administered with or without AS02A, has undergone and passed General Safety Tests in mice and guinea pigs, as well as Pyrogen Tests in rabbits. In primate

studies, detailed clinical, biochemical, and hematological examinations revealed no significant local or systemic reactions in any of the animals. Rhesus monkeys receiving 1/3rd dose levels or full dose levels of the vaccine (FMP2.1/AS02A) developed high titer antibodies to the AMA1 protein with normal hematological and biochemical laboratory tests. In addition, the FMP2.1/AS02A formulation was acceptably tolerated in the rhesus macaque at the highest target human dosage (50 µg) and administration schedule (0, 1, and 2 months) and elicited high antibody titers.

4.3.4 Clinical Experience with FMP2.1/AS02A

BB-IND 11140 entitled "Plasmodium falciparum Recombinant Merozoite Surface Protein (E. coli; AMA1; FMP2.1) Vaccine with AS02A Adjuvant (oil-in-water emulsion +MPL+QS-21; GSK" was filed by USAMMDA with the FDA in July 2003. The first Phase 1 trial was initiated at WRAIR in September 2003 under a protocol entitled "Open-label Phase 1 dose escalation study to evaluate safety, reactogenicity, and immunogenicity of a candidate *Plasmodium falciparum* asexual stage malaria antigen (FMP2.1) administered intramuscularly with GSK Biologicals' adjuvant AS02A to healthy malaria-naïve adults at 0, 1, 2 months (WRAIR 1043)." As of March 2004, 52 volunteers had been screened, and 23 had been enrolled and allocated to one of three dose level groups (1/5 dose [~10 µg of FMP2.1 in 0.5 mL AS02A, 8 volunteers], 1/2 dose [~25 µg of FMP2.1 in 0.5 mL AS02A, 8 volunteers], or full dose [~50 µg of FMP2.1 in 0.5 mL AS02A, 7 volunteers]). Actual versus scheduled immunizations are as follows; low dose 22 of 24, medium dose 22 of 24, and high dose, 19 of 21. There has been one Severe Adverse Event (SAE), an episode of paroxysmal supraventricular tachycardia that occurred two days following immunization, and was determined to be a common, pre-existing condition that was not causally related to immunization.

The predominant adverse events (AEs) have been local pain (25 incidents over 63 vaccinations), local swelling (7 incidents over 63 vaccinations) and headache (6 incidents over 63 vaccinations). Eight grade 3 reactions have been reported in association with immunization (low dose - 0, medium dose - 1, and high dose - 7). Of the grade 3 reactions seen in the high dose group, 5 of 7 occurred in one individual. The grade 3 reactions reported for the individual with 5 of the 7 reported grade 3 reactions were all self-reported, as the individual did not return for follow-up during the time he was symptomatic. These 5 grade 3 reactions reported by this individual included headache, malaise, myalgia, fatigue and joint pain. In addition, this individual reported a fever of 103.2 the evening after vaccination. All of his grade three reactions had resolved or improved to grade 1 within 48 hours after vaccination.

All Adverse Events were expected except the one SAE. Dose escalation was held until relationship of the SAE to vaccination could be established. Evaluation of the volunteer was completed and it was determined the event was not vaccine related. A final SAE report was submitted. No protocol revisions have been made in response to AEs.

Adverse Events by group are summarized below:

Group	A = low dose	B = medium dose	C = high dose
Number of subjects	8	8	7
Total immunizations	22	22	19
Grade 1 reactions	11	20	26
Grade 2 reactions	6	17	21

Grade 3 reactions	0	1	7

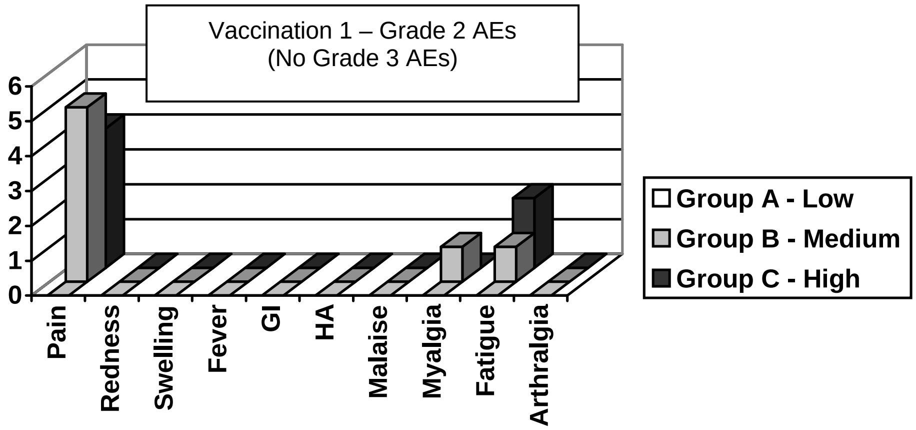

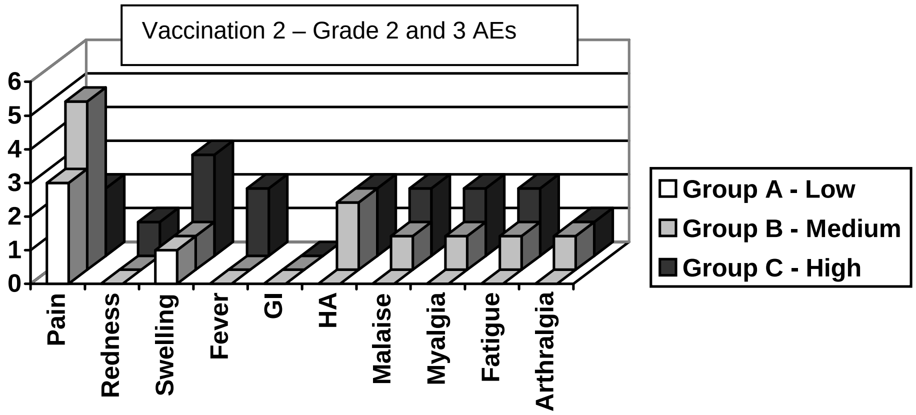

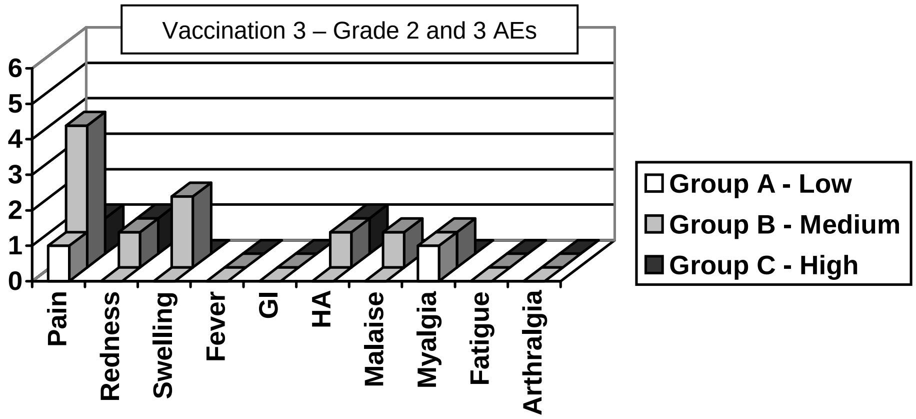

This study used 0.5 mL of AS02A for each dose level of FMP2.1. For reasons of simplicity of reconstitution and administration, it was decided to reduce the volume of AS02A

proportionate to the reduction of FMP2.1 antigen from the full dose in future trials. Thus for the present trial each vial containing 50 µg of FMP2.1 will be reconstituted with 0.5 mL of AS02A from a prefilled syringe as described in Section 9.2.1.

4.3.5 Antibodies made against FMP2.1 inhibit parasite growth in vitro

One method used to evaluate the potential of a vaccine is to determine if antibodies, made against it in animals or humans, can be effective in reducing the parasite's growth in vitro. The Growth Inhibition Assay (GIA) is done by adding immune antisera or purified IgG to a mixture of human red blood cells and parasites and allowing invasion and growth to proceed for an amount of time sufficient for one cycle of growth. The number of infected red blood cells is determined by the addition of a dye, which becomes fluorescent when it binds to the parasite DNA. With the use of a cell sorter 40,000 cells are examined and the percent of infected cells is determined with a high degree of accuracy. Using this method we have examined the antibodies produced following immunization of rabbits with FMP2.1 mixed with either the adjuvant Montanide ISA720 or AS02A. Also, sera from the ongoing Phase 1 trial of FMP2.1/AS02A have been assessed by GIA. The results of all the GIA measurements performed thus far are as follows:

Table 1. Results of Growth or Invasion Inhibition Assay (GIA) for FMP2.1 (recombinant 3D7 AMA1)

			% Inhibition by 20% serum 1-2 weeks after dose #2		% Inhibition by 20% serum 1-2 weeks after dose #3	
	Protein (ug)	Adjuvant (ml, route)	individual values	average	individual values	average
Humans (MAL-033, A)	10	AS02 (0.5 ml, IM)	4%, -3%, 74%, 4%, 17%, 5%, 16%	17%	2%, 2%, NA, 11%, 16%, 17%, 17%	11%
Humans (MAL-033, B)	25	AS02 (0.5 ml, IM)	35%, 4%, 3%, 5%, 14%, -6%, -3%	8%	28%, 91%, 9%, 4%, NA, -1%, -2%	22%
Humans (MAL-033, C)	50	AS02 (0.5 ml, IM)	9%, 3%, -2%, 36%, 31%, 14%	15%	14%, -5%, 7%, 16%, 14%, 91%	23%
Rabbits	50	AS02 (0.5 ml, IM)	NA	NA	50%, 48%, 16%	38%
Rabbits	100	Montanide (1.0 ml, SQ)	NA	NA	87%, 77%, 83%	83%
Rhesus monkeys	50	AS02 (0.5 ml, IM)	48%, 25%, 36%	36%	39%, 18%, 31%	29%

Notes: For GIA, homologous 3D7 P. falciparum parasites were cultured 1 cycle (static) in presence of 20% test serum, new rings determined by flow cytometry and Hoeschst dye 33342, and inhibitions calculated compared with pre-immune or adjuvant controls. All sera was heat inactivated, human sera was also dialyzed to remove any drugs. There is, so far, no direct correlation between GIA and protection from infection or disease. This GIA assay has limitations, for example, it is done in the absence of other immune effector mechanisms such as cellular or cytokine functions.

Clearly, three species of animal, including humans, immunologically responded to FMP2.1 in a fashion that results in the production of antibodies that have the functional capability to inhibit parasite invasion or growth in erythrocytes. This evidence supports the further testing of FMP2.1 in a field setting.

4.3.6 Preliminary immunogenicity data from AMA1/AS02A Phase 1 trial

Figure. Geometric mean anti-AMA1 antibody titer by ELISA (OD). Immunizations were administered on Days 0, 30 and 60. All three dose levels appear to be immunogenic. These results are preliminary as of May 2004 and final analysis will be completed after the trial ends in September 2004.

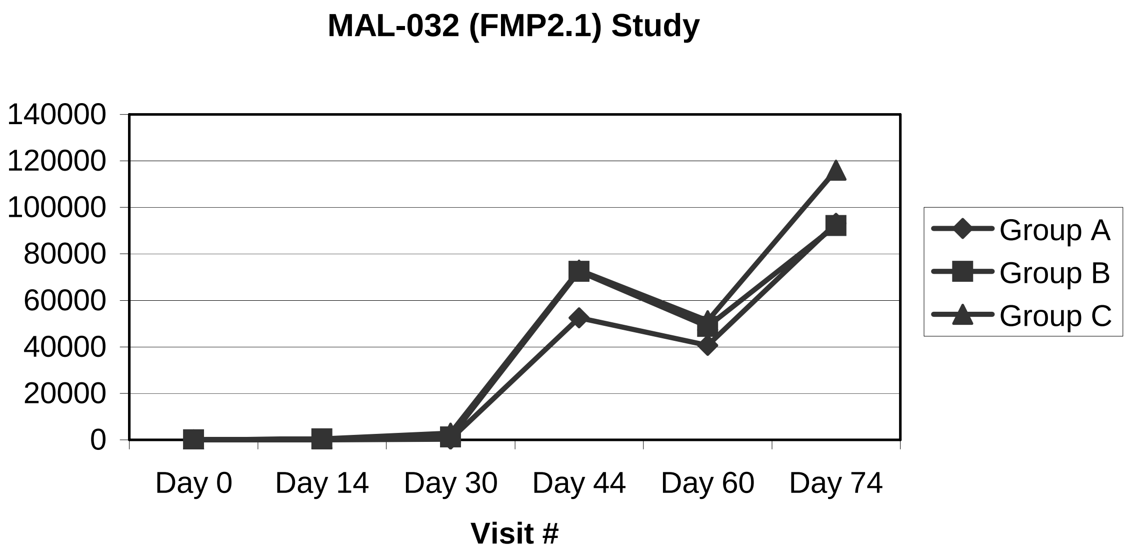

4.3.7 Clinical Experience with the AS02A Adjuvant

The adjuvant system AS02A, previously known as SBAS2, consists of an oil-in-water emulsion combined with two immuno-stimulants, Monophosphoryl Lipid A (MPL) and a saponin derivative known as QS21. QS21 is a highly purified component of a saponin agent derived from the soap bark tree, *Quillaja saponaria* (22;47;48). MPL is a detoxified, deacylated form of monophosphoryl lipid A, derived from the lipopolysaccharide (LPS) of *Salmonella minnesota*. LPS, and more specifically, its lipid A component, has long been known for its strong adjuvant effects; however, until recently, its high toxicity precluded its use in a vaccine formulation. Ribi et al. (19) showed that the monophosphorylated form of lipid A retains its adjuvant function and almost completely loses its endotoxin effects. Subsequently, the 3-deacylated form of MPL was shown to have a further decrease in its toxicity as tested in small animals, but retains its immunopotentiating effect (49). Several immunogenicity studies performed in mice, guinea pigs, monkeys, and humans have shown that inclusion of 3D-MPL into a vaccine preparation potentiates both specific antibody and cellular immune responses (49-51). The term MPL in this protocol refers to the 3-deacylated form of the compound. To date, the bulk of the experience with this formulation in malaria vaccines has been in conjunction with the RTS,S antigen reviewed above.

Vaccine formulations including AS02A and the RTS,S, FMP1 and FMP2.1 antigens have been tested for safety in over 150 malaria-naïve adults in the U.S. with no vaccine-related SAEs and no significant vaccine-related laboratory test abnormalities. For the purposes of this protocol,

detailed information on clinical experience with the AS02A antigen will focus on studies done in adult and pediatric African populations.

In a pediatric Phase 2b trial of FMP1/AS02A in Kenya that is currently in progress, there has been one death, determined by the PI and by Medical Monitor not to be vaccine related based on an autopsy and extensive pathological tissue examinations conducted in the U.S. The cause of death was not definitively determined although pathology reports were consistent with disseminated bacterial infection. Six additional SAEs have been reported, none deemed causally related to the vaccine.

Large clinical experience has been gained in Gambian adults, in a series of Phase 1 and 2b studies in which 711 doses of RTS,S/AS02A were administered. A pooled analysis of the reactogenicity showed that headache (34%) and malaise (25%) were the most frequently reported general symptoms. There were 4 reports of a general symptoms of maximum intensity (grade 3) probably or suspected to be related to vaccination in this population. All were reported in a phase 2b efficacy study after administration of a 4th dose booster vaccination: 1 case of arthralgia, 2 cases of headache and 1 case of malaise (0.1%, 0.3% and 0.1% respectively of documented doses). The majority of solicited local or general symptoms were of short duration (4 days) and all these adverse events reported resolved without sequelae. There was no significant increase in reactogenicity after subsequent (up to 4) doses. In these studies 4 SAEs have been reported. Of these, only one SAE probably related or suspected of being related to vaccination (elevated ALT) occurred during the trial after dose 2. However in the same study, four cases of elevated ALT were reported as SAEs in the rabies vaccine control group. There were 3 other reports judged unlikely to be related to vaccination, these were: 1) erectile impotence, 2) hospitalization with jaundice and urinary tract infection 5 months after study completion (6 months after the last dose); the subject died the next day of suspected fulminant hepatitis, 3) pneumonia cirrhosis and hepatocellular carcinoma reported 5 months after dose 3; the subject died 6 months later. A summary table of the clinical experience with AS02A in adults and children is provided in Table 2.

Table 2. Summary of clinical experience with the AS02A adjuvant in African subjects.

Vaccine	Location	Age(years)	Number of recipients of AS02A	Number of doses	Dose
RTS,S/AS02A	Gambia	18-45	153	435	0.5mL[1]
RTS,S/AS02A	Gambia	6-11<br>6-11<br>6-11	20<br>20<br>20	60<br>59<br>60	0.5mL[1]<br>0.25mL[2]<br>0.1mL[3]
RTS,S/AS02A	Gambia	1-5<br>1-5<br>1-5	30<br>30<br>30	89<br>89<br>89	0.5mL[1]<br>0.25mL[2]<br>0.1mL[3]
RTS,S/AS02A	Mozambique	1-4	30	84	0.25mL[2]
RTS,S/AS02A	Mozambique	1-4	≈1000	N/A	0.25mL[2]
FMP1/AS02A	Kenya	18-55	~20	~60	0.5mL[4]
FMP1/AS02A	Mali	18-55	20	60	0.5mL[4]
FMP1/AS02A	Kenya	1-4	90	N/A	0.5mL[5]<br>0.25mL[6]

1. 0.5mL = 50µl RTS,S + 0.5mL AS02A
2. 0.25mL = 25µl RTS,S + 0.25mL AS02A
3. 0.1mL = 10µl RTS,S + 0.1mL AS02A
4. 0.5mL = 50µl FMP1 + 0.5mL AS02A
5. 0.5mL = 50µl FMP1 + 0.5mL AS02A
6. 0.25mL = 25µl FMP1 + 0.25mL AS02A

N/A – Not available

The experience of the use of the AS02A adjuvant in children to date arises from the clinical development of the RTS,S/AS02A vaccine. Two Phase 1 safety and immunogenicity studies evaluating 0.1 mL, 0.25 mL and 0.5 mL doses of RTS,S/AS02A have been conducted in Gambian children aged 1 to 11 years. A 0.5 mL dose of RTS,S/AS02A contains 50 µg RTS,S, 50 µg MPL®, 50 µg QS21 and 250 µL SB62. Each study was controlled with Imovax® human diploid cell rabies vaccine.

Pain at the injection site was a frequent symptom across all study groups. The incidence of swelling increased with dose level. The majority of general solicited symptoms were mild to moderate in intensity and short lasting. In children aged 6 to 11 years headache was the most frequently occurring general solicited symptom in subjects receiving RTS,S/AS02A (any dose level). In the younger children (aged 1 to 5 years), the most frequently occurring general symptom in subjects receiving the 0.1 mL dose RTS,S/AS02A was fever, reported after 11% of doses compared to 26% of doses in the rabies control group. In subjects receiving 0.25 mL dose RTS,S/AS02A, loss of appetite was the most frequent symptom, being reported after 12% of doses, which compared to 20% of doses in the control group. In subjects receiving 0.5 mL dose RTS,S/AS02A, irritability/fussiness was most frequently reported, occurring after 27% of doses compared to 12% of doses in the control group. Grade 3 general solicited symptoms were infrequent in both studies and resolved or decreased in intensity within 24 hours.

Unsolicited symptoms were recorded with similar (6 to 11 year-olds) or lower frequency (1 to 5 year-olds) in the study vaccine groups compared to the control vaccine groups. The majority of unsolicited symptoms were mild to moderate in intensity and unrelated to vaccination.

Hematocrit values were generally low but comparable between study groups. Six children in the RTS,S/AS02A groups (one in 0.1 mL dose group, five in 0.25 mL dose group) experienced moderate anemia, defined as a hematocrit of 15% to 24% during the period to 30 days post Dose 3. There were no cases of anemia in the 0.5 mL or control groups. All these children had documented malaria episodes before or at the time that anemia was recorded.

All children were monitored for liver function. Among the 6 to 11 year-olds, two children in the RTS,S/AS02A groups experienced a transient rise in ALT levels judged not to be related to vaccination and not clinically relevant. In the younger children, increases in ALT levels from pre-vaccination were observed in two subjects in RTS,S/AS02A groups and two subjects in the rabies groups. Of these, only one case in the rabies control group was judged to be clinically relevant and vaccination was discontinued after Dose 1. No other clinically relevant abnormalities of hematological or biochemical laboratory parameters were observed.

In the interval to 30 days post Dose 3, 6 serious adverse events (SAEs) in total occurred. One SAE occurred in the 6 to 11 year old study; a case of bronchopneumonia was reported in a subject in the rabies control group. The event was not considered causally related to vaccination and the subject made a full recovery. Five SAEs were reported in the 1 to 5 year old study.

Among the RTS,S/AS02A recipients one subject suffered acute malaria with acute upper respiratory tract infection (0.1 mL dose) and one subject suffered cerebral malaria (0.25 mL dose). In the rabies control group three SAEs were reported: acute severe malaria with urinary tract infection and salmonella septicemia, bronchopneumonia with bronchial asthma and accidental death due to drowning. All SAEs were considered not to be related to study vaccines. Apart from the fatal SAE, all subjects made a full recovery and were not withdrawn from the study.

The RTS,S/AS02A (0.25 mL dose) vaccine was evaluated in a double blind, randomized, controlled Phase I study under a new schedule (0, 1, 2 month) in children aged 1 to 4 years in Mozambique. Subjects received RTS,S/AS02A (0.25 mL dose) or Engerix™-B hepatitis B vaccine (GSK Biologicals, Rixensart, Belgium). As previously observed, local reactions at the site of injection were common. Unlike the Gambian pediatric trial, where swelling at the injection site was the most frequently observed local reaction, pain was the most frequently observed reaction. Grade 3 swelling was reported after 23% of doses of RTS,S/AS02A (0.25 mL dose), but did not occur following administration of Engerix™-B. The incidence of swelling in the RTS,S/AS02A group decreased after Dose 2 of vaccine, but increased after Dose 3.

Few solicited general adverse events were reported. In the RTS,S/AS02A group the most frequently reported solicited adverse events related to vaccination were fever (9.5% after all doses) followed by loss of appetite (7.5% after all doses). In the Engerix™-B comparator group, fever (1.2%) was the only solicited general adverse event related to vaccination. There was no trend in increase in solicited general adverse events related to vaccination with subsequent doses. Only one Grade 3 solicited general adverse event was reported in the study; one subject suffered fever in the RTS,S/AS02A group. All solicited general symptoms reported resolved within the 4 day follow-up period after vaccination. The frequency of solicited symptoms was similar to that previously observed in the Gambia trial.

Unsolicited adverse events were recorded over a 30-day surveillance period after vaccination. Unsolicited symptoms were recorded with similar frequency in the study vaccine group compared to the control vaccine group. No unsolicited event was considered by the investigator to be related to vaccination. The unsolicited events reflected the pattern of childhood morbidity expected in the population and unsolicited adverse events were balanced between treatment groups in terms of frequency and severity.

Two children experienced moderate anemia (Hematocrit 15 to 24%) during the period to one month post Dose 3. One child who received RTS,S/AS02A had anemia associated with an acute case of malaria. In the control group the cause was not identified but the child recovered when administered a course of antibiotics and iron supplementation.

In total 7 subjects in the control group and 3 subjects in the RTS,S/AS02A group had an elevation in ALT during the period to one months post Dose 3 (reference range < 60 IU/L). One subject in the control group was observed to have an ALT level of 61 at a single time point. The subject was clinically well and, because the value was just outside the reference range, he was not investigated further. In the other 9 subjects the raised ALT was associated with a viral hepatitis. Five of these subjects had acute hepatitis A. One subject in each group was a chronic carrier of hepatitis B and 1 subject in the RTS,S/AS02A group was suffering from acute hepatitis B. One other subject who received Engerix™-B was HBsAg positive and was a probable chronic carrier of hepatitis B; no confirmatory serology was performed.

No other clinically relevant abnormalities of hematological or biochemical laboratory parameters were observed.

A total of four serious adverse events were reported, two in each group, during the period to one month post Dose 3. One subject in the Engerix™-B group suffered *P. falciparum* malaria; a second subject suffered glomerulonephritis secondary to skin lesions. In the RTS,S/AS02A (0.25 mL dose) group, one subject suffered a febrile convulsion 15 days after the second vaccination and another subject suffered from bronchopneumonia. All SAEs were considered not to be related to study vaccines. All subjects made a full recovery and were not withdrawn from the study.

The FMP1/AS02A trials in Kenya and Mali remain blinded and data on AS02A safety and reactogenicity are not yet available from these trials. However, no vaccine-related SAEs have been reported from these trials as of March 2004.

In summary, these studies, with a large cumulative experience in both malaria-naïve and malaria-experienced participants, suggest that AS02A has a good safety profile.

4.3.8 Justification of 0, 1, 2 Month Schedule

The overall testing program of FMP2.1 aims at carrying out studies in children in subsequent trials. With this goal in mind, the present study uses a 0, 1, and 2 month immunization schedule anticipating that this schedule will be more amenable to eventual incorporation into the Expanded Programme on Immunization of the WHO.

4.3.9 Justification of the vaccine dosing regimen and development plan for vaccine

The clinical development of the candidate malaria vaccine AMA-1/AS02A will progress in four distinct steps; 1) a Phase 1 dose-escalation trial in malaria naïve adults (recently completed), 2) a Phase 1 dose-escalation trial in malaria-experienced adults (the current protocol), 3) a Phase 1 dose-escalation study in young children that will result in dose selection for an efficacy trial, and 4) an efficacy trial of the selected dose in the target pediatric population.

The emphasis of the initial Phase 1 adult studies in malaria naïve and malaria-experienced populations is to describe the safety and tolerability of the highest anticipated vaccine dose intended for pediatric use. Our recent experience for other candidate malaria vaccines containing 10, 25 or 50 µg of antigen formulated in AS02A showed that 25 or 50 µg was more immunogenic than was 10 µg. Therefore, the initial AMA-1 dose for this safety study is 25 µg, rather than 10 µg.

4.3.10 Comparator Vaccine

4.3.10.1 Rationale for rabies comparator vaccine

Having a comparator vaccine is particularly useful in Phase 1 trials conducted in malaria-endemic areas, since background immunity and natural exposure to malaria may make it difficult to interpret immunogenicity data. This is particularly a concern in this trial in a setting with seasonal malaria when transmission intensity will be increasing during the period when vaccine is being administered. Rising titers of antibody to AMA1 could be due to immunization or to natural exposure or both. The use of a control group will permit comparison of immune responses and will result in a clearer interpretation of serological results. While a placebo control group would accomplish this same end, using a vaccine that is beneficial to the subjects increases

the benefit to risk ratio, which is always relatively low in a Phase 1 trial. We have chosen to use rabies vaccine as the comparator for several reasons: 1) the dosing schedule is compatible with that of FMP2.1; 2) to be able to compare results directly with recent and ongoing studies of the FMP1/AS02A vaccine in Mali and Kenya that used rabies vaccine as a comparator; and 3) the available evidence supports a benefit for participants who receive rabies vaccine.

Rabies prevalence in Mali is not known but available data from the Ministry of Health's Division of Epidemiology suggest that the rabies burden is high. Approximately 1,500 dog bites were reported to public health officials in Bamako, the capital of Mali, from 1996 through 1999. The vast majority of the dogs are unvaccinated and in most cases the status of rabies infection is unknown. 124 heads of dogs were examined for evidence of rabies infection. Rabies infection was found in 34 (27%) heads; 7 cases were negative and there were no reported results for 74 cases (60%). These incomplete data allow us to estimate that approximately 30% of dogs that bite humans may be carrying and potentially transmitting rabies infection. In the Bandiagara district health center, approximately one case of human rabies is reported per year. This is likely a considerable underestimate of the true incidence of rabies cases given the general population's reliance on traditional healers and the relatively low utilization of the district health center. Of note, three dog bites were reported among the 40 study subjects during a recent Phase 1 trial of malaria vaccine FMP1/AS02A in Kenya that used the rabies vaccine as a comparator.

We initially planned to use Aventis Imovax rabies vaccine as the comparator but due to a recall and unavailability of this vaccine we will use Chiron RabAvert rabies vaccine which is considered by the CDC to be equally safe and efficacious, and which has been shown to be essentially interchangeable with the Imovax vaccine (i.e. an immunization series started with one vaccine can be successfully completed with the other).

When the RabAvert® rabies vaccine is administered according to the recommended immunization schedule (days 0, 7, 21), nearly 100% of subjects attain a protective titer. In two studies carried out in the US in 101 subjects, protective antibody titers >0.5 IU/mL were obtained by day 28 in all subjects. In studies carried out in Thailand in 22 subjects, and in Croatia in 25 subjects, antibody titers of >0.5 IU/mL were obtained by day 14 (injections on days 0, 7, 21) in all subjects (52-55).

High antibody titers have also been demonstrated with off-label immunization with rabies vaccines. Among participants in England, Germany, France and Belgium who received two immunizations one month apart, nearly 100% of the participants developed specific antibody and the geometric mean titer for the group was 10 IU (56-59). The proposed immunization schedule of 0, 1, and 2 months is therefore expected to be highly successful in conferring protective immunity against rabies among the control participants.

4.3.11 Safety of RabAvert® rabies vaccine

Local and/or mild systemic reactions may occur after injection of RabAvert® rabies vaccine but these are usually transient and do not contraindicate continuing immunization. Local reactions such as induration, swelling and reddening have been reported more often than systemic reactions. In a comparative trial in normal volunteers, Dreesen *et al.* (52;60) described their experience with RabAvert compared to a HDCV rabies vaccine. Nineteen subjects received RabAvert and 20 received HDCV. The most commonly reported adverse reaction was pain at the injection site, reported in 45% of the HDCV group, and 34% of the RabAvert group. Localized lymphadenopathy was reported in about 15% of each group. The most common systemic reactions were malaise (15 % RabAvert group vs. 25 % HDCV group), headache (10 %

RabAvert group vs. 20 % HDCV group), and dizziness (15 % RabAvert group vs. 10 % HDCV group). In a recent study in the USA (4), 83 subjects received RabAvert and 82 received HDCV. Again, the most common adverse reaction was pain at the injection site in 80% in the HDCV group and 84% in the RabAvert group. The most common systemic reactions were headache (52% RabAvert group vs. 45% HDCV group), myalgia (53% RabAvert group vs. 38% HDCV group) and malaise (20% RabAvert group vs. 17% HDCV group).

None of the adverse events was serious, almost all adverse events were of mild or moderate intensity. Statistically significant differences between vaccination groups were not found. Both vaccines were generally well tolerated. Uncommonly observed adverse events include temperatures above 38°C (100°F), swollen lymph nodes, and gastrointestinal complaints. In rare cases, patients have experienced severe headache, fatigue, circulatory reactions, sweating, chills, monoarthritis and allergic reactions; transient paresthesias and one case of suspected urticaria pigmentosa have also been reported. Human serum albumin (HSA) is present in RabAvert at concentrations less than 0.3 mg/dose. No type III hypersensitivity reactions have been observed with RabAvert. Serious systemic anaphylactic reactions or neuroparalytic events have been reported in association with RabAvert administration. Against a background of 11.8 million doses distributed world-wide 10 cases of encephalitis (1 death) or meningitis, 7 cases of transient paralysis including 2 cases of Guillain-Barré Syndrome, 1 case of myelitis, 1 case of retrobulbar neuritis, and 2 cases of suspected multiple sclerosis have been temporally associated with the use of RabAvert. Also, 2 cases of anaphylactic shock have been reported.

4.3.12 Rationale for double blind controlled design

A double-blind controlled dose escalation trial will allow assessment of vaccine safety in each of three groups, one group each to receive medium and full dose levels of the experimental vaccine, and one group to receive the comparator vaccine. Thirty adults will be randomized to receive the medium dose level of FMP2.1 (n=20) or rabies vaccine (n=10) and thirty to receive the full dose level of FMP2.1 (n=20) or rabies vaccine (n=10). The division of the rabies group into two groups of ten is done to maintain blinding at each immunization time point, and all participants who receive the rabies vaccine will be analyzed as a single group. The sample size of the groups, however, will not allow detection of anything other than very large differences in the occurrence of adverse events among the three groups. The advantage of double blinding is to remove the potential for investigator and participant prejudgment about the effects of the vaccines in the reporting of adverse events.

5 OBJECTIVES

5.1 Primary objective

- To evaluate the safety and reactogenicity of two dose levels of WRAIR's AMA1 malaria antigen (FMP2.1) adjuvanted in GlaxoSmithKline Biologicals' AS02A compared to rabies vaccine in malaria-experienced Malian adults aged 18-55 years inclusive.

5.2 Secondary objectives

1. To measure the magnitude and duration of antibody response to FMP2.1

2. To measure cellular immune responses to FMP2.1 at baseline and after immunization
3. To measure the inhibition of parasite growth by the in vitro GIA
4. To determine the specificity of the antibodies to diverse AMA1 genotypes in addition to 3D7, by measuring by ELISA, and GIA on parasites with typed AMA1.

6 STUDY DESIGN AND METHODOLOGY

6.1 Overview

- Double blind, randomized controlled dose escalation phase I study
- One study center
- Screening will be done within 35 days prior to the first immunization
- Dose escalating by approximately 25µg and 50µg of FMP2.1 adjuvanted with 0.25 mL and 0.5 mL of AS02A, respectively
- At each of the two FMP2.1 dose levels, 30 adults will be randomized to receive either FMP2.1/AS02A (n=20) or RabAvert® vaccine (n=10)
- Immunization schedule will be on study days 0, 30 +/- 7, and 60 +/- 7
- Each consecutive immunization with the 50 µg dose of FMP2.1 will be administered at least 14 days after the corresponding immunization with the 25 µg dose of FMP2.1 to permit review of day 7 safety laboratory results
- Route of immunization will be the deltoid muscle IM
- CRFs will serve as source documents. Only information that cannot be collected initially onto CRFs (namely, clinical laboratory test results and adverse event medical records) will first be collected onto separate source documents prior to transcription into CRFs.
- Study duration will be approximately 12 months per participant
- 7-day surveillance (day of vaccination and study days 1, 2, 3 and 7 for solicited adverse events
- 30-day surveillance for unsolicited adverse events
- Follow-up of serious adverse events (SAEs) until resolution
- Beginning Study Day 120, participants will be visited and assessed by local guides at home at monthly intervals and will be asked to return to clinic every 3 months for safety surveillance.
- The primary analysis will be conducted for all primary and secondary endpoints at a data-lock-point one month post Immunization 3 (study day 90), after which the Primary Study Report will be produced.
- The study will then continue in a single-blind fashion. Data gathered during this period will be reported in an addendum report.
- At the end of the study participants will be informed which vaccine they received and those who received malaria vaccine will be offered immunization with the rabies vaccine.

6.2 Site description

The study will be conducted in Bandiagara, which has been the site of MRTC malaria clinical, epidemiological and entomological studies since 1993. Since 1998 Bandiagara has been the site of an NIH-supported contract for developing a site for testing malaria vaccines, known as

the Bandiagara Malaria Project (BMP). The BMP has completed full censuses of Bandiagara, established a laboratory with the capability of preserving sera, PBMCs, live parasites and DNA; and a clinical research center where malaria diagnosis and hemoglobin and blood glucose levels are routinely determined and where children with severe malaria are hospitalized and cared for. The BMP conducted a case control study that enrolled 253 cases of severe malaria matched to 253 each of uncomplicated malaria and healthy controls. A cohort study of 427 subjects aged 3 months to 20 years with nested drug resistance studies was conducted from 1999 through 2001 as well as a matched cohort incidence trial of 338 pairs or 676 children from 2002-2004 to monitor the effects of concomitant schistosomiasis on malaria acquisition. The rate of loss to follow-up has consistently been less than 7%. The BMP clinical facilities are located within the Bandiagara District health center, where a two large blocks of rooms were renovated in 2003 for a Phase 1 malaria vaccine trial, “Double blind randomized controlled Phase 1 trial to evaluate the safety and immunogenicity of WRAIR’s MSP1 candidate malaria vaccine (FMP1) adjuvanted in GlaxoSmithKline Biologicals’ AS02A vs. Rabies vaccine in semi-immune adults in Bandiagara, Mali” The clinical research facility includes an air-conditioned clinical laboratory and vaccine storage preparation room, six private consultation rooms, a procedure room, covered waiting area, two vaccination rooms, a resuscitation suite, an observation room, and storage and administrative space. Directly across the road is the Bandiagara Center for Research on Traditional Medicine, where two rooms are exclusively used for BMP laboratory activities. Locked cabinets with restricted access are used for data storage.

The BMP team has been trained to conduct GCP-compliant studies, using source documents, written informed consent and standardized case report forms. The BMP team has also established a strong trust and rapport with the community, and the community is very accepting of conducting malaria vaccine trials there.

The site is being connected to the MRTC central laboratory in Bamako via a VSAT system, which will allow a high-speed communication link with Bamako, U.S. partner institutions and the Internet.

6.2.1 Determination of local clinical laboratory test reference ranges

In most African countries, reference ranges for clinical laboratory tests are those used in clinical laboratories from Europe and the United States. Laboratory tests such as hemoglobin concentration, white blood cell count, platelet count, serum creatinine and liver enzymes concentration, which are used to determine malaria vaccine potential toxicity, have a different distributions in the West African populations than in European and American populations (61). A survey was therefore undertaken in Bandiagara in March 2003, to determine local reference intervals for clinical laboratory values. This survey followed the methods described in the National Committee for Clinical Laboratory approved guidelines (NCCLS) (62). 120 each healthy male and female residing in Bandiagara were included and reference limits were calculated nonparametrically by taking the 2.5 and 97.5 percentiles of the observed sample values. The 95% confidence intervals of the reference limits were also calculated. The reference intervals calculated were used to establish the upper limits of normal ranges to determine locally appropriate exclusion criteria and toxicity ranges for adverse events for this trial.

6.2.2 Competencies of staff

The BMP clinic team is composed of medical doctors and doctors of pharmacy who are experienced in conducting complex studies with long periods of follow-up and complying with ICH/GCP requirements. Malian and U.S. investigators on this protocol have worked in close collaboration at this field site since 1997. Past studies have been implemented with the aim of preparing the team to conduct GCP-compliant malaria vaccine trials, culminating in a Phase 1 trial of a blood stage malaria vaccine conducted in 2003 through 2004. The team's training program in recent years has included GCP workshops in Mali, in Baltimore at the University of Maryland's Center for Vaccine Development, and at the Johns Hopkins Summer Institute on Biostatistics and Epidemiology. Several of the study investigators have attended courses and workshops on bioethics. Each year since 2002 MRTC investigators have attended a GCP workshop directed by the PI on this protocol and other senior MRTC investigators, and specific training on each clinical trial protocol and its study procedures are conducted. A specific training program on protocol-specific study procedures will be designed for the local guides and supporting staff in Bandiagara. The PI, co-investigators and the BMP clinic team will carry out all the study procedures. The local guides have many years of experience with the conduct of clinical research, and the senior guide is also trained as a nurse. Local guides will be provided with study-specific training, and are fluent in French as well as many local languages and dialects. They will have the responsibility to maintain contact with study participants, to remind them of their scheduled clinic visits, to guide them through the clinical research center from post to post (e.g. from vaccination room to observation room) and to document any travel outside the area of Bandiagara.

The U.S.-based clinical investigators have extensive experience with domestic and foreign vaccine trials as well as other clinical trials and field research in Africa and many other tropical regions. The U.S. co-PI was present for each immunization of the 2003 FMP1/AS02A Phase 1 trial in Mali, each time accompanied by one or more experienced clinical investigators from the CVD, WRAIR, and/or GSKBio. A similar level of close collaboration between Malian, U.S. and European investigators will be employed for this trial.

The Biostatistics and Epidemiology Unit of the MRTC is directed by senior investigators who hold graduate degrees in epidemiology and biostatistics from U.S. universities and staffed by five data entry technicians. The Unit has implemented data management systems based on 21 CFR 11 guidelines, and this will be their third malaria vaccine trial conducted under IND regulatory standards. The CVD statistician who will provide back-up and oversight of the data management and analysis process has decades of experience as the biostatistician for domestic and foreign Phase 1, 2, 3 and 4 vaccine trials.

6.3 Inclusion criteria

- A male or non-pregnant female aged 18-55 years inclusive at the time of screening.
- For women, willingness not to become pregnant until 1 month after the last immunization (pre-menopausal female participants will be referred to the local family planning clinic in Bandiagara, which offers several means of contraception that are approved and recommended by the Malian Ministry of Health)
- Separate written informed consent obtained from the participant before screening and study start, respectively
- Available and willing to participate in follow-up for the duration of study (12 months)

6.4 Exclusion criteria

The following criteria will be checked at the time of study entry (i.e. following screening, at the time that participants are enrolled into the vaccine trial itself). If any apply at the time of study entry, the subject will not be included in the study:

- Previous vaccination with any investigational vaccine or with any rabies vaccine
- Use of any investigational or non-registered drug or vaccine other than the study vaccine(s) within 30 days preceding the first study immunization, or planned use up to 30 days after the third immunization
- Chronic administration (defined as more than 14 days) of immuno-suppressants or other immune-modifying drugs within six months prior to the first immunization. This will include any dose level of oral steroids or inhaled steroids, but not topical steroids
- Planned administration/administration of a vaccine not foreseen by the study protocol within 30 days before the first study immunization with the exception of tetanus toxoid
- Any confirmed or suspected immunosuppressive or immunodeficient condition, including human immunodeficiency virus (HIV) infection
- Any confirmed or suspected autoimmune disease
- History of allergic reactions or anaphylaxis to immunizations or to any vaccine component
- History of serious allergic reactions to any substance, requiring hospitalization or emergent medical care
- History of allergy to tetracycline, doxycycline, nickel, Imidazole, chicken eggs, processed bovine gelatin, chicken protein, neomycin, or amphotericin B.
- History of splenectomy
- Serum ALT ≥43 IU/L
- Serum creatinine level >113 μmol /L for males and 70 μmol /L for females
- Hb <11 g/dL for males and <10 g/dL for females
- WBC <4.0 x $10^3/mm^3$ or >13 x $10^3/mm^3$
- Absolute lymphocyte count ≤1.4 x 10^3 /μl
- Thrombocytopenia < 108,000/μl
- More than trace protein, more than trace hemoglobin or positive glucose in urine
- Administration of immunoglobulins and/or any blood products within the three months preceding the first study immunization or planned administration during the study period.
- Suspected or known current alcohol or illicit drug abuse
- Pregnancy or positive urine beta-HCG on the day of or prior to immunization
- Breastfeeding
- Simultaneous participation in any other interventional clinical trial
- Acute or chronic pulmonary, cardiovascular, hepatic, renal or neurological condition, or any other findings that in the opinion of the PI may increase the risk of participating in the study
- Other condition that in the opinion of the PI would jeopardize the safety or rights of a participant in the trial or would render the participant unable to comply with the protocol

6.4.1 Justification for the exclusion of children

The FMP2.1/AS02A vaccine has been tested in 23 adults in the United States, and this will be its first use in a malaria-exposed population. Natural malaria transmission may affect

both the safety and immunogenicity of the vaccine, therefore it is felt that it is more ethical to perform this Phase 1 study first in adults who can give full, informed independent consent. Following this study, assuming the vaccine is shown to be safe and immunogenic in this study population, further studies in children are anticipated.

6.4.2 Rationale for using clinical assessment of immunosuppression

We do not plan to test for HIV at the time of screening. HIV seroprevalence is 1.7% in Mali, one of the lowest rates in sub-Saharan Africa. Although no serosurveys have been done in Bandiagara itself, this site is in a remote rural area and almost certainly has a lower prevalence rate than the average for the entire country. After working at this site for the past seven years, we have only very rarely encountered persons with illnesses that raised clinical suspicion of an immunosuppressive disease. Therefore, the training of staff and establishment of programs that would be necessary for voluntary counseling and testing for HIV would likely yield few, if any, cases of HIV in this small study.

Second, this study has been designed to produce comparable results to other Phase 1 trials of recombinant protein blood stage malaria vaccines adjuvanted with AS02A, including trials of FMP1/AS02A conducted in Kenya and Mali. In these studies, a similar approach was taken to exclude persons with clinical evidence of immunosuppressive disease but not test for asymptomatic HIV infection, based on the rationale that it is necessary to assess the safety and immunogenicity of this vaccine in generally healthy adults who are representative of the population from which they are drawn. In Kenya, where rates of HIV infection are higher, this general population includes many persons living with HIV and a study that excluded them would be of less value. Eventually, it will be necessary to demonstrate the safety and immunogenicity in persons living with HIV for any malaria vaccine to be employed in Africa, but because of the low rates of HIV infection in Mali these studies will have to be conducted elsewhere, and meaningful subanalyses of HIV-infected and uninfected vaccinees will require the larger sample sizes of Phase 2b or Phase 3 trials.

6.5 Treatments that could interfere with vaccine-induced immunity

The following criteria will be checked at each visit. If any become applicable during the study, the subject will not be required to discontinue the study, but a separate immunogenicity analysis may be done that excludes these individuals. See section 12.3 for definition of study cohorts/datasets to be evaluated.

- Use of any investigational drug or vaccine other than the study vaccine(s) during the study period.
- Chronic administration (defined as more than 14 days) of any dose level of immunosuppressants or other immune-modifying drugs during the study period and chronic daily use of inhaled steroids. Intermittent use of inhaled and topical steroids are allowed.
- Administration of a vaccine not foreseen by the study protocol during the period starting from 30 days before the first study immunization and ending 30 days after the third immunization.
- Administration of immunoglobulins and/or any blood products up to 30 days after the last study immunization.

6.6 Contraindications to vaccination

The following criteria will be checked prior to each immunization and are contraindications to further immunization. However, the study participants will be encouraged to continue to participate in the surveillance schedule for safety evaluation.

- Systemic hypersensitivity reaction following administration of the study vaccine. Severe (i.e., Grade III) local reactions will be evaluated as outlined in Sections 15.3 and 15.4 to determine whether or not further study immunizations should be administered
- Positive urine β-HCG

6.7 Indications for deferral of immunization

The following adverse events constitute grounds for temporary deferral of vaccine administration; if any one of these adverse events occurs at the time scheduled for vaccination, the subject may be vaccinated at a later date, within the time interval specified in the protocol section 7.1.7, or withdrawn at the discretion of the investigators. The subject will be followed until resolution of the event, as with any adverse event (see section 10). A subject who is withdrawn from the study, will be encouraged to remain in the safety evaluation for the duration of the study.

- Oral temperature >37.5^{o}C or evidence of clinical malaria (see section 6.7.1) at the time of vaccination will warrant deferral of immunization until fever and symptoms resolve
- Any other condition that in the opinion of the investigator poses a threat to the individual if immunized or that may complicate interpretation of the safety of the vaccine following immunization.

Such individuals will be followed daily in the clinic until the symptoms resolve or the window for immunization expires. No further vaccination will be performed if the subject does not recover (oral temperature ≤37.5^{o}C and/or lack of symptoms) within 7 days of the originally scheduled vaccination date. The subject, however, will be followed for safety and immunogenicity.

If the individual meets any of the above criteria for deferral on the day of first immunization the PI may elect to exclude the subject from further participation in the study.

6.7.1 Definition of clinical malaria

A clinical episode of *P. falciparum* malaria is defined as the presence of *P. falciparum* asexual parasitemia on Giemsa-stained thick blood smear films in the presence of the following: (i) fever defined as oral temperature $\geq$ 37.5^{o}C in the absence of other evident clinical conditions that could explain the fever; and/or (ii) one or more of the following symptoms consistent with malaria including but not limited to headache, vomiting, abdominal pain, or myalgia with or without fever. This case definition is based on seven continuous years of clinical experience treating children and adults with malaria in Bandiagara, and represents the current standard for clinical decisions to treat malaria in this setting. Study investigators will use their best clinical judgment in deciding when to treat malaria and will not be precluded from treating malaria by this definition.

7 CONDUCT OF THE STUDY

Detailed SOPs will be used for the study procedures described in this section. Staff and investigators will be trained in the SOPs relevant to their duties and sign copies of the SOPs to document this training. Copies of SOPs will be available for inspection and review by the DMID, USAMMDA, GSKBio, WRAIR and study monitors. The general methods that will be used are summarized in the following sections.

7.1 General study aspects

7.1.1 Screening and inclusion process

Recruitment will be progressive until 60 adults of either gender who fulfill the inclusion criteria are included in the study. Volunteers will be recruited by non-coercive methods among adults 18-55 years of age residing in Bandiagara. They will be recruited after coming voluntarily to the BMP clinic. No undue influence will be exerted upon volunteers to obtain their consent. After the study has been explained to the potential volunteers, they will be allowed to leave and return later with their decision; this will allow time for them to discuss the study with their family and carefully consider their involvement in the study. Finally, at the BMP clinic, the individual consent process will be conducted in a separate and private room to ensure confidentiality, to reduce the likelihood of other participants influencing the decision to participate, and to allow further time to make a final decision.

After community information is disseminated as described in Section 15.2.1, all interested and potentially eligible adults aged 18-55 will be invited to visit the study clinic on a specific date. These individuals will receive oral and written explanation of the study, after which screening consent will be obtained from those willing to participate. All screening tests, medical history and examinations will be performed only after screening consent is obtained. Women who express unwillingness to avoid pregnancy will be excluded from the remainder of the screening process. Any clinically relevant finding that is discovered upon screening will either be treated appropriately by study clinicians or referred for more comprehensive diagnosis and treatment at the government health clinic. Study clinicians will generally handle acute, simple conditions such as malaria or other acute infections. More complicated or chronic conditions, such as chronic renal or heart disease, will be referred to appropriate sources of medical care.

Upon screening, the Investigator will prepare a screening form for each participant. This screening form will later become part of the CRF for participants enrolled in the vaccine trial. A unique screening identification number will be assigned to each study participant. Participants will provide a medical history, with special attention to any history of recurrent infections to suggest immune suppression, previous history of splenectomy and prior vaccine reactions. They will also undergo physical examination and laboratory screening tests, which include (see section 8.2 for details on laboratory testing to be performed): complete blood count (CBC) creatinine, ALT, urine dipstick analysis, and urine β-HCG (for females to exclude pregnancy). Urine β-HCG will be obtained on female participants just before each immunization. A participant who meets any of the exclusion criteria will be excluded. Participants excluded from this study because of significant abnormalities will be managed initially by study clinicians and referred to the local health center for evaluation as necessary. All screening tests will be completed within the 35 days prior to entry into the study. Laboratory studies may be conducted at other times

during the course of the trial if the investigators judge it necessary for the safety of the participant. All screening and follow-up diagnostic laboratory testing will be performed at the Bandiagara Malaria Project laboratory in Bandiagara and if applicable at the MRTC clinical laboratory in Bamako. Recruitment will continue until 60 eligible participants have fulfilled all of the inclusion criteria and none of the exclusion criteria, and signed the study consent form. Should participants change their mind and decline to participate prior to immunization, additional participants will be screened until 60 participants are enrolled. Each study subject will receive a photo ID card and a copy of the photo ID will be attached to the CRF folder for each participant.

7.1.2 Treatment allocation and randomization

Individual participants will be randomized to receive either full dose or half dose FMP2.1 or RabAvert® Rabies vaccine without stratification for gender. Participants will be randomized to two groups of 30 subjects (cohorts 1 and 2). The first 30 eligible volunteers will constitute the first cohort and will be randomized in a 2:1 ratio to receive 25μg of FMP2.1 adjuvanted with 0.25 mL of AS02A (n=20) or RabAvert® vaccine (n=10). Similarly, the next 30 eligible volunteers will constitute the second cohort (cohort 2) and will be randomized in a 2:1 ratio to receive 50μg of FMP2.1 adjuvanted with 0.50 mL of AS02A (n=20) or RabAvert® vaccine (n=10). Within each cohort, treatments will be assigned to subject ID numbers in randomized blocks. Randomization to either the two vaccines (FMP2.1/AS02A or RabAvert®) will be done using a computer-generated randomization list. The randomization list will contain sequential codes linking a study number to a vaccine assignment (FMP2.1/AS02A or RabAvert®). Study numbers will be assigned to participants of each cohort in the order in which they provide written informed consent to be enrolled in the vaccine trial. The vaccine and dose that are assigned during the first immunization will be maintained for second and third immunizations. Access to the randomization list will be exclusively limited to the study drug manager(s)/pharmacist(s), study statistician in Bamako, and statistical consultant in Baltimore. These individuals are unblinded and will not be involved in study participants' further evaluation. The Local Medical Monitor will also keep one set of the randomization code in a sealed envelope, in the event that emergency unblinding is required. The reason for any unblinding will be documented as well as the steps taken.

7.1.3 Vaccination process

Before each vaccination, criteria for continued eligibility will be reviewed and verified. The volunteer participation will be pursued only if she or he is deemed eligible to receive vaccination. A history-directed physical examination will be done and oral temperature, blood pressure, pulse and baseline general symptoms will be recorded. Venous blood will be collected for laboratory analysis and urine will be collected from female volunteers and tested for β-hCG by dipstick testing.

After the participant's identity is checked by comparing his/her photo ID and study number with that on the CRF, he/she will be vaccinated by intramuscular injection into the left deltoid muscle. If any local impairment prevents administration of the vaccine into the upper arm for that particular immunization, the vaccine may be administered into the opposite arm in the

deltoid region (see section 9.2.3). Vaccination will be done on study days 0, 30 +/- 7 and 60 +/- 7. Double blinding will be maintained as described in detail in Section 9.4.

7.1.4 Post-immunization evaluations

After immunization, the participant will be observed for onset of local and systemic reactions. The participant will be observed for a minimum of 30 minutes post-vaccination. Signs and symptoms (as detailed in Section 10) will be solicited and recorded by the investigators according to adverse events recording procedures (see Section 10).

Participants will complete a 7-day surveillance after each vaccination at the BMP clinic center (including day 0, the day of vaccination). All adverse events occurring during this 7-day period will be followed until resolution or stabilization. If any symptom persists beyond the 7-day surveillance period the participant will be followed daily until resolution of the adverse event.

After the 7-day surveillance period, participants will be asked to come to the BMP clinic center on days 14 +/- 3 and 30 +/- 7 after vaccination. At each visit, a study physician will evaluate the participants. A complete clinical examination will be performed and information on any solicited or unsolicited symptoms since the last visit will be collected. Every effort will be made to ensure compliance with visits. Local guides will conduct home visits to participants a day before their scheduled visit at the clinic. If a participant does not appear for a scheduled clinic visit, the local guide will visit him/her again and accompany the participant to the clinic center. If a serious adverse event (SAE) has occurred, appropriate measures will be taken to notify the PI, Local Medical Monitor, SMC, and all sponsors and IRBs as described in section 10.5.2.

7.1.5 Dose escalation

As soon as possible after the completion of the 7-day surveillance period following each immunization with the 25 µg dose of FMP2.1, the PI, Local Medical Monitor, at least two members of the SMC, and the Study Co-Sponsor (DMID) will review safety results and decide whether to proceed with next scheduled immunization with the full dose. Ultimately, the decision to proceed with each dose escalation will be made by the Study Co-Sponsor based on advice from the Local Medical Monitor and SMC and in consultation with WRAIR, GSKBio and investigators as described in detail in Section 15.4

7.1.6 Detailed description of study visits

Day -35 to –1 Screening /inclusion of participants

Meetings will be held with town administrative and medical authorities to explain the purpose of the study. These will be followed by meetings with the traditional authorities and the heads of families for village-level “permission to enter.” Subsequently, general information about the study will be disseminated through the local radio station. The target population will be invited for screening as described in investigator’s subject recruiting SOPs. Screening will be performed until 60 volunteers are included.

Visit 1 (may take place over more than one visit)

- Written informed consent for screening
- Medical history of participant
- Complete physical examination
- Collect 5-10 mL venous blood sample to measure:
  - Hematology: Complete Blood Count (CBC) which includes: red blood cells, platelets, white blood cell count, lymphocyte count, hemoglobin, mean corpuscular volume and mean corpuscular hemoglobin concentration.
  - Biochemistry: serum creatinine and ALT
- Beta-HCG pregnancy test on urine for females
- Collect urine: dipstick for blood, glucose and protein.
- Check of inclusion and exclusion criteria
- Written informed study consent for vaccination
- Assignment of unique study number
- Prepare an ID card containing participant's unique study number and photo for enrolled participants.

Day 0: Vaccination 1
Visit 2

Before vaccination:

- Review screening laboratory test results
- Review inclusion/exclusion criteria and check of contraindications/precautions
- Record any complaints, symptom-directed physical examination, and examination of the immunization arm(s) for any abnormalities.
- Record vital signs: oral temperature, blood pressure, pulse
- Record baseline data for solicited general symptoms
- Collect 30-40 mL venous blood sample to measure:
  - CBC, hemoglobin electrophoresis, creatinine, ALT
  - Serum for anti-AMA1 antibody titer and GIA
  - WBC for CMI to AMA1
- Collect urine for β-HCG test for females
- Administer study immunization 1

After vaccination:

- Observe for a minimum of 30 minutes
- Record blood pressure, pulse, oral temperature
- Record solicited and unsolicited events
- Instruct participants to return to the BMP clinic center immediately should they manifest any signs or symptoms they perceive as serious.

Days 1-3: First 3 days post-vaccination 1 surveillance visits
Visit 3-5

- Record vital signs; blood pressure, pulse, oral temperature
- Examine site of injection
- Record solicited and unsolicited signs/symptoms

- Targeted physical examination including immunization arm(s)

Day 7± 1 day: 7 days post-vaccination 1 surveillance visit
Visit 6

- Brief medical history
- Record vital signs; blood pressure, pulse, oral temperature
- Physical examination
- Record any unsolicited adverse events occurring after the last study immunization
- Collect 5-10 mL of venous blood to measure CBC, creatinine and ALT

Day 14± 3 days: 14 days post-vaccination 1 surveillance visit
Visit 7

- Brief medical history
- Record vital signs; blood pressure, pulse, oral temperature
- Physical examination
- Record any unsolicited adverse events occurring after the last study immunization
- Collect 30-40 mL of venous blood to measure:
    - Serum for anti-AMA1 antibodies and GIA
    - WBC for CMI

Day 30± 7 days: 1 month post-vaccination 1 surveillance visit and vaccination 2
Visit 8

Before vaccination:

- Check participant's ID to confirm identity
- Targeted physical examination including immunization arm(s)
- Check of contraindications/precautions
- Record vital signs: oral temperature, blood pressure, pulse
- Review medical history and record any unsolicited adverse events occurring since last visit
- Record baseline data for solicited general symptoms
- Collect 30-40 mL venous blood sample to measure:
    - CBC, creatinine, ALT
    - Serum for anti-AMA1 antibody titer and GIA
    - WBC for CMI
- Collect urine for β-HCG test for females
- Administer study immunization 2

After vaccination:

- Observe for at least 30 minutes
- Record blood pressure, pulse, oral temperature
- Record solicited and unsolicited adverse events
- Instruct participants to return to the BMP clinic center immediately should they manifest any signs or symptoms they perceive as serious.

Days 31-33: Days 1,2,3 post-vaccination 2 surveillance visits

Visits 9-11

- Record vital signs; blood pressure, pulse, oral temperature
- Examine site of injection
- Record daily solicited and unsolicited signs/symptoms
- Targeted physical examination including immunization arm(s)

Day 37 ± 7 days: Day 7 post-vaccination 2 surveillance visit
Visit 12

- Record vital signs; blood pressure, pulse, oral temperature
- Examine site of injection
- Record daily solicited and unsolicited signs/symptoms
- Targeted physical examination including immunization arm(s)
- Collect 5-10 mL of venous blood to measure CBC, creatinine and ALT

Day 44± 7 days: 14 days post-vaccination 2 surveillance visit
Visit 13

- Brief medical history
- Record vital signs; blood pressure, pulse, oral temperature
- Physical examination
- Record any unsolicited adverse events occurring after the last study immunization
- Collect 30-40 mL of venous blood to measure:
    - Serum for anti-AMA1 antibodies and GIA
    - WBC for CMI

Day 60± 7 days: 1 month Post-vaccination 2 surveillance visit and vaccination 3
Visit 14

Before vaccination:

- Check participant's ID to confirm identity
- Targeted physical examination including immunization arm(s)
- Check of contraindications/precautions
- Record vital signs: oral temperature, blood pressure, pulse
- Review medical history and record any unsolicited adverse events occurring since last visit
- Record baseline data for solicited general symptoms
- Collect 30-40 mL venous blood sample to measure:
    - CBC, creatinine, ALT
    - Serum for anti-AMA1 antibody titer and GIA
    - WBC for CMI
- Collect urine for β-HCG test for females
- Administer study immunization 3

After vaccination:

- Observe for at least 30 minutes
- Record blood pressure, pulse, oral temperature

- Record solicited and unsolicited adverse events
- Instruct participants to return to the BMP clinic center immediately should they manifest any signs or symptoms they perceive as serious.

Days 61-63: Days 1,2,3 post-vaccination 3 surveillance visits
Visit 15-17

- Record vital signs; blood pressure, pulse, oral temperature
- Examine site of injection
- Record daily solicited and unsolicited signs/symptoms
- Targeted physical examination including immunization arm(s)

Day 67 ± 7 days: Day 7 post-vaccination 3 surveillance visit
Visit 18

- Record vital signs; blood pressure, pulse, oral temperature
- Examine site of injection
- Record daily solicited and unsolicited signs/symptoms
- Targeted physical examination including immunization arm(s)
- Collect 5-10 mL of venous blood to measure CBC, creatinine and ALT

Day 74± 7 days: 14 days Post-vaccination 3 surveillance visit
Visit 19

- Brief medical history
- Record vital signs; blood pressure, pulse, oral temperature
- Targeted physical examination including immunization arm(s)
- Record any unsolicited adverse events occurring after the last study immunization
- Collect 30-40 mL of venous blood to measure:
    - Serum for anti-AMA1 antibodies and GIA
    - WBC for CMI

Day 90± 10 days: 30 days Post-vaccination 3 surveillance visit
Visit 20

- Brief medical history
- Record vital signs; blood pressure, pulse, oral temperature
- Targeted physical examination including immunization arm(s)
- Record any unsolicited adverse events occurring since last visit
- Collect 30-40 mL of venous blood to measure:
    - Serum for anti-AMA1 antibodies and GIA
    - WBC for CMI
    - CBC, creatinine and ALT
- Collect urine for β-HCG test for females

Day 120± 10 days to Day 364± 10 days:
Post-vaccination Safety surveillance period

- From this date, participants will be visited monthly by local guides to confirm their location and to remind them to come to clinic for routine clinical evaluation.

- Participants are invited to continue to attend the BMP clinic any time they are sick.

Day 180± 14 days to Day 364± 14 days:

Post-vaccination Safety surveillance period

Visit 21-23

- From this day participants will be asked to return to BMP clinic center every 3 months ±14 days.
- Brief medical history
- Record vital signs
- Targeted physical examination including immunization arm(s)
- Collect 30-40 mL venous blood to measure:
  - Serum for anti-AMA1 antibodies and GIA
  - WBC for CMI
  - CBC, ALT, creatinine
- Collect urine for β-HCG test for females
- Participants are invited to continue to attend the BMP clinic any time they are sick. A malaria smear will be done whenever symptomatic malaria is suspected.

7.1.7 Outline of study procedures

Table 3. Summary of study procedures

**Study Days**	**-35 to –1 Screening**	**0**	**1-3**	**7**	**14**	**30**	**31-33**	**37**	**44**	**60**	**61-63**	**67**	**74**	**90**	**120-364**
**Clinic Visit**	**1**	**2**	**3-5**	**6**	**7**	**8**	**9-11**	**12**	**13**	**14**	**15-17**	**18**	**19**	**20**	**21-23**
Village & family information & discussion	●														
Written individual Screening Consent	●														
Check of inclusion/exclusion criteria	●	●													
Check of contraindications to immunization		●				●				●					
Written individual Study Consent		●													
Medical history	●	●	●	●	●	●	●	●	●	●	●	●	●	●	●
Physical examination	●	●	●	●	●	●	●	●	●	●	●	●	●	●	●
Vital signs (T, BP, P)	●	●[f]	●	●	●	●[f]	●	●	●	●[f]	●	●	●	●	●
**Vaccination**		●				●				●					
Post-vaccination recording of solicited AE		●	●	●		●	●	●		●	●	●			
Recording of unsolicited AE occurring up to one month post-vaccination			●	●	●	●	●	●	●	●	●	●	●	●	
Recording of medication		●	●	●	●	●	●	●	●	●	●	●	●	●	●
Recording of SAEs during the study period		●	●	●	●	●	●	●	●	●	●	●	●	●	●
Urine analysis for blood, glucose and protein	●[a]														
Urine β-HCG	●[a]	●[b]				●[b]				●[b]				●	●[d]
CBC	●[a]	●[c]		●	●	●[c]		●	●	●[c]		●	●	●	●[d]
Serum chemistry (Creatinine, ALT)	●[a]	●[c]		●	●	●[c]		●	●	●[c]		●	●	●	●[d]
Serum and cells for anti-AMA1 response		●[c]			●	●[c]			●	●[c]			●	●	●[e]
Monthly home visit by local guides															●[g]
Review of health status															●[h]
Scheduled blood volume (mL)	5-10	30-40	0	5-10	30-40	30-40	0	5-10	30-40	30-40	0	5-10	30-40	30-40	90-120
Cumulative Blood Volume (mL)	5-10	35-50	35-50	40-60	70-100	100-140		105-150	135-190	165-230		170-240	200-280	230-320	320-440

a. Performed within 35 days prior to immunization
b. Performed just prior to each immunization
c. Blood collected just prior to each immunization
d. CBC, creatinine, ALT and urine β-HCG will be determined every 3 months from study Day 180 through study Day 364
e. Serum for anti-AMA1 antibodies and cells for CMI collected every 3 months from study Day 180 through study Day 364
f. Pre-immunization and 30mn after each immunization
g. Monthly local guides home visits to check the status of participants starting from study Day 120
h. Record any new onset chronic or acute diseases or other medically significant conditions, unscheduled clinic visits and any new treatments since previous scheduled study visit.

Table 4. Intervals between study visits

**Interval**	**Size of interval in days**
Visit 2 → Visit 7	14 ± 3
Visit 2 → Visit 8	30 ± 7
Visit 8 → Visit 13	14 ± 3
Visit 8 → Visit 14	30 ± 7
Visit 14 → Visit 19	14 ± 3
Visit 14 → Visit 20	30 ± 7

7.1.8 Health care provision

The clinical research facility includes private consultation rooms, a procedure room, a resuscitation suite with oxygen, suction and resuscitation kits, and a post-immunization observation room. An ambulance with suction and oxygen will be made available on immunization days. The pharmacy at the BMP clinic will have sufficient provisions to provide participants with oral and parenteral drugs for the treatment of common illnesses (including malaria) free of charge, using essential medicines and treatment regimens that meet or exceed standards recommended by the Mali Ministry of Health. 24-hour hospitalization and basic emergency surgery and obstetrical services are available at the Bandiagara Health Centre adjacent to the clinical facility. If the investigators or the Local Medical Monitor judge that a participant requires hospitalization at the regional hospital in Mopti (less than one hour's drive from Bandiagara), or at the National Hospital in Bamako (approximately 8 hours drive from Bandiagara), referral and transportation will be arranged and the medical management of the participants will be monitored by senior physician investigators and/or the Local Medical Monitor. The regional hospital in Mopti has blood transfusion capabilities, radiography, and more advanced medical subspecialty and surgical capabilities. The national hospital in Bamako has these capacities and in addition has an intensive care unit with mechanical ventilation, a computerized axial tomography scanning facility and other advanced medical and surgical care.

8 SAMPLE HANDLING AND ANALYSIS

8.1 Overview of collection time points

Blood will be collected from study participants by venipuncture up to 15 times during the study, including screening. The maximum amount of blood requested from any participant for standard collection during the study for research purposes will not exceed 440 mL over the one-year study period. However, additional blood may be obtained as deemed necessary by the investigators or clinicians to evaluate any illness or condition.

1. Safety

Tests for CBC, creatinine, and ALT, at screening, Day 0, 7, 14, 30, 37, 44, 60, 67, 74, 90 and every three months thereafter (intervals between study visits may deviate from this schedule by the ranges indicated in Table 4 above) will be performed at the BMP clinical laboratory.

2. Serology

Separation of serum/plasma from venous blood will be performed at the BMP clinic and samples will be aliquoted for later use to determine anti-AMA1 antibody levels and GIA at Day 0, 14, 30, 44, 60, 74, 90 and every three months thereafter (intervals between study visits may deviate from this schedule by the ranges indicated in Table 4 above) until the end of the study.

3. CMI

CMI assays will be performed on samples collected on study days 0, 14, 30, 44, 60, 74, 90 and every three months thereafter. The CMI assays on samples collected at day 90 and thereafter will provide information on the duration of vaccine-boosted CMI responses.

8.2 Laboratory Assays

The Investigators will maintain detailed SOPs for all laboratory assays at the BMP and central laboratories at MRTC, in Bamako. These SOPs will include sample collection, handling (e.g. serum separation for serology and GIA), labeling, preservation (e.g. PBMC cryopreservation), storage, transport, and shipping. All staff and investigators will be trained in the SOPs relevant to their duties and sign copies of the SOPs to document this training. Copies of SOPs will be available for inspection and review by the DMID, USAMMDA, GSKBio, WRAIR and study monitors. The general methods that will be used are summarized in the following section.

Hematology and Biochemistry:

A complete blood count (CBC), serum creatinine and ALT tests will be measured at defined time points throughout the study period. The Principal Investigator will maintain laboratory reference intervals in the study file, and copies will be made available upon request to study monitors and sponsors. Hematology and serum biochemistry assays will be performed at the BMP clinical laboratory in Bandiagara, which has passed inspection by monitors from USAMMDA, WHO and GSKBio.

Urine will be collected for glucose, blood and protein determination using FDA-approved urinary reagent dipsticks. Pregnancy tests will be performed for women on urine samples using FDA-approved urine pregnancy test kits.

Serology:

Serological assays for anti-AMA1 antibodies will be performed at the WRAIR laboratories in Silver Spring, US. Serum will be collected at indicated time points (see section 8.1).

Immunogenicity will be determined by evaluating antibody (IgG) responses to the *P. falciparum* AMA1 protein as measured using standard ELISA methodologies with appropriate capture antigens.

Additional assays

GIA: As described in detail in Section 4.3.1, antibodies to AMA1 inhibit merozoite invasion of erythrocytes in vitro and provide a secondary means of measuring specific response to immunization with the AMA recombinant antigen. Sera for growth inhibition assays and processing inhibition assays will be preserved and shipped to the WRAIR Department of Immunology.

CMI: Native AMA1 is known to shed from merozoites and is therefore processed by antigen-presenting cells. Recombinant AMA1 may or may not be seen by the immune system in the same way. Lymphocyte proliferation studies will be performed on PBMCs from pre- and post-immunization venous blood samples to determine whether individuals have naturally

acquired baseline cellular immune responses to AMA1 and whether these responses are boosted following immunization with the FMP2.1/AS02A vaccine. As noted in Section 4.3.1, AMA1 is also expressed in sporozoites and present in late stage liver schizonts, stages where CMI is important, providing additional rationale for CMI studies. PBMCs will be cryopreserved and transported to the University of Maryland or WRAIR Department of Immunology for these assays.

As a capacity-building exercise, WRAIR investigators will assist MRTC investigators with establishing the ability to perform serological assays for antibody (IgG) responses to the *P. falciparum* AMA1 by using the same ELISA methodologies with appropriate capture antigens that will be used for the immunogenicity study endpoint at WRAIR.

No additional blood will be drawn for these capacity-building assays. After final serological results from the reference immunology laboratory at WRAIR have been fully analyzed and reported, the results of the Mali assays may be compared to the WRAIR serological results, solely for the purposes of assessing how well the Malian laboratory was able to replicate the WRAIR results. It is emphasized that this is a capacity-building exercise and that only the serological results from the WRAIR Department of Immunology will be analyzed as trial endpoints.

9 STUDY VACCINES/MEDICATIONS AND ADMINISTRATION

9.1 Study vaccines

9.1.1 FMP2.1/AS02A vaccine

The candidate antigen, FMP2.1, has been developed and manufactured by the WRAIR. The adjuvant AS02A is manufactured by GSKBio. The Quality Control Standards and Requirements for each component of the vaccine are described in separate release protocols and the required approvals have been obtained. The vials will be stored at 2ºC to 8ºC.

FMP2.1

Each vial of the FMP2.1 antigen contains approximately 50 µg of lyophilized protein.

Active Ingredient: 0.100 mg/mL Purified Bulk PfAMA1 (3D7) in approximately 0.60 mL volume

Excipients:
23.5 mM Sodium phosphate
0.1 mM EDTA
3.15% Sucrose
30 mM Sodium chloride

AS02A adjuvant

The FMP2.1 vaccine will be reconstituted in AS02A adjuvant. AS02A contains 50 µg MPL and 50 µg QS21, 250 µl of SB62 (oil/water emulsion) in phosphate buffered saline (PBS) per volume of 0.5 mL. The AS02A adjuvant will be supplied as pre-filled syringes. The prefilled syringes will contain approximately 0.60 mL of liquid and will be stored at 2ºC to 8ºC.

FMP2.1/AS02A vaccine

Each vial of FMP2.1, containing approximately 50 µg antigen, will be reconstituted using one pre-filled syringe containing approximately 0.6 mL AS02A adjuvant.

9.1.2 RabAvert® vaccine

RabAvert, rabies vaccine, produced by Chiron Behring GmbH & Co is a sterile freeze-dried vaccine obtained by growing the fixed-virus strain Flury LEP in primary cultures of chicken fibroblasts. The strain Flury LEP was obtained from American Type Culture Collection as the 59th egg passage. The growth medium for propagation of the virus is a synthetic cell culture medium with the addition of human albumin, polygeline (processed bovine gelatin) and antibiotics. The virus is inactivated with â-propiolactone, and further processed by zonal centrifugation in a sucrose density-gradient. The vaccine is lyophilized after addition of a stabilizer solution which consists of buffered polygeline and potassium glutamate. One dose of reconstituted vaccine contains less than 12 mg polygeline (processed bovine gelatin), 1 mg potassium glutamate and 0.3 mg sodium EDTA. Small quantities of bovine serum are used in the cell culture process. Bovine components originate only from source countries known to be free of bovine spongiform encephalopathy. Minimal amounts of chicken protein may be present in the final product; ovalbumin content is less than 3 ng/dose (1 mL), based on ELISA. Antibiotics (neomycin, chlortetracycline, amphotericin B) added during cell and virus propagation are largely removed during subsequent steps in the manufacturing process. In the final vaccine, neomycin is present at < 1 µg, chlortetracycline at < 20 ng, and amphotericin B at < 2 ng per dose. RabAvert is intended for intramuscular (IM) injection. The vaccine contains no preservative and should be used immediately after reconstitution with the supplied Sterile Diluent for RabAvert (Water For Injection). The potency of the final product is determined by the NIH mouse potency test using the US reference standard. The potency of one dose (1.0 mL) RabAvert is at least 2.5 IU of rabies antigen. RabAvert is a white, freeze-dried vaccine for reconstitution with the diluent prior to use; the reconstituted vaccine is a clear to slightly opaque, colorless suspension.

9.2 Dosage and administration

The Investigators will maintain detailed SOPs for vaccine transport, storage, formulation, reconstitution and administration. All staff and investigators will be trained in the SOPs relevant to their duties and sign copies of the SOPs to document this training. Copies of SOPs will be available for inspection and review by the DMID, USAMMDA, GSKBio, WRAIR and study monitors. The general methods that will be used are summarized in the following section.

9.2.1 Formulation of FMP2.1 with AS02A

After it has been determined that a participant can be immunized, the AS02A contents of one pre-filled syringe will be injected into a vial of lyophilized vaccine. The pellet of FMP2.1 will be dissolved before withdrawing either 0.25 mL of FMP2.1/AS02A (for the 25 µg group) or 0.5 mL of FMP2.1/AS02A (for the 50 µg group). (See also section 9.4 for instructions on masking the syringe contents.) Because the exact amount of FMP2.1 antigen administered is

determined by the volume drawn into the syringes and may therefore vary by very small amounts, doses are referred to as "approximately" 25 µg and 50 µg elsewhere in the protocol.

9.2.2 Reconstitution of RabAvert® vaccine

After it has been determined that a participant can be immunized, using the longer of the 2 needles supplied, the entire contents of the diluent vial will be transferred into the vaccine vial and mixed gently to avoid foaming. The white, freeze-dried vaccine dissolves to give a clear or slightly opaque suspension. The total amount of dissolved vaccine will be withdrawn into the syringe and long needle will be replaced with the smaller needle for IM injection. The reconstituted vaccine will be used immediately. (See also section 9.4 for instructions on masking the syringe contents).

9.2.3 Administration of vaccines

Each 0.25 or 0.5 mL of FMP2.1/AS02A or 1.0 mL of RabAvert® Rabies vaccine will be administered slowly by intramuscular injection in the left deltoid muscle immediately after formulation/reconstitution. Alternatively the right deltoid muscle could be used when the preferred site for injection is contraindicated or not advisable such as in the case of severe pain, infection, scarring that will make local reactions difficult to assess, or if the study participant declares a preference to be immunized in the alternative site.

9.3 Vaccine storage and accountability

FMP2.1 and RabAvert® vaccines and AS02A pre-filled syringes will be stored at + 2 ºC to + 8 ºC. Vaccines will be kept in a refrigerator that has 24-hour temperature recording. A back-up refrigerator and generator will be available in case of breakdown/power failure. The refrigerator that holds the vaccines and adjuvant will be locked. The field site manager and the study coordinator will keep the keys. Records will be maintained that document receipt, release for immunization, disposal or return to the manufacturer of all vaccine vials. Copies of these records will be provided to the sponsors for archiving. All study records will be kept in locked metal boxes.

The FMP2.1 antigen vials will be transported under controlled temperature conditions to the MRTC in Bamako, Mali. Temperature recorders will document maintenance of required temperature ranges. The AS02A adjuvant will be similarly transported from Belgium. FMP2.1 antigen, comparator vaccine and adjuvant will be stored in a cold room in the MRTC laboratory in Bamako until a few days before each vaccination is scheduled in Bandiagara. These will be transported in temperature-controlled conditions from Bamako to Bandiagara in the same containers used for shipping to Mali. In Mali, only the vaccine manager and assistant vaccine manager will have access to vaccines. The Vaccine Log Book will also be used to record use and final disposition of each vial of FMP2.1 and adjuvant. Used vaccine vials, as well as unused vials, will be kept, until such time as the PI and sponsors agree that there are no concerns about vaccine accountability and that they can be discarded.

9.4 Methods of blinding and breaking the study blind

9.4.1 Double blinding

The FMP2.1 antigen and the AS02A adjuvant will be packaged separately. The FMP2.1 antigen and the AS02A adjuvant will have exactly the same milky white appearance. After reconstitution, the comparator rabies vaccine will appear clear to slightly opaque and colorless. Blinding of the individual preparing the study vaccine ("drug manager") will not be possible. Since the test article and comparison vaccines can be distinguished by appearance, the vaccine preparation area and the immunizing area will be physically separated. The drug manager, an experienced pharmacist, will be exclusively dedicated to vaccine preparation. He will have assigned to him an assistant drug manager to ensure that the proper vaccine is delivered for each participant. To determine which vaccine each participant will receive, the drug manager will refer to the unique randomization code assigned to that participant's unique study number. The drug manager will check the study number on the participant's photo ID and will make sure that it matches that in the CRF. The drug manager will then refer to a key matching the randomization code assigned to that participant to verify the vaccine to be administered. He will also confirm that the randomization code of the participant matches the vaccine to be given in the key list. The vials will be reconstituted as above and the vaccine will be drawn into a 1 mL syringe. The barrel of the syringe will be covered with opaque tape to hide its contents.

Immunizations will be carried out simultaneously in one of two separated rooms adjacent to the vaccine preparation room and connected by sliding doors. The vaccine-filled syringes will be passed through the sliding doors to vaccinators who will consist of clinician co-investigators who will not be involved with surveillance assessments.

Despite the fact that the volumes of the three study vaccine preparations are different, every attempt will be made to maintain blinding. First, the syringe barrels will be covered with opaque tape. Second, the injectors of the study vaccines will be investigators who are not involved in any way with surveillance activities, so that even if they realize which vaccine they are injecting, they will not be involved in the assessment of adverse events following vaccination. We and many WRAIR investigators have used this technique in recent trials and we concede the possibility that a very astute participant could discern how far syringe plungers extend from the barrel, and potentially discuss this later with other participants. Mitigating this concern is the practice of injecting each participant in a closed room out of view of anyone other than the investigator(s) injecting the vaccine, so that each participant sees only the syringe he or she is injected with and never sees other participants being injected. Furthermore, the participants are not told that the vaccines vary with respect to volume, so it is very unlikely that they will discuss this amongst themselves. Finally, during the previous Phase 1 malaria vaccine trial in adults at this site, we have observed that almost without exception, participants averted their eyes at the time of immunization and thus did not directly observe the syringe.

9.4.2 Breaking the study blind

A participant's study randomization code may be unblinded before study day 90 only for safety purposes. This procedure is therefore exceptional and any decision to unblind will be discussed with the sponsors, the PI, the Senior Co-Investigators, the Local Medical Monitor, and the SMC. If deemed necessary for urgent safety reasons, the Local Medical Monitor, in consultation with the SMC, may unblind a specific participant without revealing the study blind

to the investigators and the sponsors. Any opening of these coded envelopes will be documented according to investigator SOPs. It is to be emphasized that the Local Medical Monitor and SMC may put the study on hold at any time and discuss safety concerns with the sponsors. The decision to completely unblind or permanently stop the study, will take the final form of a formal recommendation by the SMC to the study co-sponsor and IND sponsor, who will make this determination and communicate it to the PI. The PI will then notify the IRBs of this decision.

Final unblinding will be done only after monitoring/verification of GCP compliance by the study monitors, SMC review of safety concerns, and after all safety and immunological results up to study day 90 have been entered and the study statistician certifies that the databases have been locked. The final decision to unblind will be made jointly by USAMMDA and DMID.

9.5 Concomitant medication/Treatment

At each study visit/contact, the investigator will question the participant about any medication taken, including traditional medicines. Concomitant medication, including any vaccine other than the study vaccines, and any other medication relevant to the protocol, including any specifically contraindicated or administered during the period starting from one week before each study immunization and ending one month (maximum 30 days) after will be recorded in the CRF with trade name and/or generic name of the medication, medical indication, start and end dates of treatment.

10 ADVERSE EVENTS

It is the responsibility of the investigators to document all adverse events according to the detailed guidelines set out below. The participants will be instructed to contact the investigator immediately should they manifest any signs or symptoms that they perceive as serious during the study.

10.1 Eliciting and documenting adverse events

10.1.1 Adverse event definition

An adverse event includes any noxious, pathological or unintended change in anatomical, physiological or metabolic functions as indicated by physical signs, symptoms and/or laboratory detected changes occurring in any phase of the clinical study whether associated with the study vaccine or active comparator vaccine and whether or not considered vaccination related. This includes an exacerbation of pre-existing conditions or events, intercurrent illnesses, or vaccine or drug interaction. Symptomatic uncomplicated or severe malaria infection will be coded as adverse events as will any other acute illness, throughout the 12 months of the study. Anticipated day-to-day fluctuations of pre-existing conditions that do not represent a clinically significant exacerbation need not be considered adverse events. Discrete episodes of chronic conditions occurring during a study period will be reported as adverse events in order to assess changes in frequency or severity.

Adverse events will be documented in terms of a medical diagnosis. When this is not possible, the adverse event will be documented in terms of signs and/or symptoms observed by the investigator or reported by the subject at each study visit. Pre-existing conditions or signs

and/or symptoms (including any which are not recognized at study entry but are recognized during the study period) present in a participant prior to the start of the study will be recorded on the participant's CRF. Any hospitalization will be considered a serious adverse event. Adverse events to be recorded as endpoints are described in Section 10.1.4. All other adverse events will be recorded as unsolicited adverse events.

10.1.2 Surveillance period for adverse events

All adverse events occurring within 30 days following administration of each study immunization will be recorded irrespective of severity or whether or not they are considered vaccination-related.

Solicited adverse events will be elicited for a 7-day surveillance period (day of vaccination and study days 1, 2, 3 and 7 and unsolicited adverse events will be recorded during a 30-day surveillance period. Serious adverse events will be recorded throughout the study.

Instances of death, cancer or congenital abnormality in offspring of a study subject if brought to the attention of the investigator at any time after cessation of study AND suspected by the investigator to be related to study vaccine, will be reported to the sponsors and GSK Biologicals.

10.1.3 Recording adverse events

At each visit/assessment, the investigator will evaluate all adverse events observed by the investigators or reported by the participant. New adverse events will be recorded in the Adverse Event form within the participant's CRF. Solicited and unsolicited adverse events will be recorded on separate pages of the CRF. The nature of each event, date and time (where appropriate) of onset, outcome, intensity and relationship to vaccination will be established. Any corrective treatment will be recorded in the CRF. As a consistent method of soliciting adverse events, the participant will be asked a non-leading question such as: "Have you felt different in any way since receiving the vaccine or since the last visit?" The investigator will record only those adverse events having occurred within the time frames defined above.

Adverse events already documented in the CRF, *i.e.* at a previous assessment and designated as 'ongoing' will be reviewed at subsequent visits, as necessary. If these have resolved, the documentation in the CRF will be completed, including the date that the adverse event resolved. If an adverse event changes in frequency or intensity during a study period, a new record of the event will be started.

10.1.4 Solicited adverse events

Local (injection site) adverse events
Pain at injection site
Swelling at injection site
Erythema
Limitation of arm motion (abduction of shoulder)

General adverse events
Fever (oral body temperature ≥ 37.5°C)
Chills
Nausea
Headache
Malaise
Myalgia
Joint pain

Oral temperature will be recorded at the time of the clinic visit. If additional temperature measurements are recorded at another time of the day, the highest temperature will be recorded.

The assessment of severity/intensity will be as described in Section 10.2. For general signs and symptoms reported, the investigators will assign causality as described in Section 10.3 For all signs and symptoms reported, the investigators will report the outcome as described in Section 10.5.2.

10.1.5 Unsolicited adverse events

Unsolicited adverse events will be recorded in the CRF. Unsolicited adverse events are adverse events reported by the participants that are different from those solicited symptoms defined in the preceding section and/or that begin after the 7-day surveillance period for solicited adverse events.

10.2 Assessment of intensity of non-serious adverse events

For each solicited symptom the participants will be asked if they sought medical advice for this symptom. For all other adverse events than those in Table 5, maximum intensity will be assigned to one of the following categories:

0 = No adverse event

1 = An adverse event which is easily tolerated by the subject, causing minimal discomfort and not interfering with everyday activities.

2 = An adverse event that is sufficiently discomforting to interfere with normal everyday activities.

3 = An adverse event that prevents normal, everyday activities. Such an adverse event would for example prevent attendance at work/school and would require the administration of corrective therapy.

Intensity of the following adverse events will be assessed as described in Table 5:

Table 5: Assessment of adverse event intensity

Adverse Event	Intensity grade	Intensity definition
Pain at injection site	0	None
	1	Pain that is easily tolerated
	2	Pain that interferes with daily activity
	3	Pain that prevents daily activity
Swelling at injection site<br>Record size	0<br>1<br>2<br>3	0 mm<br>>0 - ≤ 20 mm<br>>20 - ≤ 50 mm<br>>50 mm
Erythema at injection site<br>Record size	0<br>1<br>2<br>3	0 mm<br>>0 - ≤ 20 mm<br>>20 - ≤ 50 mm<br>>50 mm
Limitation of arm motion - Abduction at the shoulder	0	None
	1	>90° but <120°
	2	>30° but ≤90°
	3	≤30°
Fever<br>Record oral temperature	0<br>1<br>2<br>3	<37.5ºC<br>37.5 - ≤38.0ºC<br>>38.0 - ≤39ºC<br>>39ºC
Chills	0	None
	1	Chills that are easily tolerated
	2	Chills that interfere with daily activity
	3	Chills that prevent daily activity
Nausea	0<br>1<br>2<br>3	None<br>Nausea that is easily tolerated<br>Nausea that interferes with daily activity<br>Nausea that prevents daily activity
Headache	0	None
	1	Headache that is easily tolerated
	2	Headache that interferes with daily activity
	3	Headache that prevents daily activity
Malaise	0	None
	1	Malaise that is easily tolerated
	2	Malaise that interferes with daily activity
	3	Malaise that prevents daily activity
Myalgia	0	None
	1	Myalgia that is easily tolerated
	2	Myalgia that interferes with daily activity
	3	Myalgia that prevents daily activity
Joint pain	0	None
	1	Joint pain that is easily tolerated
	2	Joint pain that interferes with daily activity
	3	Joint pain that prevents daily activity

10.3 Assessment of causality

Every effort will be made by the investigator to explain each adverse event and assess its causal relationship, if any, to administration of the study vaccine(s).

The degree of certainty with which an adverse event can be attributed to administration of the study vaccine(s) (or alternative causes, e.g., natural history of the underlying diseases, concomitant therapy, etc.) will be determined by how well the event can be understood in terms of one or more of the following:

- Reaction of similar nature having previously been observed with this type of vaccine and/or formulation.
- The event having often been reported in literature for similar types of vaccines.
- The event being temporally associated with vaccination or reproduced on re-vaccination.

All solicited local (injection site) reactions will be considered causally related to vaccination. The investigators will assess the causality of all other adverse events using the following method:

In your opinion, did the vaccine(s) possibly contribute to the adverse event?

NO : The adverse event is not causally related to administration of the study vaccine(s). There are other, more likely causes and administration of the study vaccine(s) is not suspected to have contributed to the adverse event.

YES : There is a reasonable possibility that the vaccine contributed to the adverse event.

If an AE is determined not to be causally related to administration of a study vaccine, the investigators will specify the likely cause if one can be determined.

10.4 Following-up of adverse events and assessment of outcome

Investigators will follow up subjects with serious adverse events until the event has resolved or until the condition has stabilized regardless of when this occurred in relation to the study conclusion. Investigators will follow up participants with non-serious adverse events until the participant completes the study. Clinically significant laboratory abnormalities, as well as any adverse event, will be followed up until they have returned to normal, or until a satisfactory explanation has been provided. Reports relative to the subsequent course of an adverse event noted for any subject will be submitted to the Study Monitor.

Outcome will be assessed as:
1 = Recovered
2 = Recovered with sequelae
3 = Ongoing at participant study conclusion
4 = Died
5 = Unknown

10.5 Serious adverse events

10.5.1 Definition of a serious adverse event

A serious adverse event is any untoward medical occurrence that results in death, is life threatening, results in persistent or significant disability/incapacity, requires in-patient hospitalization or prolongation of existing hospitalization or is a congenital anomaly/birth defect in the offspring of a study subject. In addition, important medical events that may jeopardize the

patient or may require intervention to prevent one of the other outcomes listed above will be considered serious.

- Life threatening—definition: An adverse event is life threatening if the participant was at risk of death at the time of the event; it does not refer to an event, which hypothetically might have caused death, if it were more severe.

- Disabling/incapacitating—definition: An adverse event is incapacitating or disabling if the event results in a substantial disruption of the participant's ability to carry out normal life functions. This definition is not intended to include experiences of relatively minor medical significance such as headache, nausea, vomiting, diarrhea, influenza, and accidental trauma (e.g. sprained ankle).

- Hospitalization: In general, hospitalization signifies that the participant has been detained (usually involving at least an overnight stay) at the hospital or emergency ward for treatment that would not have been appropriate in the physician's office or outpatient setting.

10.5.2 Reporting serious adverse events

In the event that one or more serious adverse reactions probably or suspected of being related to vaccination are detected following any immunization in any of the vaccine groups, no further vaccinations will be administered until a written report has been submitted to DMID, U.S. Army HSRRB, University of Maryland IRB, and the FMPOS Ethics Review Committee and the investigators have conferred with the Local Medical Monitor and SMC.

Any serious adverse events occurring during the study period whether or not considered to be related to the study vaccine/comparator will be reported within 24 hours of the PI being notified to the IND sponsor (USAMMDA) by telephone, followed by faxing of the complete AE reporting form to the Sponsor. SAEs will also be reported within 24 hours by telephone or E-mail to the Local Medical Monitor, SMC and the FMPOS Ethics Review Committee in Mali. Any death or SAE that is related to the study vaccine will be reported to DMID and GSK-Biologicals within 24 hours of the PI being notified. A written report will follow the initial report within 3 working days. The report will be sent to the Quality Assurance Office, USAMMDA (cf. Appendix 17.2)

Notifications and reports will be provided by USAMMDA to the following agencies by Internet, e-mail, fax or telephone within the reporting deadlines required by each agency: the co-sponsor (DMID), the University of Maryland School of Medicine IRB, the Food and Drug Administration, and the HSRRB. All SAE reports and follow-up information will be reported to GSK-Biologicals by fax, phone, or E-mail within 24 hours of the PI becoming aware of the SAE. The rationale for USAMMDA serving as the primary contact point for disseminating all SAE reports to these bodies (with the exception of GSK Biologicals) is that communications at the field site are limited, and while every effort will be made to build redundancy into the communications systems, it is possible that phone land lines and E-mail could be down simultaneously, leaving only satellite phones as a means of communication from Bandiagara. The PI and co-PI on site will make every effort to directly notify all IRBs, sponsors and partners directly within their required reporting deadlines, in addition to the notifications they will receive from USAMMDA.

Every serious adverse event that is not resolved at the time the initial written report is filed will have a follow-up report submitted when information is available. Any submitted report will be identified as "initial", "follow-up", or "medical monitor".
The initial notification will include:

- The study protocol number and the name of the PI
- The participant study number, sex and age
- The date of onset of the SAE, and date of administration of study vaccine(s)

The PI will not wait to collect additional information to fully document the event before making notification of a serious adverse event. The telephone/e-mail report will be followed by a full written report using the SAE form within the CRF, detailing relevant aspects of the adverse events in question.

Instances of death, cancer or congenital abnormality in offspring of a study participant if brought to the attention of the investigator AT ANY TIME after cessation of the study AND suspected by the Investigators to be related to study medication will be reported to the sponsors and GSK Biologicals within four weeks of coming to the knowledge of the PI.

10.6 Pregnancy

Participants who become pregnant during the study period (up to 30 days after receiving the last study immunization) will not receive additional study immunizations but may continue other study procedures.

Female participants will be instructed to notify the investigators if they become pregnant at any time during the 12-month study period. Although not considered an adverse event pregnancy will be reported in the same way as an adverse event. All pregnancies occurring during the study period will be followed to term (with permission of the participant), any premature termination reported, and the health status of the mother and child including date of delivery and the child's gender and weight will be reported to HSRRB with copy to DMID and GSKBio.

10.7 Treatment of adverse events

The investigators will provide treatment of adverse events . Advice will be sought from the Local Medical Monitor and other medical specialists as indicated for severe, unusual or complicated medical conditions. Treatment events will be fully documented.

11 PARTICIPANT COMPLETION AND DROP-OUT

11.1 Definition

From the perspective of data analysis a 'drop-out' is any participant who did not come back for the concluding visit foreseen in the protocol. A participant who returns for the concluding visit foreseen in the protocol is considered to have completed the study.

11.2 Procedure for handling drop-outs

Investigators will attempt to contact those participants who missed scheduled surveillance visits. Information gathered will be described on the Study conclusion page of the CRF and on Medication/Adverse event forms as appropriate.

11.3 Reasons for drop-out

The Study conclusion page on the CRF will specify which of the following possible reasons were responsible for dropout of the participant from the study:

- Serious adverse event
- Non-serious adverse event
- Protocol violation (to be specified)
- Withdrawal of study consent, not due to an adverse event
- Migration from the study area
- Lost to follow-up
- Other (to be specified)

12 DATA MANAGEMENT AND ANALYSIS

Data entry will be performed on site and in the MRTC/MMVDU data management unit in Bamako if necessary. The MRTC data management unit will do data analysis and reporting for primary and secondary endpoints in collaboration with the CVD statistician.

12.1 Primary endpoints

Occurrence of solicited symptoms during a 7-day surveillance period after vaccination (day of vaccination and study days 1, 2, 3 and 7).

Occurrence of unsolicited symptoms during a 30-day surveillance period after vaccination.

Occurrence of serious adverse events during the study period.

12.2 Secondary endpoints

Anti-AMA1 antibody titers at time points at which blood samples are collected for serology.

Inhibition of parasite growth by the in vitro GIA to 3D7.

Specificity of the antibodies to diverse AMA1 genotypes in addition to 3D7, by measuring by ELISA, and GIA on parasites with typed AMA1.

12.3 Study cohorts/datasets to be evaluated

Total cohort

The 'Total Cohort' will include all participants enrolled (defined as randomized to study groups) in the study.

Safety cohort

The 'Safety Cohort' will consist of all participants who have received at least one dose of study vaccine or comparator and for whom any data on safety are available.

The presentation of safety data will explore separately the adverse experiences among participants who received all vaccinations, among those who received only some and among those with clinical violations of study protocol.

Immunogenicity cohort

The ‘Immunogenicity Cohort’ will include all evaluable participants (i.e., those meeting all eligibility criteria, and who have received at least one immunization with any of the study or control vaccines) for whom data concerning immunogenicity endpoint measures are available. This will include participants for whom assay results are available for antibodies against at least one study vaccine antigen component after vaccination.

12.4 Estimated sample size

This Phase 1 trial is not powered to detect differences between groups. Even if comparative statistics for the safety variables are computed, the study will have low power to detect anything other than very large differences in the incidence of local and general side effects between the vaccination groups. This is done weighing the need to detect any possible untoward reactions against the need to limit the number of volunteers involved for safety purposes. The sample size of 20 in each study group is widely accepted and used in industry for the initial assessment of the safety, tolerance and immunogenicity of an investigational vaccine. Incorporation of a comparator vaccine as control will enable broad initial estimates of the incidence of local and general side effects and of immune responses among vaccine recipients.

Table 6. Event detection probability table.

Event Rate	Pr 0/20	Pr 1+/20
0.01	0.82	0.18
0.05	0.36	0.64
0.08	0.19	0.81
0.10	0.12	0.88

Event rate. True rate at which an event occurs.
Pr 0/20. Given the event rate, probability that no events will be detected among 20 vaccinees.
Pr 1+/20. Given the event rate, probability that one or more events will be detected among 20 vaccinees.

If the SAE rate is 8%, then the probability of observing at least one event in a group size of 20 (Pr 1+/20) is 0.81. If the SAE rate is 8%, then the probability of observing no event in a group size of 20 (Pr 0/20) is 0.19.

12.5 Primary analysis

A final research analysis plan will be agreed upon by the investigators, DMID, WRAIR and GSKBio prior to locking of the database for the final analysis. This plan will include information on adverse events by dose and number of vaccinations. The primary analysis will be conducted on data collected until Day 90 (30 days post Immunization 3). The data generated at this time will be used for decision-making related to the product clinical development plan. The study will continue in a single blind manner for additional safety surveillance and immunogenicity assessment. This additional information will be appended to the study report. It is anticipated that the results of this study will be presented to the scientific community via oral presentations at meetings and written publications in scientific journals. The data to be presented and the authorship will be discussed between investigators and sponsors, and approved by the sponsors, prior to any official communication.

The official report of the primary analysis will be submitted through appropriate channels and upon approval by the WRAIR, Dept. of Immunology to the Human Use Review and Regulatory Affairs Division at Ft. Detrick, MD. This report will contain detailed information about the participants, their tolerance of the vaccines, their side effects and laboratory abnormalities, as well as their overall immune responses to immunization.

12.5.1 Analysis of demographics

Demographic characteristics (age, sex and place of residence) of each study cohort will be tabulated. The mean age (plus range and standard deviation) by sex of the enrolled participants, as a whole and per group will be tabulated.

12.5.2 Analysis of immunogenicity

Immunogenicity will be assessed in several ways. A series of graphs will display immunologic responses. For each vaccine group and time point, the distribution of anti-AMA1 antibody levels and reverse cumulative distribution curves will be plotted. Corresponding summary statistics will show means and standard deviations as well as median, 25th and 75th

percentiles, and 10th and 90th percentiles. The results will be presented both as raw data and as log-transformed data.

In addition, for each treatment group and time point, anti-AMA1 antibody levels will be presented as geometric means of OD units with 95% confidence intervals. For each vaccine group and for each time point, a table will show the proportion of volunteers with two-fold, four-fold, and eight-fold increases in anti-AMA1 antibody titers.

12.5.3 Analysis of safety

The overall percentage of participants with at least one local adverse event (solicited or unsolicited) and the percentage with at least one general adverse event (solicited and unsolicited) during the 7-day surveillance period after vaccination will be tabulated. The incidence, intensity and relationship of individual solicited symptoms over the 7-day surveillance period will be calculated per group and vaccine administration.

The number of participants with at least one report of an unsolicited adverse event, classified by WHO-preferred terms, reported up to 29 days after vaccination will be tabulated per group and vaccine administration. The intensity and relationship to vaccination of the unsolicited symptoms reported will also be assessed.

Serious adverse events are expected to be rare, but where observed will be described. Comparisons between study groups of incidence of symptoms, local and general symptoms will be made based on a two-sided Fisher's Exact Tests. Analysis of safety during the 12-month surveillance period will consist of comparison of incidence of serious adverse events as well as hemoglobin, creatinine and ALT levels.

Clinical laboratory parameters

Hematological (CBC) and biochemical (ALT, creatinine) laboratory parameters will be measured at specific time points, Days 0, 7, 14, 30, 37, 44, 60, 67, 74, 90 and starting on day 180 every 3 months. Clinically relevant abnormal values will be tabulated and a trend analysis could be performed if deemed necessary.

13 SITE MONITORING PLAN

Site monitoring may be conducted by DMID or its designated monitoring contractor, to ensure that GCP standards and regulatory guidelines are being followed. Pre-trial monitoring visits will be made to the site, including the clinical laboratory. All records will be made available to monitors, including regulatory files, CRFs and other source documents, QA/QC documentation, SOPs, etc. At the discretion of the monitor, additional site visits may be made during the course of the trial and at the end of the surveillance period.

In addition, monitors from USAMMDA, GSKBio and WHO will be welcome to make site visits, in coordination with the primary monitoring group designated by DMID.

In conjunction with the monitoring body designated by DMID, a detailed monitoring plan, subject to approval by OCRA, will be developed and included in the Manual of Procedures. The monitoring plan will include the number of subject charts to be reviewed, which/what proportion of data fields and what will be monitored, and who will be responsible for conducting the monitoring visits, and who will be responsible for ensuring that monitoring findings are addressed.

14 QUALITY CONTROL AND QUALITY ASSURANCE

SOPs for quality management will be developed, used to train appropriate personnel, and kept on file with documentation of training. Data will be evaluated for compliance with protocol and accuracy in relation to source documents. The study will be conducted in accordance with procedures identified in the protocol. The types of materials to be reviewed, who is responsible, and the schedule for reviews will be referenced in the SOPs. Study-specific training will be provided for all staff prior to the commencement of the trial.

The study will be conducted at a single center, the Bandiagara Malaria Project in Bandiagara, Mali, with the exception of immunological studies which will be conducted at WRAIR (serology, GIA and CMI), UMB (CMI) and the main campus of the University of Bamako (serology NOT to be used for study endpoints, and CMI).

SOPs will be used at all clinical and laboratory sites. Regular monitoring and an independent audit will be performed according to GCP/ICH (e.g., data monitoring). Following written standard operating procedures, the monitors will verify that the clinical trial is conducted and data are generated, documented (recorded), and reported in compliance with the protocol, GCP, and the applicable regulatory requirements. Reports will be submitted to DMID on monitoring activities.

The investigational site will provide direct access to all trial related sites, source data/documents, and reports for the purpose of monitoring and auditing by the sponsor, and inspection by local and regulatory authorities.

The MRTC Biostatistics and Epidemiology Unit (Data Coordinating Center) in Bamako will implement quality control procedures beginning with the data entry system and generate data quality control checks that will be run on the database. Any missing data or data anomalies will be communicated to the field site for clarification/resolution.

15 ETHICAL CONSIDERATIONS

15.1 Statement of compliance and ethical reviews

The WRAIR FMP2.1/ASO2A vaccine has been submitted as FDA IND BB-9202 and the study described in this protocol will be conducted according to current Good Clinical Practices (US 21 CFR Part 50-Protection of Human Subjects and Part 56-Institutional Review Boards, U.S. Army Regulation AR 40-38 and AR 70-25, U.S. 45 CFR 46, 21 CFR 312, and the applicable rules and regulations of Mali).

The FMPOS IRB (FWA00001769) will review and approve the protocol prior to study start. In addition, the study will be reviewed by the Human Subjects Research Review Board (HSRRB) of the Office of the Surgeon General, US Army, by DMID, and the University of Maryland IRB. Documentation of the approval by these ethical review boards will be kept in the PI's study file.

15.1.1 Institutional Review Boards

All amendments will be submitted to the University of Bamako Faculty of Medicine Pharmacy and Odonto-stomatology (FMPOS) IRB, the UMB IRBs, and the HSSRB as well as to the DMID as the study sponsor. No amendments will go into effect without written approval from the FMPOS IRB, the UMD IRB, HSRRB and DMID except when necessary to eliminate immediate hazards to the participants. Protocol deviations will also be reported to each IRB according to the policy of each IRB. CRFs and other source documents will be examined to

determine whether missing data were not transcribed, unavailable or missing for unknown reasons and this information will be coded and documented in the database.

The investigators will inform all the IRBs and DMID of the following:

- All subsequent protocol amendments, informed consent changes or revisions of other documents originally submitted for review
- Serious and/or unexpected adverse events occurring during the study, where required
- New information, including any provided by GSKBio, USAMMDA or WRAIR, that may affect adversely the safety of the participants or the conduct of the study
- An annual update and/or request for re-approval, where required
- When the study has been completed.

15.2 Informed consent

The principles of informed consent in the current edition of the Declaration of Helsinki will be implemented before any protocol-specified procedures or interventions are carried out.

Information will be given in both oral and written form whenever possible. The written consent documents will embody the elements of informed consent as described in the current edition of the Declaration of Helsinki, will adhere to the ICH Harmonized Tripartite Guideline for Good Clinical Practice and will also comply with applicable Malian regulations. Independent witnesses will be used to attest that illiterate potential participants have understood the contents of the informed consent.

15.2.1 Screening and study informed consent

We consider informed consent to be a dynamic, ongoing process, with continuous availability of investigators to answer any questions that arise in the course of the trial and to ensure that participants understand trial procedures. Should new data become available that could affect participant safety and/or willingness to continue in the study, informed consent would be obtained and documented again.

The extensive contact between the team of investigators and the population of Bandiagara has led to the development of mutual trust and the establishment of an ongoing informed consent process attempting to address issues related to interventional studies in resource-limited settings. Many discussions with local community leaders, heads of families and citizens through group meetings, and more limited group interviews have reviewed the need to obtain a written informed consent from study participants. The community has now become familiar with the informed consent process, including written, signed consent forms, which have been used for several studies at this site, including the ongoing Phase 1 malaria vaccine trial that began in 2003.

Prior to initiating any study in Bandiagara, the senior Malian and U.S. investigators visit the local commandant (representative of the national government), the mayor, the director of the local school system, the chiefs of each of the eight quartiers of Bandiagara, the medical director of the local health center, the director of the Bandiagara Center for Research of Traditional Medicine, and the head of the Bandiagara traditional healers' association. These are courtesy visits in which results of the previous year's studies are summarized and plans for new studies are explained and any questions are answered. In accordance with the tradition in Mali, small quantities of kola nuts are given to the chiefs of the quartiers and the traditional healers as a sign of respect.

These individual meetings are followed by a larger community meeting attended by the above personages as well as numerous other local health care providers, traditional healers and notable citizens (including several respected women from the community). Planned studies are explained in more detail, and ample time is given for carefully and thoroughly addressing all questions and concerns. This question and answer period is frequently prolonged with many detailed and often sophisticated questions being raised. Each presentation, question and response is translated from French into Dogon and Peulh so that all present understand the entire discourse.

Once this group of community leaders has expressed their approval of the planned study, they disseminate information to their various constituencies, so that when potential recruits are approached by study staff they are already generally aware of the nature of the impending study. The investigators do not consider this process to constitute "community consent" in addition to or in lieu of individual informed consent, but rather a community "permission to enter" that is a necessary prerequisite to conducting any study in a tight-knit and highly organized traditional rural community such as Bandiagara.

After community meetings have been held, a brief announcement is made on local radio describing the study. Prior to initiating screening and informed consent, the study team meets to review the oral translation of the consent forms into the relevant local languages and dialects word by word, until there is consensus that the individuals responsible for giving consent in each language are conveying as accurately as possible the exact content of the IRB-approved French language consent form.

A period of approximately two weeks is allotted for screening and recruitment to allow plenty of time for participants to consider their decision about participating and to discuss their participation with family members and others in the community. At the times of screening and recruitment, the consent forms are read to participants who speak French, and translated orally into the language of choice of each participant. In all cases, the investigator will give the participants ample opportunity to inquire about the details of the study and to ask any questions before dating and signing the consent forms, including the opportunity to take a copy of the consent form home to review with family members or others before returning on a later day with their decision. All illiterate individuals will have the study and consent forms explained to them point by point by the interviewer in the presence of a witness who will sign the consent form. Witnesses will have no association with the conduct of the study and will not be related to the study subject.

We will adapt a comprehension test recently piloted in another vaccine trial in Mali. Volunteers must answer all questions on the comprehension exam correctly prior to being eligible for enrollment to the study. Study staff will use incorrect answers to identify those areas of the informed consent that need further review with the volunteer. The incorrectly answered questions will then be repeated to the volunteer after a review of the informed consent. A final score of 100% will be required for the volunteer to be considered eligible to continue the screening process. This test will be administered orally in the presence of a witness in the case of potential volunteers who cannot read.

Informed consent will be documented by the use of a written consent form approved by the IRBs and signed or thumbprinted and dated by the participant, and by the person who conducted the informed consent discussion. Thumb printing will be used for illiterate persons, who are expected to constitute the majority of participants. The consent will be orally translated into native languages from the French written version of the consent form. The consent will be administered by a study clinician who is fluent in French and will either be fluent in the local language of the participant or use a translator. A witness will assist during the procedure. After the participant clearly states that she/he has understood what was explained and agrees to participate to the study, the consent forms will be completed. The participant will be asked if

she/he prefers to thumbprint or to sign. In the case of the thumbprint option, the distal end of her/his left thumb will be applied to a stamp inker and then firmly applied to the space on the consent forms reserved for thumbprints. This procedure has been followed for many years by the BMP team, and thumbprints are uniformly legible.

The signature/thumbprint confirms that the consent is based on information that has been understood. Each participant's signed informed consent form is kept on file by the investigator for possible inspection by regulatory authorities. The subject will receive a copy of the signed and dated written informed consent forms and any other written information provided by the investigator, and will receive copies of any signed and dated consent form updates and any amendments to the written information.

Since the vast majority of study participants do not use telephones, fax or mail, contact information is provided in terms of local physicians who can be visited directly and who can themselves reach the investigators directly or by telephone or fax.

15.2.2 Screening recruitment radio announcement text

"The Bandiagara Research Project team from the Faculty of Medicine in Bamako has returned to Bandiagara, and sends its greetings to the population of Bandiagara. The team is here to test an experimental malaria vaccine, to see if it is safe to use in adults who live in a place where they get malaria. Adult men and women aged 18-55 years who live in Bandiagara town and are interested in participating in this research study are invited to come to the Bandiagara Health Center at [*time*] on [*date*] to learn more about this study**."**

15.3 Role of Local Medical Monitor

Prof. Hamar Traoré will be the Local Medical Monitor for this study. The term Local Medical Monitor is equivalent to the ICH term "Sponsor's Medical Expert". Prof Traoré's *curriculum vitae* will be maintained on record. He is a qualified and experienced internal medicine physician not otherwise associated with this protocol, who is able to provide medical care to research subjects for conditions that may arise during the conduct of the study and oversee the safety aspects of the study. The medical monitor is required to review all serious adverse events associated with the protocol and provide an unbiased written report of the event within 10 calendar days of the initial report. At a minimum, the local medical monitor should comment on the outcomes of the serious adverse event (SAE) and relationship of the SAE to the test product. The medical monitor should also indicate whether he concurs with the details of the report provided by the study investigator.

The Local Medical Monitor will support the clinical investigators and act as a link between the investigators and the Safety Monitoring Committee (SMC).

The PI will report all serious adverse events to the Local Medical Monitor. He will review all serious adverse events associated with the protocol and will provide an unbiased written report of the event within 10 calendar days of the initial report. At a minimum, this report will comment on the outcomes of the adverse event and relationship to vaccination and indicate if the Local Medical Monitor concurs with the details of the report provided by the investigators.

The involvement of the Local Medical Monitor will be particularly important when decisions related to safety of participants have to be made quickly. Code break envelopes will be in his safekeeping and he may unblind individual study participants if deemed necessary for medical and/or ethical reasons. In exceptional circumstances, for example a death possibly related to vaccination, he would have the authority to suspend the whole or any specific aspect of the trial pending review by the SMC.

Prof. Traoré will be on-site during active phases of immunization and during the immediate post-vaccination surveillance period. A physician on staff at the Bandiagara Health Center who resides full time in Bandiagara will act as the on-site local Medical Monitor in support of Prof. Traoré between the vaccinations.

The Local Medical Monitor's role will include:

- Acting as the study volunteers' advocate
- Promptly communicating relevant safety information to the SMC
- Providing advice to the investigators on whether a set of clinical circumstances in a study warrants formal notification to the USAMMDA, and the SMC.
- Providing clinical advice on any illness in study subjects especially in circumstances in which treatment might influence the course of the trial.
- Review all SAEs as outlined above

The Local Medical Monitor will liaise closely with the PI throughout the course of the trial and relay relevant safety information to the PI and the SMC. The PI and co-PI will subsequently inform the FMPOS IRB, the SMC, DMID, GSKBio, WRAIR and USAMMDA of safety concerns that arise.

15.4 Safety Monitoring Committee (SMC)

An independent Safety Monitoring Committee will be constituted to help the Medical Monitor review safety data in real time. This committee will consist of the Local Medical Monitor and at least 2 other independent experts, one an experienced senior Malian physician, and the other an expert on conducting and/or monitoring clinical trials. The role of the SMC will be to provide safety oversight over the conduct of the trial. It will review safety data between immunizations and advise on progression to the next immunization. The Safety Monitoring Committee will hold three conference calls to review the safety data generated from the trial up to that point. Each will occur approximately two to three weeks after each immunization with the 25 μg dose level. The purpose of these conference calls will be to review the accumulated safety data in order to advise whether or not the study can proceed to the subsequent immunization with the 50 μg dose level specifically, and whether the trial can proceed generally. The study will not proceed to the next immunization unless explicitly agreed to by a majority of the members of the Safety Monitoring Committee, either in the form of a letter, fax or email from the SMC chair. In the event that the SMC identifies a safety concern, the collaborative group including the investigators, DMID, WRAIR and GSK will review all safety data in order to decide whether to give the next sequential dose. Senior investigators and representatives from DMID, WRAIR, GSK and USAMMDA will be invited to but not obligated to participate in the conference calls during open sessions, which will be followed by closed sessions including only SMC members.

The investigator will inform the SMC of:

- All subsequent protocol amendments, informed screening or study consent form changes or revisions of other documents originally submitted for review
- Serious adverse events (SAEs) and grade 3 adverse experiences (as defined in Table 5, section 10.2) occurring during the study, regardless of relationship to the study vaccine
- New information that may affect adversely the safety of the subjects or the conduct of the study.

The SMC will be empowered to put the study on temporary hold pending review of potential safety issues. The SMC will request additional information from the Principal Investigator as needed and will request any appropriate statistical calculations to support discussions with the sponsor. All documentation provided to members of the SMC for information and review will be treated in a confidential manner.

15.5 Risks and potential benefits to the participants

15.5.1 Vaccination

Risks associated with both vaccinations include local inflammatory reactions to the injected product, such as injection site pain and swelling and some limitation of arm movement. Systemic effects may include flu-like syndrome, fever, chills, nausea/GI symptoms, headache, malaise, myalgia and arthralgia. While rare, allergic reactions, including life-threatening anaphylaxis, are associated with many vaccine preparations and must therefore be considered as a potential risk in this study. Risks associated with drawing blood include fainting, infection and bruising.

15.5.2 Medical treatment for participants

Free medical treatment will be provided to enrolled participants during the active immunization phase and the surveillance period, at a level that meets or exceeds the local Malian standards of medical diagnosis and treatment. Medical care for ailments not related to vaccination will not extend beyond the study period. Medical care for ailments related to vaccination will extend, at minimum, until the condition has resolved or stabilized.

15.5.3 Rabies vaccination

During the conduct of the study participants randomized to receive rabies immunization will benefit from this due to the assumed prevalence of rabies in Bandiagara. At the end of the study all participants will be informed of the vaccine they received. Volunteers randomized to the FMP2.1/AS02A vaccine will be offered rabies immunization at that time. This will be done at the recommended schedule of 0, 7 and 21 days.

15.5.4 Pregnancy

The effects of both the study and comparator vaccines on the unborn fetus are unknown. Female participants will be counseled to avoid becoming pregnant during the immunization phase of the study and up to one month after the last immunization. Any female participant interested in contraceptive methods will be referred to the local health center family planning services for evaluation and institution of one of several available contraceptive method approved by the Malian Ministry of Health.

15.5.5 Benefits

Participants may not receive any direct benefit from the experimental vaccine. However, they will receive follow-up medical care during the screening period as well as the 12 months of the study, at the BMP Clinic in Bandiagara. At the end of the study, participants will be told what vaccine they received. If they received the malaria vaccine, they may come to the clinic

after the study to get the rabies vaccine, if they wish, so that all participants may potentially benefit from immunization against rabies.

15.6 Precautions to minimize risks

15.6.1 Vaccination

As outlined above, the participants will be monitored closely during their participation in this study. Both the study antigen and adjuvant have been prepared according to Good Manufacturing Practices (GMP). The vaccine will be administered by experienced investigators trained in Good Clinical Practices (GCP) with drugs and equipment available for the treatment of anaphylaxis. All study immunizations will be given by intramuscular injection.

15.6.2 Post-vaccination

The vaccinees will be observed closely for at least 30 minutes, with appropriate medical treatment readily available in case of a rare anaphylactic reaction following the administration of vaccines.

Senior investigators will administer the vaccines in the BMP clinic. The vaccinating investigators will not be directly involved in post-immunization assessment of adverse events. A physician skilled and familiar with emergency resuscitation procedures will assist during the immunization and post-immunization observation phases. In order to maintain the study blinding, the vaccines will be prepared for administration by a specific team that will not be involved in further participant evaluation during surveillance.

Drugs to treat anaphylaxis include epinephrine diphenhydramine and methylprednisolone. Epinephrine will be injected parenterally in standard recommended doses. Diphenhydramine will be administered orally or parenterally in standard recommended doses and methylprednisolone will be injected parenterally as needed to treat anaphylaxis. A kit containing necessary supplies for the management of anaphylaxis will be on-site. The Investigators will be trained and familiarized with resuscitation procedures, following detailed SOPs. A physician skilled and familiar with emergency resuscitation will assist on-site at each immunization phase.

15.6.3 Post-trial information on product safety

For domestic trials, the U.S. Army Medical Research and Materiel Command retains data sheets including name, study number, address (when one is available) and dates for all volunteers participating in research for entry into the Command's Volunteer Registry Data Base. The intent of the data base is to readily answer questions concerning an individual's participation in research sponsored by USAMRMC and to ensure that the USAMRMC can exercise its obligation to ensure research volunteers are adequately warned (duty to warn) of risks and to provide new information as it becomes available. The information is stored at USAMRMC for a minimum of 75 years.

In the case of this trial, there is no likelihood that the rural Malian study participants would contact USAMRMC with questions, nor is it feasible for USAMRMC to directly contact study participants with new information about the study products. Instead, a Volunteer Registry Database containing the above information will be maintained in a locked facility at the Malaria Research and Training Center, under the control of the director of the Biostatistics and Epidemiology Unit, for as long as the institution exists. In case the need arises to inform study subjects of new information about the study product, USAMRMC can contact senior MRTC

investigators, who may be able to trace the study participants through local contacts at the study site. Access to this Volunteer Registry Database will be subject to the same confidentiality protections as CRFs and other study-related documents bearing personal information.

15.6.4 Protection of study staff

All study personnel have been trained to follow current Universal Precautions as recommended by the U.S. Centers for Disease Control and Prevention. Additionally, the following approved SOPs from the BMP clinical lab elaborate the precautions that will be taken by study personnel to minimize risks: General Laboratory Safety, Exposure to Blood and Infectious Material, and Waste Management.

15.7 Procedures for maintaining confidentiality

Participants will be assigned a unique study number. All results will be keyed to this number. Study records will only be available to staff members and will be kept locked at the study site conforming to the investigators' SOPs. Following the conclusion of the study, all records will be maintained on site for a minimum of two years, after which they will be stored long-term in the MMVDU data storage facilities in Bamako. All records will be retained in locked metal boxes for at least two years after a marketing application is approved for FMP2.1; or, if an application is not approved for FMP2.1, until two years after shipment and delivery of the drug for investigational use is discontinued and the FDA has been so notified. After either of these conditions have been met with permission of the sponsors, records will be destroyed. Representatives of the US Army Medical Research and Material Command (USAMRMC), the FDA and the sponsors may review these records.

15.8 Compensation

Each participant will be compensated for the time they donate to the study by being given 50 kg of rice and 50 kg of millet, total value about $60. One half will be provided after the first immunization and one half at the end of the study. Throughout Mali, the availability of food is subject to seasonal variation in relation to the harvest season. However, there is no recent history of famine or starvation. At the study site, while cases of pediatric malnutrition are occasionally seen at the Health Centre, these are usually attributable to poor feeding habits rather than to scarcity of food, and the treatment is educating the parents to provide more nutritional foods to small children. The total amount of food to be distributed in two parts over the course of one year will last an average family approximately four weeks. The type of food distributed, i.e., rice and millet, are staple starches that are typically served accompanied by a sauce containing some sort of meat as well as vegetables, and are therefore only a part of the local diet. This amount of compensation is consistent with what we have provided to participants of longitudinal studies in Mali for several years, and has been carefully considered by the local Malian IRB, who have determined that it is appropriate compensation for time lost to study procedures, and not coercive.

As would be the case for study volunteers being compensated for study participation in the U.S., study participants who drop out of the study voluntarily will have their compensation prorated for their time in the study. Study participants who must be withdrawn due to adverse events or other non-voluntary reasons will receive the full compensation.

15.9 Financing and insurance

This study will be financed primarily by contract N01-AI-85346 from the DMID, National Institutes of Health, to the University of Maryland with a subcontract to the University of Bamako. Additional resources are provided by the intramural MVDB, National Institutes of Health. These additional resources are primarily in the form of infrastructure including vehicles, communications, computer networks, as well as the training, preparation and equipping of the clinical laboratory.

GSK Biologicals has and will maintain during the term of its Material Transfer Agreement with the University of Bamako or the Protocol, whichever is the longer, a clinical trial liability insurance policy sufficient to cover the cost of reasonable medical care required to treat or stabilize adverse reactions suffered by patients who received FMP2.1 adjuvanted with AS02A in accordance with the approved Protocol, to the extent the medical care is not covered by the patients' medical or hospital insurance or by third party or governmental programs providing such coverage.

16 REFERENCES

(1) Miller LH, Baruch DI, Marsh K, Doumbo OK. The pathogenic basis of malaria. Nature 2002; 415(6872):673-679.

(2) Chitnis CE. Molecular insights into receptors used by malaria parasites for erythrocyte invasion. Curr Opin Hematol 2001; 8(2):85-91.

(3) Dvorak JA, Miller LH, Whitehouse WC, Shiroishi T. Invasion of erythrocytes by malaria merozoites. Science 1975; 187(4178):748-750.

(4) Aikawa M, Miller LH, Johnson J, Rabbege J. Erythrocyte entry by malarial parasites. A moving junction between erythrocyte and parasite. J Cell Biol 1978; 77(1):72-82.

(5) Snow RW, Craig M, Deichmann U, Marsh K. Estimating mortality, morbidity and disability due to malaria among Africa's non-pregnant population. Bull World Health Organ 1999; 77(8):624-640.

(6) Alonso PL, Armstrong JR, Lindsay SW. Malaria, bednets, and mortality. Lancet 1991; 338(8771):897.

(7) Nevill CG, Some ES, Mung'ala VO, Mutemi W, New L, Marsh K et al. Insecticide-treated bednets reduce mortality and severe morbidity from malaria among children on the Kenyan coast. Trop Med Int Health 1996; 1(2):139-146.

(8) D'Alessandro U, Olaleye BO, McGuire W, Langerock P, Bennett S, Aikins MK et al. Mortality and morbidity from malaria in Gambian children after introduction of an impregnated bednet programme. Lancet 1995; 345(8948):479-483.

(9) Brooker S, Guyatt H, Omumbo J, Shretta R, Drake L, Ouma J. Situation analysis of malaria in school-aged children in Kenya - what can be done? Parasitol Today 2000; 16(5):183-186.

(10) Sachs J, Malaney P. The economic and social burden of malaria. Nature 2002; 415(6872):680-685.

(11) Coulibaly D, Diallo DA, Thera MA, Dicko A, Guindo AB, Kone AK et al. Impact of preseason treatment on incidence of falciparum malaria and parasite density at a site for testing malaria vaccines in Bandiagara, Mali. Am J Trop Med Hyg 2002; 67(6):604-610.

(12) Diallo D, Traore AK, Baby M, Rhaly AA, Bellis G, Chaventre A. Haemoglobinopathies C and S in the Dogons. Nouvelle Revue Francaise d Hematologie 1994; 35(6):551-554.

(13) Agarwal A, Guindo A, Cissoko Y, Taylor JG, Coulibaly D, Kone A et al. Hemoglobin C associated with protection from severe malaria in the dogon of mali, a west african population with a low prevalence of hemoglobin S [In Process Citation]. Blood 2000; 96(7):2358-2363.

(14) Dame JB, Williams JL, McCutchan TF, Weber JL, Wirtz RA, Hockmeyer WT et al. Structure of the gene encoding the immunodominant surface antigen on the sporozoite of the human malaria parasite Plasmodium falciparum. Science 1984; 225(4662):593-599.

(15) Cerami C, Frevert U, Sinnis P, Takacs B, Clavijo P, Santos MJ et al. The basolateral domain of the hepatocyte plasma membrane bears receptors for the circumsporozoite protein of Plasmodium falciparum sporozoites. Cell 1992; 70(6):1021-1033.

(16) Ballou WR, Hoffman SL, Sherwood JA, Hollingdale MR, Neva FA, Hockmeyer WT et al. Safety and efficacy of a recombinant DNA Plasmodium falciparum sporozoite vaccine. Lancet 1987; 1(8545):1277-1281.

(17) Herrington DA, Clyde DF, Losonsky G, Cortesia M, Murphy, JR et al. Safety and immunogenicity in man of a synthetic peptide malaria vaccine against Plasmodium falciparum sporozoites. Nature 1987; 328(6127):257-259.

(18) Fries LF, Gordon DM, Richards RL, Egan JE, Hollingdale MR, Gross M et al. Liposomal malaria vaccine in humans: a safe and potent adjuvant strategy. Proc Natl Acad Sci U S A 1992; 89(1):358-362.

(19) Ribi E, Cantrell J, Feldner T. Microbiology. Washington, DC: American Society of Microbiology, 1986.

(20) Hoffman SL, Edelman R, Bryan JP, Schneider I, Davis J, Sedegah M et al. Safety, immunogenicity, and efficacy of a malaria sporozoite vaccine administered with monophosphoryl lipid A, cell wall skeleton of mycobacteria, and squalane as adjuvant. Am J Trop Med Hyg 1994; 51(5):603-612.

(21) Vreden SG, Verhave JP, Oettinger T, Sauerwein RW, Meuwissen JH. Phase I clinical trial of a recombinant malaria vaccine consisting of the circumsporozoite repeat region of Plasmodium falciparum coupled to hepatitis B surface antigen. Am J Trop Med Hyg 1991; 45(5):533-538.

(22) Gordon DM, McGovern TW, Krzych U, Cohen JC, Schneider I, LaChance R et al. Safety, immunogenicity, and efficacy of a recombinantly produced Plasmodium falciparum circumsporozoite protein-hepatitis B surface antigen subunit vaccine. J Infect Dis 1995; 171(6):1576-1585.

(23) Bomford R, Stapleton M, Winsor S, Beesley JE, Jessup EA, Price KR et al. Adjuvanticity and ISCOM formation by structurally diverse saponins. Vaccine 1992; 10(9):572-577.

(24) Stoute JA, Slaoui M, Heppner DG, Momin P, Kester KE, Desmons P et al. A preliminary evaluation of a recombinant circumsporozoite protein vaccine against Plasmodium falciparum malaria. RTS,S Malaria Vaccine Evaluation Group [see comments]. N Engl J Med 1997; 336(2):86-91.

(25) Stoute JA, Kester KE, Krzych U, Wellde BT, Hall T, White K et al. Long-term efficacy and immune responses following immunization with the RTS,S malaria vaccine. J Infect Dis 1998; 178(4):1139-1144.

(26) Kester KE, McKinney DA, Tornieporth N, Ockenhouse CF, Heppner DG, Hall T et al. Efficacy of recombinant circumsporozoite protein vaccine regimens against experimental Plasmodium falciparum malaria. J Infect Dis 2001; 183(4):640-647.

(27) Bojang KA, Milligan PJ, Pinder M, Vigneron L, Alloueche A, Kester KE et al. Efficacy of RTS,S/AS02 malaria vaccine against Plasmodium falciparum infection in semi-immune adult men in The Gambia: a randomised trial. Lancet 2001; 358(9297):1927-1934.

(28) Good MF, Kaslow DC, Miller LH. Pathways and strategies for developing a malaria blood-stage vaccine. [Review] [94 refs]. Annual Review of Immunology 1998; 16:57-87.

(29) Narum DL, Thomas AW. Differential localization of full-length and processed forms of PF83/AMA-1 an apical membrane antigen of Plasmodium falciparum merozoites. Mol Biochem Parasitol 1994; 67(1):59-68.

(30) Crewther PE, Culvenor JG, Silva A, Cooper JA, Anders RF. Plasmodium falciparum: two antigens of similar size are located in different compartments of the rhoptry. Exp Parasitol 1990; 70(2):193-206.

(31) Waters AP, Thomas AW, Deans JA, Mitchell GH, Hudson DE, Miller LH et al. A merozoite receptor protein from Plasmodium knowlesi is highly conserved and distributed throughout Plasmodium. J Biol Chem 1990; 265:17974-17979.

(32) Kocken CH, van der Wel AM, Dubbeld MA, Narum DL, van de Rijke FM, Van Gemert GJ et al. Precise timing of expression of a Plasmodium falciparum-derived transgene in Plasmodium berghei is a critical determinant of subsequent subcellular localization. J Biol Chem 1998; 273(24):15119-15124.

(33) Deans JA, Alderson T, Thomas AW, Mitchell GH, Lennox ES, Cohen S. Rat monoclonal antibodies which inhibit the in vitro multiplication of Plasmodium knowlesi. Clin Exp Immunol 1982; 49(2):297-309.

(34) Thomas AW, Deans JA, Mitchell GH, Alderson T, Cohen S. The Fab fragments of monoclonal IgG to a merozoite surface antigen inhibit Plasmodium knowlesi invasion of erythrocytes. Mol Biochem Parasitol 1984; 13(2):187-199.

(35) Peterson MG, Marshall VM, Smythe JA, Crewther PE, Lew A, Silva A et al. Integral membrane protein located in the apical complex of Plasmodium falciparum. Mol Cell Biol 1989; 9(7):3151-3154.

(36) Waters AP, Thomas AW, Deans JA, Mitchell GH, Hudson DE, Miller LH et al. A merozoite receptor protein from Plasmodium knowlesi is highly conserved and distributed throughout Plasmodium. J Biol Chem 1990; 265(29):17974-17979.

(37) Marshall VM, Zhang L, Anders RF, Coppel RL. Diversity of the vaccine candidate AMA-1 of Plasmodium falciparum. Mol Biochem Parasitol 1996; 77(1):109-113.

(38) Oliveira DA, Udhayakumar V, Bloland P, Shi YP, Nahlen BL, Oloo AJ et al. Genetic conservation of the Plasmodium falciparum apical membrane antigen-1 (AMA-1). Mol Biochem Parasitol 1996; 76(1-2):333-336.

(39) Hodder AN, Crewther PE, Matthew ML, Reid GE, Moritz RL, Simpson RJ et al. The disulfide bond structure of Plasmodium apical membrane antigen-1. J Biol Chem 1996; 271(46):29446-29452.

(40) Thomas AW, Waters AP, Carr D. Analysis of variation in PF83, an erythrocytic merozoite vaccine candidate antigen of Plasmodium falciparum. Mol Biochem Parasitol 1990; 42(2):285-287.

(41) Escalante AA, Grebert HM, Chaiyaroj SC, Magris M, Biswas S, Nahlen BL et al. Polymorphism in the gene encoding the apical membrane antigen-1 (AMA-1) of Plasmodium falciparum. X. Asembo Bay Cohort Project. Mol Biochem Parasitol 2001; 113(2):279-287.

(42) Kocken CH, Narum1 DL, Massougbodji A, Ayivi B, Dubbeld MA, van der WA et al. Molecular characterisation of Plasmodium reichenowi apical membrane antigen-1 (AMA-1), comparison with P. falciparum AMA-1, and antibody-mediated inhibition of red cell invasion. Mol Biochem Parasitol 2000; 109(2):147-156.

(43) Thomas AW, Trape JF, Rogier C, Goncalves A, Rosario VE, Narum DL. High prevalence of natural antibodies against Plasmodium falciparum 83-kilodalton apical membrane antigen (PF83/AMA-1) as detected by capture-enzyme-linked immunosorbent assay using full-length baculovirus recombinant PF83/AMA-1. Am J Trop Med Hyg 1994; 51(6):730-740.

(44) Udhayakumar V, Kariuki S, Kolczack M, Girma M, Roberts JM, Oloo AJ et al. Longitudinal study of natural immune responses to the Plasmodium falciparum apical membrane antigen (AMA-1) in a holoendemic region of malaria in western Kenya: Asembo Bay Cohort Project VIII. Am J Trop Med Hyg 2001; 65(2):100-107.

(45) Dolo A, Thera MA, Baby M. Strain-specific antibody responses to potential malaria vaccine antigens in Mali. Am J Trop Med Hyg 69[3], 495. 2003.
Ref Type: Abstract

(46) Hodder AN, Crewther PE, Anders RF. Specificity of the protective antibody response to apical membrane antigen 1. Infect Immun 2001; 69(5):3286-3294.

(47) Wu JY, Gardner BH, Murphy CI, Seals JR, Kensil CR, Recchia J et al. Saponin adjuvant enhancement of antigen-specific immune responses to an experimental HIV-1 vaccine. J Immunol 1992; 148(5):1519-1525.

(48) Soltysik S, Bedore DA, Kensil CR. Adjuvant activity of QS-21 isomers. Ann N Y Acad Sci 1993; 690:392-395.

(49) Myers KR, Truchot AT, Ward J. Cellular and molecular aspects of endotoxin reactions. In: Nowotny A, editor. Amsterdam: 1990: 145-156.

(50) Loppnow H, Durrbaum I, Brade H, Dinarello CA, Kusumoto S, Rietschel ET et al. Lipid A, the immunostimulatory principle of lipopolysaccharides? Adv Exp Med Biol 1990; 256:561-566.

(51) Johnson AG, Tomai MA. A study of the cellular and molecular mediators of the adjuvant action of a nontoxic monophosphoryl lipid A. Adv Exp Med Biol 1990; 256:567-579.

(52) Dreesen DW, Fishbein DB, Kemp DT, Brown J. Two-year comparative trial on the immunogenicity and adverse effects of purified chick embryo cell rabies vaccine for pre-exposure immunization. Vaccine 1989; 7(5):397-400.

(53) Nicholson KG, Farrow PR, Bijok U, Barth R. Pre-exposure studies with purified chick embryo cell culture rabies vaccine and human diploid cell vaccine: serological and clinical responses in man. Vaccine 1987; 5(3):208-210.

(54) Vodopija I, Sureau P, Lafon M, Baklaic Z, Ljubicic M, Svjetlicic M et al. An evaluation of second generation tissue culture rabies vaccines for use in man: a four-vaccine comparative immunogenicity study using a pre-exposure vaccination schedule and an abbreviated 2-1-1 postexposure schedule. Vaccine 1986; 4(4):245-248.

(55) Wasi C, Chaiprasithikul P, Chavanich L, Puthavathana P, Thongcharoen P, Trishanananda M. Purified chick embryo cell rabies vaccine. Lancet 1986; 1(8471):40.

(56) Cox JH, Schneider LG. Prophylactic immunization of humans against rabies by intradermal inoculation of human diploid cell culture vaccine. J Clin Microbiol 1976; 3(2):96-101.

(57) Kuwert EK, Marcus I, Werner J, Iwand A, Thraenhart O. Some experiences with human diploid cell strain-(HDCS) rabies vaccine in pre- and post-exposure vaccinated humans. Dev Biol Stand 1978; 40:79-88.

(58) Ajjan N, Soulebot JP, Stellmann C, Biron G, Charbonnier C, Triau R et al. [Results of preventive rabies vaccination with a concentrated vaccine of the PM/WI38-1503-3M rabies strain cultured on human diploid cells. Preparation of mixed antirabies-antitetanus hyperimmune immunoglobulin by plasmapheresis of blood taken from vaccinated veterinary students]. Dev Biol Stand 1978; 40:89-100.

(59) Costy-Berger F. [Preventive rabies vaccination using vaccine prepared from human diploid cells]. Dev Biol Stand 1978; 40:101-104.

(60) Dreesen DW. A comparative study of cell culture rabies vaccine: Immunogenicity and safety. Protocol 8USA301RA, 1985 - 1986. 1986.
Ref Type: Unpublished Work

(61) Yapo AE. Profil biochimique de reference normal de l'enfant Ivoirien de 0 a 15 ans. Medicine d'Afrique Noire 1999; 46:4-9.

(62) How to Define and Determine Reference Intervals in the Clinical Laboratory: Approved Guidelines. Second Edition[20], C28-A2. 2000. NCCLS.
Ref Type: Serial (Book,Monograph)

17 APPENDICES

17.1 Consent forms and translation certification

CONSENT FORM TO ALLOW SCREENING TESTS August 10, 2004 v4

Project title: Double blind randomized, controlled Phase 1 dose escalation trial to evaluate the safety and immunogenicity of the WRAIR AMA1 malaria antigen (FMP2.1) adjuvanted in GSK's AS02A vs. rabies vaccine in malaria-experienced adults in Bandiagara, Mali

Principal investigator:	Mahamadou A. Thera M.D., M.P.H., Tel 223-674 0961
Co-investigators:	Christopher V. Plowe M.D. M.P.H.
	Ogobara K. Doumbo M.D., Ph.D., Tel 223-222 8109
Sponsors:	Office of the Surgeon General, U.S. Army; Division of Microbiology and Infectious Diseases, National Institute of Allergy & Infectious Diseases; University of Bamako School of Medicine; GlaxoSmithKline Biologicals, Rixensart, Belgium
Site:	Bandiagara Malaria Project Clinic, Bandiagara, Mali, West Africa

Participant name: ____

First Middle Last

Participant Screening Number: __ __ __

Purpose: Malaria is a disease that affects many people in Africa and in Mali. It is caused by germs that are spread by mosquito bites. Scientists at Walter Reed Army Institute of Research (USA) working with GlaxoSmithKline Biologicals (Belgium) have made an experimental vaccine against malaria called FMP2.1/AS02A. The Malaria Research and Training Center at the faculty of medicine in Bamako, together with his American and Belgian partners, would now like to test this malaria vaccine in adults in Bandiagara to make sure that it is safe to give to people who are regularly exposed to malaria. We invite you to take part in this research study, to have tests done to determine if you might be able to take part in the vaccine study.

Procedures: The screening may include asking you questions about your health, examining you, and doing tests on your blood and urine to look for any signs of illness in your blood, kidneys or liver. We will clean your arm before taking blood and will use a new needle that hasn't been used on anyone else. You will be told all the test results and the possible meaning of these results will be explained to you within about a week. Urine will not be stored and will be used only to find out if you can take part in this vaccine study. The amount of time that this screening visit takes should be about one or two hours. If you participate in the vaccine study, you will be vaccinated three times one month apart and checked medically frequently.

Potential benefits: The possible benefit of taking part in this screening is that you will have your health checked with the examination and tests. If anything is found that is not normal, you will be told and referred to the health care centers in Bandiagara as necessary to be checked. If your tests are normal, you may be invited to take part in the vaccine study. Potential risks: The risks to you from this screening study are minimal. Drawing blood may cause discomfort and occasional bruising at the site, and rarely, fainting occurs. We will clean your arm or finger before taking blood and will use new needles to draw the blood. The risks of donating urine are minimal.

Confidentiality: Every effort will be made to keep the results of this screening confidential. A copy of this signed consent form will be placed in our study file and a copy will be given to you.

Results may be published in medical journals or at scientific meetings but your name will not be used in the report and the specific information that we learn about you will not be shared with anybody except the authorized study investigators. Your name, your address and dates of participation in this study will be kept in a locked room at the Faculty of Medicine in Bamako, so that if there is ever a reason we need to talk to you in the future, we will be able to find you. The sponsors of the study or representatives of the U.S. Food and Drug Administration may also wish to view your records.

Right to Withdraw: Your taking part in the future study is entirely voluntary. You may refuse to take part or may withdraw from this screening program at any time without penalty or loss of care. The investigator in charge of the screening and research study may decide to end your involvement at any time for medical reasons.

Alternatives to Participation: You do not have to consent to this screening. Medical care and health examinations are available at the Health Center.

Cost and Compensation: All medical care including health examination and blood and urine tests will be provided to you without cost. Whether you decide to take part in the vaccine study or not, we will provide treatment for medical conditions that we diagnose when we perform the blood and urine testing, according to the standard of care that is available in Mali.

Consent: Do you have questions about taking part in this screening? If you have any questions or concerns about your taking part in this screening at a later date, or if you feel that you have been injured by taking part in this screening program you may contact Prof. Ogobara Doumbo in Bamako, Tel: 222 8109. If you believe that your rights are not being considered, you may contact Prof. Mamadou Marouf Keita FMPOS IRB chairman in Bamako, Tel: 223 0780/222 2712. The doctors at the Bandiagara health center and traditional medicine center can help you contact either Professors Doumbo or Keita. A copy of this consent form will be provided to you.

If you agree to participate in this study, please sign or put your thumbprint below.

_________________________ Date: _____/_____/_____

Participant signature or thumbprint *dd mm yy*

_________________________ Date: _____/_____/_____

Investigator's Signature *dd mm yy*

____

Investigator's name

Complete if participant is illiterate:

Witness to Consent Interview

On the date given next to my signature, I witnessed the "Screening Consent Interview" for the Research Study named above in this document. I attest that the information in this screening consent form and the written summary was explained to the subject, and the subject indicated that his/her questions and concerns were adequately addressed.

Name of Witness ____________________

Signature of Witness ___________________ Date: _____/_____/_____

dd mm yy

Witness to Subject's Signature

On the date given next to my signature, I witnessed the subject signs his/her name or imprint his/her thumbprint on this consent form.

Name of Witness ____________________

Signature of Witness ___________________ Date: _____/_____/_____

dd mm yy

Formulaire de consentement éclairé pour le dépistage des volontaires 10 Août, 2004 v4

Titre de l'étude : Essai thérapeutique contrôlé et randomisé en double aveugle de phase 1 pour évaluer la tolérance et l'immunogénicité de l'antigène antipaludique du WRAIR, dérivé de l'AMA1 (FMP2.1) associé à l'adjuvant AS02A de GlaxoSmithKline Biologicals en comparaison avec le vaccin antirabique dans une population adulte semi-immune à Bandiagara au Mali.

Chercheur Principal : Mahamadou A Thera, M.D., M.P.H., Tél : 223-674 0961
Chercheurs Associés : Christopher V Plowe, M.D., M.P.H.
Ogobara K Doumbo, M.D., Ph.D, Tél : 223-222 8109

Promoteurs : L'Armée américaine ; La Division de la Microbiologie et des Maladies Infectieuses (DMID) de l'Institut National des Maladies Allergiques et Infectieuses (NIAID) ; L'Université de Bamako, Faculté de médecine ; GlaxoSmithKline Biologicals. Rixesart, Belgique

Site : Bandiagara, centre de santé de référence du cercle, centre clinique du projet paludisme de Bandiagara

Nom du Participant : ____

Prénoms Nom

Numéro de dépistage du participant : __ __ __

But : Le paludisme affecte beaucoup de personnes en Afrique et au Mali. Il est causé par des germes transmis par la piqûre d'un moustique. Les scientifiques américains du Walter Reed Army Institute of Research en collaboration avec GlaxoSmithKline Biologicals de la Belgique ont mis au point un nouveau vaccin expérimental antipaludique appelé FMP2.1/ASA02A. Le centre de recherche et de formation sur le paludisme de la faculté de médecine de Bamako, (MRTC) en collaboration avec ses partenaires américains et de Belgique, veut tester ce nouveau vaccin à Bandiagara pour évaluer la tolérance du produit dans une population d'adultes en

contact naturel avec le parasite du paludisme dans le but d'évaluer si le vaccin est bien toléré dans une population naturellement exposée au risque du paludisme. Nous vous invitons à participer à ce programme de dépistage pour déterminer si vous pouvez être inclus dans l'étude.

Procédures : Au cours du dépistage nous vous poserons des questions sur votre état de santé, nous vous ferons un examen physique médical et nous ferons des tests sur l'urine et le sang pour chercher les signes d'une maladie du sang, du foie ou des reins. Pour les femmes un test de grossesse sur l'urine sera fait. Les résultats de tous ces tests ainsi que leur signification vous seront expliqués après environ une semaine. L'urine prélevée ne sera pas conservée. Elle servira seulement à déterminer si vous pouvez participer à l'étude. La durée de cette visite de dépistage sera d'environ une heure à deux heures. Au cas où vous devenez participant à l'étude du vaccin, vous recevrez trois doses de vaccin à un mois d'intervalle et vous ferez l'objet de contrôles médicaux fréquents.

Bénéfices potentiels : Le bénéfice potentiel consistera à obtenir un bilan partiel de votre état de santé basé sur les tests effectués et l'examen médical. En cas d'anomalie constatée, une explication complète vous sera fournie et votre état de santé sera pris en charge au centre de santé de référence de Bandiagara à nos frais. Si les résultats de vos tests sont satisfaisants nous vous inviterons à participer à l'étude du vaccin.

Risques potentiels : Sont minimes. La prise de sang peut provoquer un état d'inconfort et parfois, un traumatisme local. Quelques rares fois on observe une perte de connaissance. Les prélèvements seront faits après un nettoyage local, à l'aide d'aiguilles stériles et à usage unique. Une nouvelle seringue sera employée à chaque prise de sang. Les risques de collecte de l'urine sont négligeables.

Confidentialité : Les résultats de ce dépistage seront maintenus confidentiels. Une copie signée de ce formulaire de consentement, sera gardée dans nos dossiers. Et une copie signée vous sera remise. En cas de publication des résultats dans un journal médical ou de présentation lors de rencontres scientifiques votre identité ne sera pas révélée. Toute information vous concernant restera confidentielle et accessible uniquement aux chercheurs autorisés de notre équipe. Les informations permettant de vous retrouver au besoin, que sont votre nom, votre adresse et les dates de votre participation à cette étude, seront gardées sous clef à la faculté de médecine à

Bamako. Les promoteurs de l'étude ainsi que les représentants de l'agence américaine du médicament (FDA) auront accès à ces informations.

Droit de retrait : Votre participation future à l'étude du vaccin est entièrement volontaire. Vous pouvez refuser de participer à ce programme de dépistage ou de décider de vous retirer en cours de dépistage en toute liberté et à tout moment sans que cela vous cause le moindre dommage. Les responsables de l'étude pourraient aussi décider d'arrêter votre participation au programme de dépistage à tout moment pour des raisons médicales et dans votre intérêt.

Compensation : Tous les examens cliniques et tests de laboratoires, seront faits gratuitement. Que vous décidiez de participer à l'étude du vaccin ou pas vous bénéficierez de la gratuité des soins pour les affections diagnostiquées lors des tests de sang et d'urine conformément aux normes thérapeutiques en vigueur au Mali.

Alternative à la participation : Vous n'êtes pas obligé d'accepter de participer à ce programme de dépistage. Le centre de santé de Bandiagara reste disponible pour l'offre de soin et du service médical.

Consentement : Avez-vous des questions sur ce programme de dépistage ? En cas de questions ou d'inquiétudes ultérieures ou si vous estimez avoir été lésé en participant à ce programme de dépistage, vous pourriez contacter directement ou par l'intermédiaire de notre équipe à Bandiagara, le Prof Ogobara Doumbo, directeur du MRTC/DEAP à Bamako au 222 8109. Si vous estimez que vos droits n'ont pas été respectés vous pourriez vous adresser au Prof Mamadou Marouf Keita, Président du comité éthique de la FMPOS au 223 0780/222 2712. Les médecins du centre de santé de Bandiagara ou du centre de recherche en médecine traditionnelle (CRMT) peuvent aussi vous aider à contacter les Professeurs Doumbo ou Keita. Une copie de ce formulaire de consentement vous sera remise.

Si vous acceptez de participer à ce programme de dépistage, veuillez apposer votre empreinte digitale ou votre signature ci-dessous.

____________________ Date : _____/_____/_____

Empreinte digitale ou *dd mm yy*

Signature du Participant

_____________________ Date : _____/_____/_____

Signature du Chercheur *dd mm yy*

____

Nom et Prénom du Chercheur

A compléter si le participant est illettré :

Témoignage de l'interview du consentement :

Je soussigné, témoin du consentement ci-dessus pour le dépistage, atteste, qu'à la date indiquée à côté de ma signature, le contenu du formulaire de consentement pour le dépistage, a été clairement expliqué au participant et que les questions du participant ont été répondues de façon appropriée.

Nom du témoin : _______________________

Signature du témoin : ___________________ Date: _____/_____/_____

dd mm yy

Témoignage de la signature par le participant :

Je soussigné, témoin de la signature (marque par empreinte digitale) atteste qu'à la date indiquée à côté de ma signature, le participant a marqué de son empreinte digitale le formulaire de consentement pour le dépistage ci-dessus.

Nom du témoin : _______________________

Signature du témoin : ___________________ Date: _____/_____/_____

dd mm yy

RESEARCH CONSENT FORM August 10, 2004, v.4

Project title: Double blind randomized, controlled Phase 1 dose escalation trial to evaluate the safety and immunogenicity of the WRAIR AMA1 malaria antigen (FMP2.1) adjuvanted in GSK's AS02A vs. rabies vaccine in malaria-experienced adults in Bandiagara, Mali

Principal investigator:	Mahamadou A. Thera M.D., M.P.H., Tel 223-674 0961
Co-investigators:	Christopher V. Plowe M.D. M.P.H.
	Ogobara K. Doumbo M.D., Ph.D., Tel 223-222 8109
Sponsors:	Office of the Surgeon General, U.S. Army; Division of Microbiology and Infectious Diseases, National Institute of Allergy & Infectious Diseases; University of Bamako School of Medicine; GlaxoSmithKline Biologicals, Rixensart, Belgium
Site:	Bandiagara Malaria Project Clinic, Bandiagara, Mali, West Africa

Participant Name: ____

First Middle Last

Participant Study Number: __ __ __

Purpose: Malaria is a disease that affects many people in Africa and in Mali. It is caused by germs that are spread by mosquito bites. Scientists at Walter Reed Army Institute of Research (USA) working with GlaxoSmithKline Biologicals (Belgium) have made an experimental vaccine against malaria called FMP2.1/AS02A. Early tests of this vaccine in 23 people in the United States have not found any serious bad effects related to the vaccine. This vaccine is not yet approved for use in the United States or elsewhere; however, the U.S. Food and Drug Administration has permitted its use in this research study. The Malaria Research and Training Center at the faculty of medicine in Bamako, together with his American and Belgian partners, would now like to test this vaccine in adults in Bandiagara to make sure that it is safe to give to people who are regularly exposed to malaria and to find out the best dose to use. To do this, we will compare the safety of a full dose and half dose of the vaccine to a vaccine for rabies that is already known to be safe and is approved and used. Rabies is an infection of the brain that usually causes death, and can be caught from being bitten by rabid or diseased dogs or bats. We hope that information from this study can be used to develop a malaria vaccine that in the future will help protect people from getting sick with malaria.

Procedures: Your screening tests have shown that you can take part in this research study. We invite you to receive either 3 full doses of the experimental malaria vaccine, three half doses of the malaria vaccine, or RabAvert® Rabies vaccine, which is approved in Mali. Before you get your first dose of the vaccine, a card with your name and picture will be made of you to keep, so that we can be sure to identify you correctly. We will also examine you again and check your blood and urine (urine for women only). Which of the vaccines you get will be determined by chance, like when you toss a coin and you do not know whether it will land on the side with a face or on the other side. Neither you nor the doctors in the study will know which vaccine you have received. This is done to make sure that the doctors evaluate you the same way, no matter

which of the study vaccines you are given. Once the first dose of vaccine is given, the second dose will be given 30 days later and the third dose 30 days after the second one. The vaccine will be given by injection into one of your upper arms. After each injection you will be asked to stay in the clinic for 30 min for observation and come back after 1, 2, 3, 7 and 14 days for a 1-hour visit to see how you are feeling and for a brief physical examination. Following the last dose of vaccine a local guide will visit you every month for a few minutes to see if you are well. During the last six months of the study you will be asked to come to the clinic every 3 months for evaluation. About two tablespoons of blood will be taken from your arm with a needle 14 times throughout the study. This will be done to make sure that the vaccine does not hurt you and to measure the effect of the vaccine. The total amount of blood taken during the year of the study will be about 25 to 30 tablespoons. Additional blood tests may be necessary if you get sick. We will clean your arm before taking blood and will use new needles each time that blood is collected. If you become sick from malaria, you will be given treatment. Some of the blood may be stored for a long time with a number on it that tells us it is your blood, so that in the future we can do other tests related to malaria and the vaccine that we do not yet know about. The blood might be stored at the University of Bamako or at one of our partners' laboratories in the United States. Some of these tests will provide information about your body’s ability to respond to malaria. Permission from the ethical review committees at the University of Bamako and other sponsors will be gotten for any additional tests. If you don’t want your blood to be stored for other testing, please tell us so we can discard any blood that is left. If you decide at some time in the future that you wish to have your remaining blood discarded, please ask the doctors at the Health Center or Traditional Medicine Center to contact Dr. Mahamadou Thera at 2 442 495 or Pr. Ogobara Doumbo at 222 8109. You can still participate in the study whether or not you choose to have your samples stored.

Risks/Discomforts: Whether you receive rabies vaccine or malaria vaccine, you should expect soreness, swelling and, in some cases, redness at the site of injection that will disappear after 48-72 hours. In addition, muscle soreness and low-grade fever, chills, flu-like symptoms, nausea, headache, tiredness and general aches and pains are possible during the same period of time. If any of these symptoms occur, you will be offered medication to make you feel better. Like with any vaccine, there is a small possibility that you may have an allergic reaction due to vaccination. These reactions may be mild, limited to a rash, or severe and life threatening and requiring intensive medical care in a hospital. In very rare cases the rabies vaccine could cause temporary paralysis or weakness, which may be life threatening and require an extended stay in the hospital. Allergic reactions to some of the medications used in this study are also possible. Minor bruising and a slight risk of infection are possible at the site where blood is drawn. There may be other reactions that at this time are not known. If new information about the safety of the vaccine becomes available, you will be informed.

Pregnancy: Female participants should avoid becoming pregnant during the vaccination phase of the study up to one month after the last dose of vaccine. The effects of the malaria vaccine on a baby developing inside a pregnant woman are completely unknown, but may include birth defects. If you are female, a pregnancy test will be done on your urine during screening, before each vaccination, and three more times after vaccination. You will be told the results of these tests. If your pregnancy test is positive before the study starts, you will not be included in the study. If it becomes positive after your inclusion in the study, no further doses of the vaccine will be given to you. However, we will continue to check on you to see if you are well. If you wish to avoid becoming pregnant, you should practice a method of birth control. If you are interested in this please inform a member of the staff. You will then be referred to the local Health Center to

discuss the best form of birth control for your needs. However, no method of birth control is 100% effective.

Duration of study for each participant: 12 months

Benefits: You may not receive any direct benefit from being in this study. At the end of the study, we will tell you what vaccine you had. If you got the malaria vaccine you may come to the clinic after the study to get the rabies vaccine, if you want. The rabies vaccine will be given to you as three injections: the second injection will be given one week after the first, and the third injection will be given two weeks after the second.

Compensation: To make up for the time you give us to be in the study, we will give you 50 kg rice and 50 kg millet, worth about $60 We will give you half of the rice and millet after the first shot and the other half at the end of the study. In addition, you will receive follow-up medical care during the 12 months of the study, at the BMP Clinic in Bandiagara. Treatment for malaria and for other illnesses will be free of charge, according to the standard of care that is available in Mali. You will still be able to receive free medical care at the clinic even if you withdraw from the study.

Precautions to follow: Once you are enrolled into the study, with the exception of true emergencies, you should seek medical care only from the BMP Clinic in Bandiagara and you should take no medications except those given at this clinic. This should continue until you are told that the study is terminated. Please note: 1) The malaria vaccine has not been proven to prevent malaria. Therefore, you should continue to practice your regular malaria prevention methods, including sleeping under a bed net. 2) The RabAvert® Rabies vaccine does not guarantee that you are completely "protected" against rabies. Therefore, if an animal bites you, you should still see a doctor.

Number of participants in the study: 60.

Confidentiality: Information about your participation in this study will remain confidential. In any reports, participants will be referred to by study number only. Results may be published in medical journals or at scientific meetings but your name will not be used in the report and the specific information that we learn about you will not be shared with anybody except the authorized study investigators. Access to study files and your identification photograph will be limited to members of the BMP staff only and all files with information that could identify you will be kept in locked cabinets for a minimum of two years. You will receive a copy of this consent form. Your name, your address and dates of participation to this study will be kept in a locked room at the faculty of medicine in Bamako, so that if there is ever a reason we need to talk to you in the future, we will be able to find you. The sponsors of the study or representatives of the U.S. Food and Drug Administration may also wish to view your records.

Right to withdraw: Taking part in the study is entirely voluntary. You may stop taking part in this study at any time. Refusing to take part will not affect your current or future medical care in any way at the Bandiagara Health Center or Traditional Medicine Center. If you decide to withdraw from the study later, please inform any member of the BMP Clinic staff or make an appointment with the Principal Investigator listed above. You will be told about any new findings that may affect your willingness to continue in the study. Please feel free to ask any questions that will allow you to understand clearly the nature of the study, today or at any time in the future.

Alternatives to Participation: You do not have to consent to take part in this study. Medical care and health examinations are available at the Health Center, and the rabies vaccine is an approved vaccine in Mali and can be purchased if it is available.

Reasons why you might not be able to continue to take part in the study: The study doctors may decide that you should no longer take part in this study due to: 1) Health conditions that might make participation dangerous to your health; 2) Any other conditions that might make continued participation dangerous to your health.

Medical care for injury or illness: The sponsors of this study have agreed to pay for treatment necessary to help you recover from any study-related injury you may experience during the course of the study. You will not receive money as payment for any injury or illness.

Persons or places for answers in the event of a research-related injury: If you think you have a medical problem, please report to the BMP Clinic, in Bandiagara, or see one of the doctors at the Bandiagara Health Center or Traditional Medicine Center and ask them to contact one of the study doctors.

For information or answers to questions concerning your rights as a research participant, or if you feel you have been hurt by participating in this study, you may contact: The Chairman of the FMPOS Ethical Review Committee, Prof. Mamadou Marouf Keita, C/O FMPOS, BP 1805 Bamako, Mali, Tel: 223 0780/222 2712. The BMP doctors, Health Center doctors, or Traditional Medicine Center doctors can help you to contact Prof. Keita.

I do want to participate in this study:

[ ] Yes [ ] No

I agree that you may keep my blood for tests later:

[ ] Yes [ ] No

_________________________ Date: _____/_____/_____

Participant signature or thumbprint *dd mm yy*

________________________ Date: _____ /_____ /_____

Investigator's Signature *dd mm yy*

____

Investigator's Name

Complete if participant is illiterate:

Witness to Consent Interview

On the date given next to my signature, I witnessed the "Consent Interview" for the Research Study named above in this document. I attest that the information in this consent form and the written summary was explained to the subject, and the subject indicated that his/her questions and concerns were adequately addressed.

Name of Witness ___________________

Signature of Witness __________________ Date: _____/_____/_____

dd mm yy

Witness to Subject's Signature

On the date given next to my signature, I witnessed the subject sign his/her name or imprint his/her thumbprint on this consent form. Name of Witness ___________________

Signature of Witness __________________ Date: _____/_____/_____

dd mm yy

Formulaire de consentement éclairé pour participer à l'essai de Phase I du vaccin antipaludique FMP2.1/AS02A (10 Août, 2004 v4)

Titre de l'étude : Essai thérapeutique contrôlé et randomisé en double aveugle de phase 1 pour évaluer la tolérance et l'immunogénicité de l'antigène antipaludique du WRAIR, dérivé de l'AMA1 (FMP2.1) associé à l'adjuvant AS02A de GlaxoSmithKline Biologicals en comparaison avec le vaccin antirabique dans une population adulte semi-immune à Bandiagara au Mali.

**Chercheur Principal** :	Mahamadou A Thera, M.D., M.P.H. Tél : 223 674 0961
**Chercheurs Associés** :	Christopher V Plowe, M.D., M.P.H.<br>Ogobara K Doumbo, M.D., Ph.D., Tél : 223 222 8109
**Promoteurs** :	L'Armée américaine ; La Division de la Microbiologie et des Maladies Infectieuses (DMID) ; l'Institut National des Maladies Allergiques et Infectieuses (NIAID) ; L'Université de Bamako, Faculté de médecine ; GlaxoSmithKline Biologicals, Rixensart, Belgique
**Site** :	Bandiagara, centre de santé de référence du cercle, centre clinique du projet paludisme de Bandiagara

Nom du Participant : ____

Prénoms Nom

Numéro d'étude du participant : __ __ __

But : Le paludisme affecte beaucoup de personnes en Afrique et au Mali. Il est causé par des germes qui sont transmis par la piqûre d'un moustique. Les scientifiques américains et de Belgique ont mis au point un nouveau vaccin appelé FMP2.1/AS02A. Ce vaccin est expérimental et il a été administré à 23 personnes aux Etats-Unis sans que soit observé un effet secondaire grave lié au vaccin. Ce vaccin n'est pas encore autorisé sur le marché aux Etats-Unis ni ailleurs dans le monde, toutefois l'agence américaine du médicament, la FDA a autorisé son utilisation dans cette étude. Le centre de recherche et de formation sur le paludisme de la faculté de médecine de Bamako, (MRTC) en collaboration avec les chercheurs américains et de Belgique, veut tester ce nouveau vaccin à Bandiagara pour évaluer si le produit est bien toléré dans une population d'adultes en contact naturel et régulier avec le parasite du paludisme. Pour cela nous allons comparer la tolérance d'une dose complète et d'une demi-dose du FMP2.1/AS02A à celle du vaccin antirabique, un vaccin déjà connu et largement utilisé au Mali. La rage est une infection du cerveau au cours de laquelle l'issue fatale est la règle et qui est transmise par la morsure de chiens ou de chauves-souris, infectés par le germe de la rage. Nous espérons obtenir de cette étude des informations utiles pour la mise au point d'un vaccin qui protègera contre le paludisme.

Procédures : Vos tests au dépistage ont montré que vous pouvez participer à l'étude. Nous vous invitons à recevoir 3 doses de vaccins, soit le vaccin expérimental antipaludique à dose complète ou à demi-dose, soit le vaccin antirabique RabAvert® qui est autorisé sur le marché au Mali. Avant la vaccination vous recevrez une carte d'identification portant votre nom et votre photo

d'identité ; vous devez conserver cette carte et nous la présenter à chaque visite au centre. Avant la vaccination, nous vous ferons encore un examen clinique et une analyse du sang et de l'urine. L'analyse de l'urine avant la vaccination sera fait seulement chez les femmes. Le vaccin qui vous sera administré (vaccin antipaludique ou antirabique) sera déterminé par hasard comme lors d'un jet de pièce à pile ou face. Ni vous, ni les médecins de l'étude ne sauront lequel des vaccins vous a été administré. Cela est fait pour assurer que, votre état de santé sera évalué de la même façon par les médecins quel que soit le vaccin reçu. Une seconde dose sera administrée 30 jours après la première dose et une troisième dose sera administrée 30 jours après la seconde dose. Les vaccins seront administrés par injection dans le muscle des épaules. Suite à chaque injection nous vous demanderons de rester au centre clinique pour observation durant 30 minutes. Puis nous vous demanderons de venir au centre clinique le premier, le second, le troisième, le septième et le quatorzième jour après votre vaccination. Ces visites durent environ 1 heure et ont pour but de vérifier votre état de santé. Trente jours après la troisième dose du vaccin un guide local vous visitera à domicile pour évaluer votre état de santé ; puis cette visite à domicile du guide local aura lieu une fois par mois jusqu'au sixième mois, soit au total 3 visites à domicile. Après nous vous demanderons de venir en visite au centre clinique une fois au neuvième mois et une autre fois au douzième mois. Le guide local vous rappellera deux jours à l'avance de la date de votre visite au centre clinique. Environ deux cuillérées à soupe de sang vous seront prélevées à certaines visites soit 14 fois au cours de l'étude. Le sang prélevé servira à vérifier que vous tolérez bien le vaccin et à mesurer son effet sur votre organisme. La quantité totale de sang qui vous sera prélevée durant l'étude sera entre 25 et 30 cuillérées à soupe. En cas de maladie une quantité supplémentaire de sang pourra être prélevée dans un but diagnostique. En cas d'épisode de paludisme vous recevrez un traitement adéquat. Une partie de votre sang pourra être conservée avec un identifiant spécifique à vous pour de futurs tests liés au paludisme ou à un autre potentiel vaccin antipaludique. Ce sang sera conservé aux laboratoires du DEAP à Bamako ou dans un des laboratoires de nos partenaires américains aux Etats-Unis. Certains de ces tests serviront à déterminer la capacité de votre organisme à répondre face au paludisme. Mais ces tests ne seront faits qu'après approbation des comités éthiques de la FMPOS et des promoteurs. Si vous ne voulez pas que votre sang soit conservé pour des tests futurs, dîtes-le et nous ne le conserverons pas. Si vous donnez votre accord maintenant et que vous changez d'avis plus tard, contacter le Prof Ogobara Doumbo (téléphone : 222 8109) ou le Dr Mahamadou A Thera (téléphone : 244 2495) ou par l'intermédiaire des médecins de notre équipe de recherche ou par l'intermédiaire du médecin-chef du CSREF de Bandiagara ou les médecins du CRMT: alors votre sang qui était gardé sera éliminé. Vous pourrez participer à l'étude, que vous ayez accepté ou pas d'avoir votre échantillon de sang conservé. Une copie de ce formulaire de consentement vous sera remise

Risques potentiels : Que vous soyez vacciné avec le vaccin antirabique ou le vaccin expérimental antipaludique vous ressentirez de la douleur, un gonflement et parfois une rougeur au site d'injection. Ces signes vont disparaître dans les 48-72 heures suivantes. En plus une douleur musculaire, une fièvre, des frissons, des signes comme si vous aviez la grippe, une nausée, des maux de tête, de la fatigue et des douleurs dans le corps peuvent survenir. Comme pour tout vaccin vous pouvez avoir une réaction allergique au produit. Cette réaction allergique peut être mineure, limitée à un prurit ou sévère nécessitant une prise en charge urgente en soins intensifs. Très rarement le vaccin antirabique peut provoquer une paralysie ou une faiblesse temporaire qui pourrait représenter un risque vital. Des réactions allergiques sont également possibles à certains médicaments utilisés dans cette étude. La prise de sang peut provoquer un état d'inconfort et parfois une blessure locale avec un risque faible de surinfection. D'autres réactions que nous ignorons complètement à ce jour peuvent aussi se produire. Toute nouvelle

information sur la tolérance du vaccin expérimental qui nous parviendra en cours d'étude vous sera communiquée.

Grossesse : Les femmes qui participent à l'étude devront éviter une grossesse durant la phase de vaccination et cela jusqu'à un mois après la dernière dose du vaccin. Un test de grossesse sera effectué sur l'urine des femmes pendant le dépistage, avant chaque injection du vaccin et quatre fois en plus après la dernière vaccination. Vous serez informées des résultats de ces tests. Un test de grossesse positif au dépistage exclut de participer à l'étude. Un test de grossesse positif en cours d'étude exclut de recevoir des doses supplémentaires du vaccin. Toutefois si vous êtes dans une telle situation vous continuerez à être suivie pour évaluer votre état de santé. Vous pourriez éviter une grossesse pendant l'étude en adoptant une méthode de contraception appropriée. Si vous le désirez vous serez référée au centre de santé pour trouver une solution adaptée à votre situation. Toutefois, aucune méthode de contraception n'est à 100% efficace.

Durée de l'étude par participant : 12 mois

Bénéfices potentiels : Il se peut que la participation à cette étude ne vous apporte aucun bénéfice direct. A la fin de l'étude nous vous dirons quel vaccin vous aviez reçu. Si vous avez été dans le groupe du vaccin antipaludique il vous sera alors offert de bénéficier, à votre demande, du vaccin antirabique. La vaccination antirabique vous sera alors administrée en trois doses : la seconde dose vous sera administrée 1 semaine après la 1ère dose et la 3ème sera administrée 2 semaines après la seconde.

Compensation : Pour le temps consacré à l'étude vous recevrez une compensation de 50kg de riz et de 50kg de mil. Vous recevrez la moitié après la première dose de vaccin et le reste à la fin de l'étude. En plus, vous bénéficierez d'un suivi médical gratuit durant 12 mois à notre centre de recherche à Bandiagara. Toute affection diagnostiquée sera traitée à nos frais et conformément aux normes et procédures de prise en charge des maladies en vigueur au Mali. Vous continuerez à bénéficier d'une prise en charge gratuite de vos maladies même en cas de retrait avant la fin de l'étude.

Précautions à prendre : Si vous êtes inclus dans l'étude, à l'exception d'une urgence, nous vous demanderons de vous faire soigner exclusivement au niveau de notre centre clinique et de ne pas consommer d'autres médicaments en dehors de ce que nous vous donnerons. Vous devriez agir ainsi jusqu'à ce nous vous informons que l'étude est finie. Veuillez noter que le vaccin antipaludique est expérimental : nous n'avons pas la preuve qu'il protège contre le paludisme. Vous devriez donc continuer avec vos méthodes habituelles de préventions du paludisme tel que l'usage de supports imprégnés d'insecticide. Le vaccin antirabique RabAvert® ne garantit pas une protection 100% contre la rage. Par conséquent si vous êtes victimes d'une morsure suspecte vous devriez en parler au médecin.

Nombre de participants : 60

Confidentialité : Les informations vous concernant dans cette étude sont confidentielles. Dans tous les rapports seul votre numéro d'identification anonyme sera mentionné. En cas de publication des résultats dans un journal médical ou de présentation lors de rencontres scientifiques votre identité ne sera pas dévoilée. Toute information permettant de vous identifier sera gardée en sécurité sous clef. L'accès aux dossiers de l'étude et à votre photo d'identité sera limité aux chercheurs autorisés de notre équipe. Ces données seront conservées pendant au moins deux ans. Votre nom, votre adresse et les dates de votre participation à cette étude seront

conservés de façon spécifique et strictement confidentielle à Bamako, dans une pièce sécurisée du MRTC. Cette précaution permettra de vous contacter à l'avenir, dans le cas où le besoin s'imposerait. Les représentants de l'agence de l'armée américaine chargée de la recherche médicale et de la commande des matériels (USAMRMC)), de la FDA (l'agence américaine du médicament), et des promoteurs de l'étude pourront avoir accès aux dossiers pour une revue de l'étude. Vous recevrez une copie de ce formulaire de consentement.

Droit de retrait : Votre participation à cette étude est entièrement volontaire. Vous pouvez décider de vous retirer en cours d'étude en toute liberté et à tout moment sans que cela vous prive de recevoir les soins médicaux appropriés et gratuits que nécessite votre état de santé ici à Bandiagara. Si vous décidez de vous retirer en cours d'étude, nous vous prions de nous en informer ; nous discuterons toute information nouvelle pouvant affecter votre volonté de continuer ou d'arrêter votre participation à l'étude. Nous restons disponibles pour répondre à toutes vos questions concernant l'étude et pour vous apporter toutes les clarifications dont vous avez besoin, maintenant ou plus tard en cours d'étude.

Possibilité d'arrêt de votre participation et raisons : Les responsables de l'étude pourraient aussi décider d'arrêter votre participation à l'étude si cette participation compromet dangereusement votre état de santé ; ou si d'autres circonstances rendent dangereux votre participation à l'étude.

Alternative à la participation : Vous n'êtes pas obligé d'accepter de participer à cette étude. Le centre de santé de Bandiagara reste disponible pour l'offre de soin et du service médical. Et le vaccin antirabique est disponible au Mali en cas de besoin.

Soins médicaux : Toute maladie vous affectant et liée à l'étude sera entièrement et complètement prise en charge à nos frais. Vous ne recevrez pas d'argent pour une maladie ou affection provoquée par votre participation à l'étude.

Personnes-contacts : En cas de questions ou d'inquiétudes ultérieures vous pourriez contacter le centre de recherche en médecine traditionnelle (CRMT), le centre de santé de référence (CSREF) ou notre équipe à Bandiagara. Vous pourriez aussi contacter directement ou par l'intermédiaire de notre équipe à Bandiagara, le Prof Ogobara Doumbo, directeur du MRTC/DEAP à Bamako au 222 8109.
Si vous estimez que vos droits de participant ont été violés du fait de votre participation à cette étude ou si vous voulez avoir des informations supplémentaires, vous pourriez vous adresser au Prof Mamadou Marouf Keita, Président du comité d'éthique de la FMPOS au 223 0780/222 2712. Les médecins du CSREF, du CRMT et de notre équipe vous aideront au besoin à contacter le Prof Keita.

J’accepte de participer à l’étude :

[ ] Oui [ ] Non

J’accepte que mon échantillon de sang soit conservé pour des tests futurs :

[ ] Oui [ ] Non

____________________ Date : _____/_____/_____

Empreinte digitale ou *dd mm yy*

Signature du Participant

_____________________ Date : _____/_____/_____

Signature du Chercheur *dd mm yy*

____

Prénoms et Nom du Chercheur

A compléter si le participant est illettré :

Témoignage de l'interview du consentement :
Je soussigné, témoin du consentement ci-dessus pour l'étude du vaccin antipaludique FMP2.1/AS02A, atteste qu'à la date indiquée à côté de ma signature, le contenu du formulaire de consentement a été clairement expliqué au participant et que le participant a indiqué que ses questions et inquiétudes ont été répondues de façon appropriée.

Nom du témoin : ______________________

Signature du témoin : __________________ Date: _____/_____/_____
dd mm yy

Témoignage de la signature par le participant :
Je soussigné, témoin de la signature (marque par empreinte digitale) atteste qu'à la date indiquée à côté de ma signature, le participant a marqué de son empreinte digitale le formulaire de consentement pour l'étude ci-dessus.

Nom du témoin : ______________________

Signature du témoin : __________________ Date: _____/_____/_____
dd mm yy

Valable du 22/05/04 au 20/03/05

Université de Bamako
Faculté de Médecine de Pharmacie
et d'Odonto-Stomatologie (FMPOS)

Département d'Epidémiologie
des Affections Parasitaires (DEAP)
Malaria Research and Training Center (MRTC)

République du Mali
Un Peuple - Un But - Une Foi

May 15, 2004

To Whom It May Concern:

I hereby certify that the attached consent forms (screening consent and study consent) for the study "*Double blind randomized, controlled Phase 1 dose escalation trial to evaluate the safety and immunogenicity of the WRAIR AMA1 malaria antigen (FMP2.1) adjuvanted in GSK's AS02A vs. rabies vaccine in malaria-experienced adults in Bandiagara, Mali*" are accurately translated from English to French. I am a physician fluent in both English and French and have several years of experience with clinical research in Mali and with administering informed consent. I am not involved as an investigator with this study.

Signed,

Abdoulaye Touré, M.D., Ph.D.
Head of Vector-Parasite Interactions Unit/MRTC

17.2 Study comprehension exam

Double blind randomized, controlled Phase 1 dose escalation trial to evaluate the safety and immunogenicity of the WRAIR AMA1 malaria antigen (FMP2.1) adjuvanted in GSK's AS02A vs. rabies vaccine in malaria-experienced adults in Bandiagara, Mali

____________________ ______________________________

________________________ ______________________________________
Census ID # Name (first, last)

1. As part of the study, you'll be injected with a live malaria parasite…...…………..T F
2. There is a chance you could get sick from this vaccine……………..……………..T F
3. Women enrolled in this study should not become pregnant up until 1 month after the last shot…..…………………………………………………….…………………….T F
4. If you change your mind about being in the study after you are vaccinated, you can withdraw your consent……..…………………………….………………..…………..T F
5. This vaccine has been given to hundreds of people already, so we know it is completely safe……………………………………………...……….……………………….T F
6. You'll have your blood drawn as part of this study………………………….…..…T F
7. You'll get 3 vaccinations in this study……………………………………….…..…T F
8. If you feel sick during the study, you shouldn't tell anyone……………………...….T F
9. If you join the study, you need to be followed in our clinic for 12 months…..……T F
10. Everybody in this study will get the same kind of vaccine……………………….…..…T F

- Total number correct before review……………….……..……… ___
- Total number correct after review…………………………….. ___

Reviewed by ________________________________ Date ___/___/___

Volunteer signature __________________________ Date ___/___/___

Witness signature ___________________________ Date ___/___/___

17.3 USAMMDA reporting scheme of SAEs

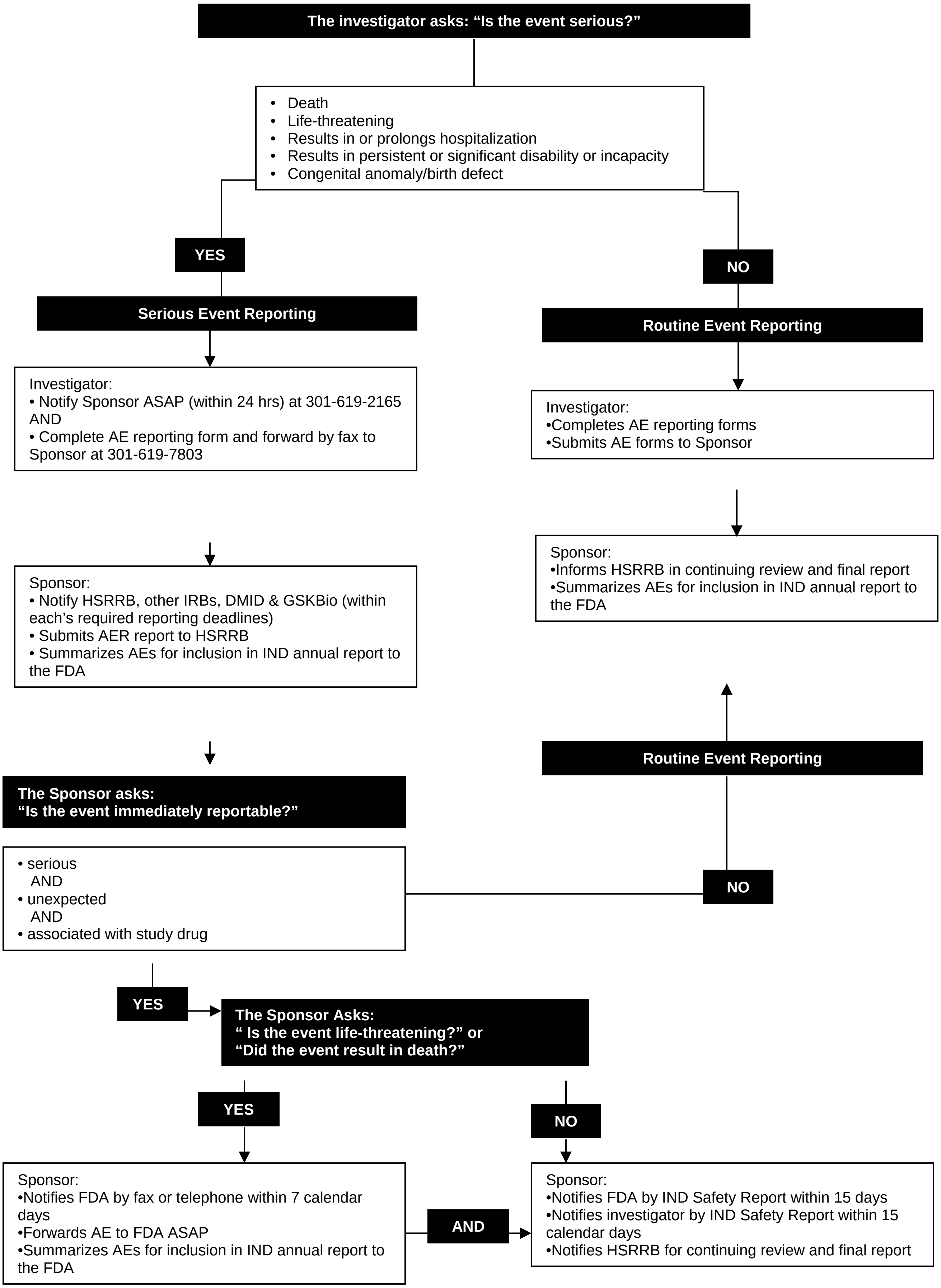

Supplement: Protocol S1 — Trial protocol (0.62 MB PDF) [file pone.0001465.s002.pdf]
